# Rare Pathogenic Variants in Mitochondrial and Inflammation-Associated Genes May Lead to Inflammatory Cardiomyopathy in Chagas Disease

**DOI:** 10.1007/s10875-021-01000-y

**Published:** 2021-03-03

**Authors:** Maryem Ouarhache, Sandrine Marquet, Amanda Farage Frade, Ariela Mota Ferreira, Barbara Ianni, Rafael Ribeiro Almeida, Joao Paulo Silva Nunes, Ludmila Rodrigues Pinto Ferreira, Vagner Oliveira-Carvalho Rigaud, Darlan Cândido, Charles Mady, Ricardo Costa Fernandes Zaniratto, Paula Buck, Magali Torres, Frederic Gallardo, Pauline Andrieux, Sergio Bydlowsky, Debora Levy, Laurent Abel, Clareci Silva Cardoso, Omar Ribeiro Santos-Junior, Lea Campos Oliveira, Claudia Di Lorenzo Oliveira, Maria Do Carmo Nunes, Aurelie Cobat, Jorge Kalil, Antonio Luiz Ribeiro, Ester Cerdeira Sabino, Edecio Cunha-Neto, Christophe Chevillard

**Affiliations:** 1 INSERM, Aix Marseille university, UMR_906, Marseille, France; 2 Theories and Approaches of Genomic Complexity (TAGC), INSERM, Aix Marseille university, UMR_1090, Parc Scientifique de Luminy, case 928, 163, avenue de Luminy, 13288 Marseille, France; 3 Laboratory of Immunology, Heart Institute (InCor), Hospital das Clínicas and Department of Medicine, Faculdade de Medicina FMUSP, Universidade de Sao Paulo, São Paulo, Brazil; 4 Institute for Investigation in Immunology, iii-INCT, Instituto Nacional de Ciência e Tecnologia, São Paulo, Brazil; 5 State University of Montes Claros (Universidade Estadual de Montes Claros), Montes Claros, Minas Gerais Brazil; 6 Unidade Clínica de Miocardiopatias, Heart Institute (Incor) Faculdade de Medicina FMUSP, Universidade de Sao Paulo, São Paulo, Brazil; 7 Departamento Morfologia, Instituto de Ciências Biológicas, Universidade Federal de Minas Gerais, Belo Horizonte, Brazil; 8 Laboratory of Human Genetics of Infectious Diseases, Necker Branch, Necker Hospital for Sick Children, Paris, France; 9 Imagine Institute, Paris Descartes University, Paris, France; 10 School of Medicine, Federal University of São João del-Rei, Divinopolis, Brazil; 11 Hospital das Clínicas and School of Medicine, Universidade Federal de Minas Gerais, Belo Horizonte, Brazil; 12 Department of Infectious Diseases, and Division of Laboratory Medicine (LIM03) and Institute of Tropical Medicine, Faculdade de Medicina FMUSP, São Paulo, Brazil; 13 Heart Institute, Laboratory of Clinical Immunology and Allergy-LIM60, University of São Paulo School of Medicine, São Paulo, SP Brazil

**Keywords:** Variants, chagas, cardiomyopathy, pathogenic, mitochondria, inflammation

## Abstract

**Abstract:**

Cardiomyopathies are an important cause of heart failure and sudden cardiac death. Little is known about the role of rare genetic variants in inflammatory cardiomyopathy. Chronic Chagas disease cardiomyopathy (CCC) is an inflammatory cardiomyopathy prevalent in Latin America, developing in 30% of the 6 million patients chronically infected by the protozoan *Trypanosoma cruzi*, while 60% remain free of heart disease (asymptomatic (ASY)). The cytokine interferon-γ and mitochondrial dysfunction are known to play a major pathogenetic role. Chagas disease provides a unique model to probe for genetic variants involved in inflammatory cardiomyopathy.

**Methods:**

We used whole exome sequencing to study nuclear families containing multiple cases of Chagas disease. We searched for rare pathogenic variants shared by all family members with CCC but absent in infected ASY siblings and in unrelated ASY.

**Results:**

We identified heterozygous, pathogenic variants linked to CCC in all tested families on 22 distinct genes, from which 20 were mitochondrial or inflammation-related – most of the latter involved in proinflammatory cytokine production. Significantly, incubation with IFN-γ on a human cardiomyocyte line treated with an inhibitor of dihydroorotate dehydrogenase brequinar (enzyme showing a loss-of-function variant in one family) markedly reduced mitochondrial membrane potential (ΔψM), indicating mitochondrial dysfunction.

**Conclusion:**

Mitochondrial dysfunction and inflammation may be genetically determined in CCC, driven by rare genetic variants. We hypothesize that CCC-linked genetic variants increase mitochondrial susceptibility to IFN-γ-induced damage in the myocardium, leading to the cardiomyopathy phenotype in Chagas disease. This mechanism may also be operative in other inflammatory cardiomyopathies.

**Supplementary Information:**

The online version contains supplementary material available at 10.1007/s10875-021-01000-y.

## Introduction

Cardiomyopathies are an important cause of cardiovascular death by heart failure and arrhythmia. Familial cardiomyopathies are a group of Mendelian genetic disorders associated with rare high-impact gene variants altering protein structure and function, mostly involving genes encoding sarcomeric/structural and calcium handling proteins. Among the acquired causes of cardiomyopathy, an estimated 30% have an infectious etiology, associated with myocarditis [1].

Little is known about the genetic underpinnings of infectious cardiomyopathy. Chagas disease (CD), caused by infection with the protozoan *Trypanosoma cruzi*, is the most common cause of nonischemic cardiomyopathy in Latin America, where 6 million people are infected, causing approximately 10,000 deaths/year due to cardiac compromise [2]. It is transmitted by the reduviid insect vector, by blood transfusion, congenitally, and by ingestion. An estimated 400,000 infected persons live in nonendemic countries, mainly the USA and Europe. Chronic CD cardiomyopathy (CCC) is a chronic inflammatory cardiomyopathy occurring decades after infection with *T. cruzi* [3] in up to 30% of CD patients. ECG abnormalities and heart conduction defects are associated with progressive inflammatory fibrotic and hypertrophic myocardial lesions including the conducting tissue [4] and precede ventricular arrhythmia/sudden cardiac death (SCD) and/or dilated cardiomyopathy with heart failure (HF), the major causes of death in CCC [5]. From the remaining CD patients, 60% persist asymptomatic form (ASY), and 10% develop gastrointestinal motility disorders [6, 7]. CD patients with ECG abnormalities typical of CCC show higher total and cardiac mortality [8]. No adequate treatment is available to prevent the development of chronic heart disease, whose prognosis is worse than dilated cardiomyopathies of other etiologies [3]. New approaches to the treatment of CCC are thus sorely needed. The pathogenesis of CCC is still incompletely understood, although myocardial inflammation and reduced mitochondrial energy metabolism are thought to play a major role in CD and cardiac remodeling and heart failure of any etiology [2, 3]. After acute infection, parasitism is partially controlled by the immune response, and low-grade parasite persistence fuels the production of inflammatory cytokines like IFN-γ and TNF-α, which is more intense in CCC than ASY patients [9]. A T cell and monocyte-rich chronic myocarditis with fibrosis and hypertrophy is the histopathological hallmark of CCC [10, 11].

IFN-γ is the most abundant cytokine expressed in the CCC myocardium, and transcriptomic analyses of the CCC myocardium show a significant IFN-γ transcriptional signature [10, 12]. IFN-γ treatment induces reduced contractility [13] and fatty acid metabolism of cardiomyocytes [14] and profibrotic changes in fibroblasts and transgenic mice overexpressing IFN-γ develop inflammatory cardiomyopathy [15, 16]. Taken together, evidence suggests IFN-γ is the culprit of CCC. In addition, the myocardium from CCC patients with ventricular dysfunction displays decreased levels of mitochondrial metabolism enzymes [17] and ATP production [18]. IFN-γ has multiple deleterious effects on the cardiomyocyte mitochondria. It induces TNF-alpha and potentiates TNF-alpha-mediated NF-kB signaling, leading to NOS2 production of NO [19, 20] which in the presence of IFN-γ-induced reactive oxygen species turns into peroxynitrite [21] and ensuing mitochondrial fragmentation and reduction of mitochondrial membrane potential, lipid beta-oxidation [14], and ATP generation [22].

High-impact rare gene variants altering protein structure and function underlie Mendelian disease and contribute to complex multifactorial disease. Approximately 10% of acute viral myocarditis (AVM) patients carried rare pathogenic homozygous variants in genes implicated in familial cardiomyopathy [23], suggesting an overlap between genetic and acquired forms of myocarditis and cardiomyopathy. However, the evidence for a role of such variants in the pathogenesis of AVM is circumstantial, as no genetic study has compared AVM patients with virus-infected patients that failed to develop myocarditis. In CD, however, serological tests can readily ascertain *T. cruzi* infection in patients who have developed cardiomyopathy or have remained asymptomatic, decades after the initial infection. This has allowed genetic association studies of common gene polymorphisms between the two *T. cruzi*-exposed groups with divergent cardiac phenotypes, CCC and ASY [2], which have a low individual effect on phenotype. CD thus provides a unique model to probe the effect of rare pathogenic genetic variants in the susceptibility towards developing postinfectious cardiomyopathy in humans.

We hypothesize here that rare genetic variants may lead to progression towards CCC by increasing cardiomyocyte susceptibility to inflammatory damage. Whole exome sequencing (WES) studies in families with multiple disease cases are an unbiased approach that has been used to identify rare pathogenic variants in Mendelian genetic disorders and complex multifactorial diseases. We used WES to search for rare, high-impact gene variants linked to CCC in nuclear families containing multiple cases of CD and involved in pathobiological processes involved in inflammatory cardiomyopathy. Our analysis disclosed rare heterozygous pathogenic variants in inflammation-related and mitochondrial genes linked to CCC cases. Functional testing indicated that IFN-γ caused significant mitochondrial dysfunction on a human cardiomyocyte line treated with an inhibitor of dihydroorotate dehydrogenase brequinar (enzyme showing a loss-of-function variant in one family).

## Patients, Materials, and Methods

### Ethical Issues

This protocol was approved by the INSERM Internal Review Board and by the Brazilian National Ethics in Research Commission (CONEP), and written informed consent was obtained from the patients. All patients enrolled in this study were over 21 years old. Investigations were conformed to the principles outlined in the declaration of Helsinki.

### Patients

Probands and their nuclear family members were recruited from the Sami-Trop CD cohort, in rural Minas Gerais state [24], and the CD outpatient clinic at the Heart Institute/HCFMUSP.

**Fig. 1:**
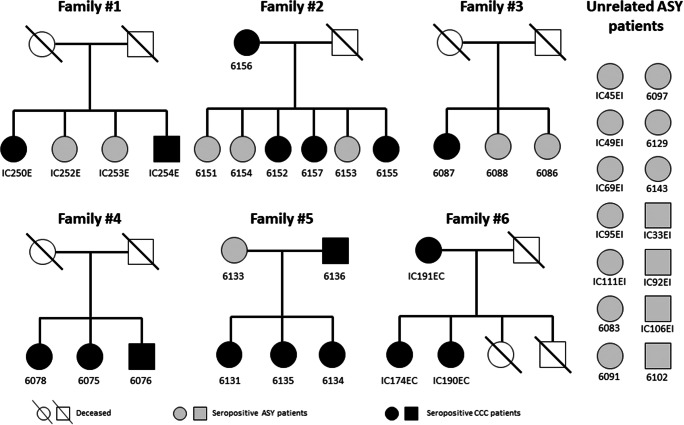
Pedigrees of the six nuclear families included in this study. All patients underwent detailed clinical interview and *T. cruzi* serological tests. *T. cruzi-*seropositive individuals were considered as CCC patients when presenting major ECG abnormalities according to the Minnesota Code classification, modified by Ribeiro et al. [8]. Major ECG abnormalities are cited in online Table 1. The presence of any one of these major ECG changes was associated with a twofold increase in mortality [8]. *T. cruzi*-seropositive individuals presenting major ECG abnormalities, according to the Minnesota Code classification, modified by Ribeiro et al. [8] were considered as CCC patients. *T. cruzi-*seropositive patients without such ECG findings, with normal echocardiography, and no clinical signs were considered indeterminate forms of CD (ASY) cases

Nuclear families typically center on a married couple which may have any number of children. Figure 1 shows the pedigrees of the 6 selected nuclear families with multiple CD cases (*n* = 25), and Table 1 depicts the clinical and demographic parameters of the studied subjects. Families 1 and 6 came from the Heart Institute, while families 2–5 came from the Sami-Trop CD cohort.

**Table 1:** Description of the study phenotypes

Family	ID	Phenotype	Chagas serology	Age	Sex	LVEF
A. Chagas disease families						
Family 1	IC250E	CCC	Seropositive	42	F	0.56
	IC252E	ASY	Seropositive	50	F	0.68
	IC253E	ASY	Seropositive	54	F	0.61
	IC254E	CCC	Seropositive	49	M	0.30
Family 2	6151	ASY	Seropositive	63	F	0.69
	6152	CCC	Seropositive	37	F	0.63
	6153	ASY	Seropositive	36	F	0.66
	6154	ASY	Seropositive	39	F	0.65
	6155	CCC	Seropositive	45	F	0.60
	6156	CCC	Seropositive	79	F	0.62
	6157	CCC	Seropositive	49	F	0.56
Family 3	6086	ASY	Seropositive	71	F	0.70
	6087	CCC	Seropositive	68	F	0.69
	6088	ASY	Seropositive	69	F	0.69
Family 4	6075	CCC	Seropositive	67	F	0.69
	6076	CCC	Seropositive	57	M	0.69
	6078	CCC	Seropositive	52	F	0.67
Family 5	6131	CCC	Seropositive	24	F	0.65
	6133	ASY	Seropositive	69	F	0.63
	6134	CCC	Seropositive	42	F	0.50
	6135	CCC	Seropositive	38	F	0.65
	6136	CCC	Seropositive	68	M	0.62
Family 6	IC174EC	CCC	Seropositive	41	F	0.38
	IC190EC	CCC	Seropositive	50	F	0.37
	IC191EC	CCC	Seropositive	69	F	0.45
				53.6 ± 14.6		0.59 ± 0.11
Seronegative siblings in family 5						
	6132	Healthy	Seronegative	32	M	0.59
	6137	Healthy	Seronegative	30	M	0.7
B. Unrelated ASY controls						
Unrelated	IC33EI	ASY	Seropositive	65	M	0.69
	IC45EI	ASY	Seropositive	51	F	0.61
	IC49EI	ASY	Seropositive	57	F	0.70
	IC69EI	ASY	Seropositive	90	F	0.68
	IC92EI	ASY	Seropositive	62	M	0.66
	IC95EI	ASY	Seropositive	57	F	0.70
	IC106EI	ASY	Seropositive	44	F	0.55
	IC111EI	ASY	Seropositive	42	F	0.63
	6083	ASY	Seropositive	41	F	0.65
	6091	ASY	Seropositive	50	F	0.62
	6097	ASY	Seropositive	47	F	0.56
	6102	ASY	Seropositive	45	M	0.66
	6129	ASY	Seropositive	71	F	0.74
	6143	ASY	Seropositive	39	F	0.66
				54.3 ± 14		0.65 ± 0.05

*LVEF*, left ventricular ejection fraction; *ASY*, asymptomatic form

### Blood DNA Preparation and Whole Exome Sequencing

On EDTA vacutainer tubes, 10 ml of blood was collected. The genomic DNA of 25 CCC and ASY patients belonging to the 6 families, plus 14 genetically unrelated ASY controls, was isolated with the QIAamp DNA Blood Midi Kit (Qiagen, Hilden, Germany). Exome sequencing was performed using the Ion Proton platform (Life Technologies, Villebon sur Yvette, France) according to a protocol previously described [25].

### Variant Prioritization

We excluded synonymous, non-exonic polymorphisms, keeping polymorphisms with a minor allele frequency (MAF) of <1% in at least one public database using the VARAFT filtering and annotation tool (https://varaft.eu) on vcf files. Variant calls with variant quality (QUAL) ≤60, depth of coverage (DP) <20, and mapping quality (MQ) ≤40 were filtered out. Only exonic nonsynonymous damaging variants were kept for downstream genetic analyses. We searched for variants, independently, in each family, under an autosomal dominant or under autosomal recessive models. In order to identify gene variants associated with CCC in each family, we selected variants that were shared by all CCC patients and absent in any and all ASY patients in a given nuclear family, as well as in 14 unrelated ASY controls. We assessed the pathogenic potential of missense variants using 4 algorithms embedded in VarAft. Only rare variants tagged as pathogenic (or damaging) were retained for downstream genetic analyses.

### Polymerase Chain Reaction and Sanger Sequencing

For validation, we designed specific primers for each mutation of interest with Primer3 software (V4.0.0) to amplify genomic DNAs (online Table 3). PCR amplifications were carried out with GoTaq polymerase (Promega, Charbonnières-les-Bains, France) and 1uM of each primer. On Eppendorf thermocycler, 50 μl reactions were carried out. The PCR products were visualized on agarose gel (1.5% agarose TBE0.5X) and purified with QIAEXII gel extraction kit (Qiagen) before Sanger sequencing.

### Mitochondrial Membrane Potential Assay

Total mitochondria were stained with MitoTracker green, and mitochondrial membrane potential (MMP) was evaluated using the TMRE dye that accumulates in fully polarized, but not in depolarized mitochondria [26]. We measured TMRE fluorescence on MitoTracker green-labeled mitochondria. Cell viability was assessed using LIVE/DEAD™ Fixable Aqua Dead Cell Stain Kit. All fluorescent dyes were from Thermo Fisher Scientific, and assays were performed using live microscopy with the ImageXpress Micro instrument (Molecular Devices).

## Results

We performed whole exome sequencing and assessed rare pathogenic gene variants associated with CCC in six nuclear families containing multiple cases of CD families (25 patients) and in a group of unrelated ASY patients (*n* = 14) who came from CD-endemic rural areas in Brazil. The average age in the CCC and ASY patients in the family groups and the ASY unrelated control were similar, around 54 ± 14 years (Table 1). Pedigrees are shown in Fig. 1.

**Fig. 2:**
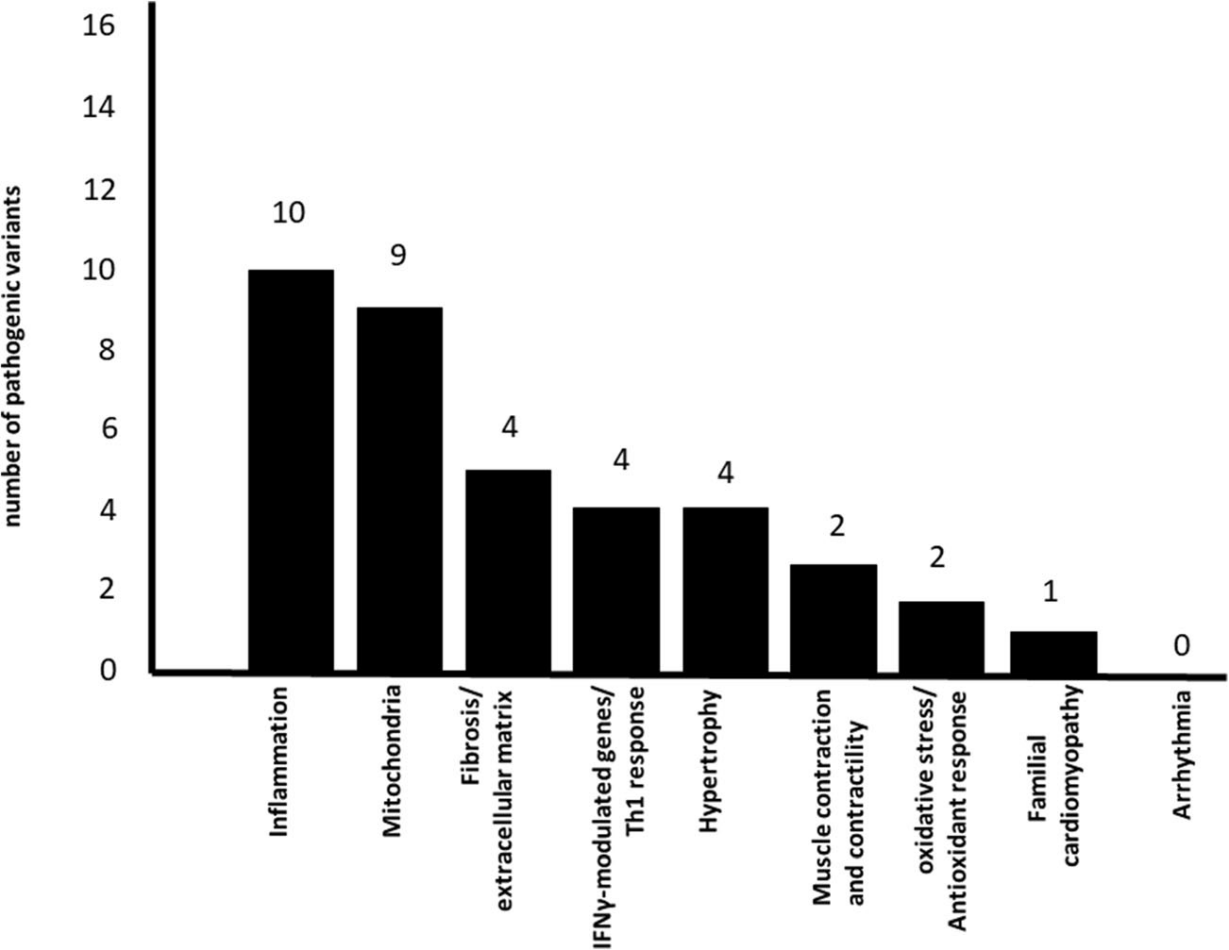
Number of genes containing pathogenic variants for each biological process related to cardiomyopathy. Pathway analyses were performed with the Ingenuity Pathways Analysis (IPA®, Qiagen, Redwood City, USA) or Reactome (reactome.org); we also interrogated whether genes participated in 9 biological processes related to the pathophysiology of CCC: inflammation, IFNγ-modulated genes/Th1 response, fibrosis/extracellular matrix, contractility of heart, hypertrophy, arrhythmia, oxidative stress/antioxidant response, familial cardiomyopathy, and mitochondria-related genes. For these nine additional biological processes, we merged IPA Knowledge Base (IKB) gene lists and published gene lists including the IFNγ induced/repressed gene lists, and Nrf2-modulated genes where appropriate. Mitochondrial gene list was a combination of genes contained in the Mitochondrion Gene Ontology term and Mitocarta 2.0. The gene list for these pathways/processes in online Table 2

**Fig. 3:**
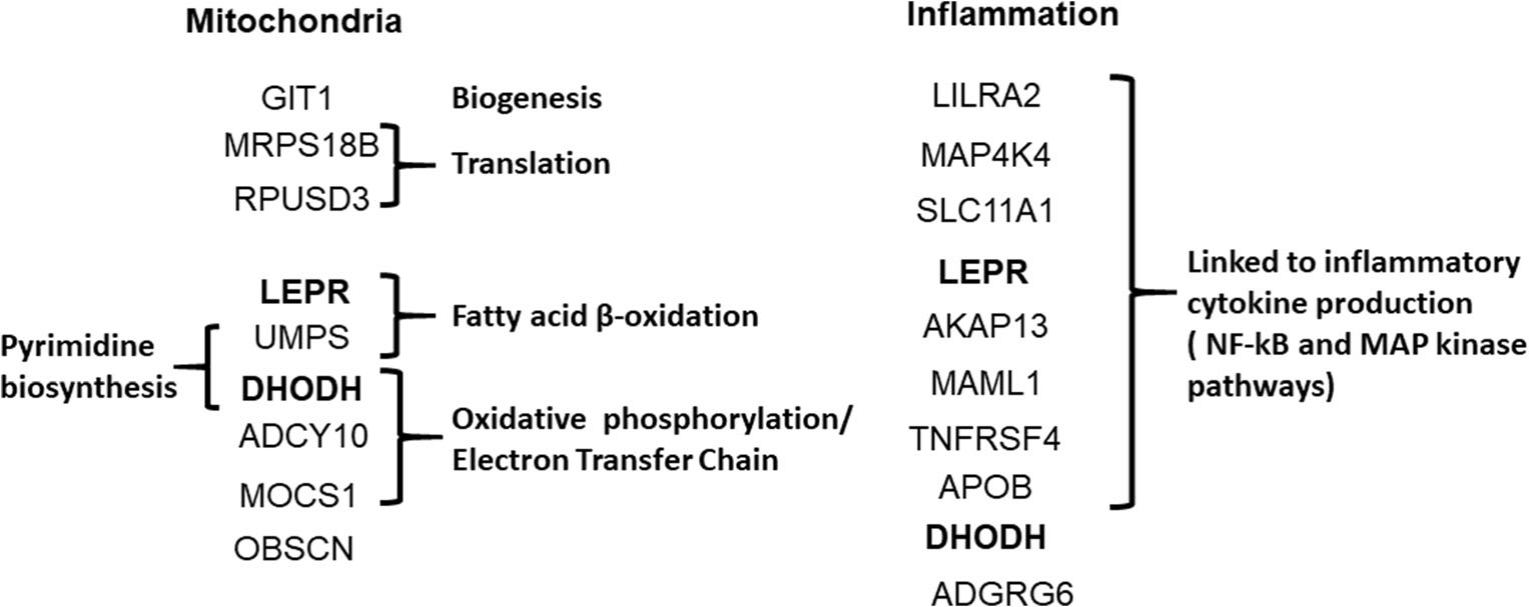
Features of the main mitochondrial and inflammation-related genes containing deleterious pathogenic variants associated to CCC

Whole exome sequencing disclosed that on average, each patient sample contained 41,780 gene variants. Among them, on average, 11,651 variants were located in coding (exonic or splicing) regions and nonsynonymous. We focused on variants characterized by a minor allele frequency < 1% in the databases (ESP6500, 1000G, and ExAC). Under a hypothesis of complete penetrance, for each given family, we selected nonsynonymous exonic or splicing variants shared by all family members with CCC but absent from ASY family members as well as by the unrelated ASY controls. For families 1 to 6, we found 39, 8, 108, 81, 9, and 76 variants fulfilling the above criteria, respectively, comprising a total of 321 CCC-specific nonsynonymous exonic rare variants. We performed a Reactome (reactome.org) pathways analysis of the 321 CCC-specific variants, prior to any prioritization. We found 8 enriched pathways with a significant false discovery ratio (FDR < 0.05). Pathways were based on a very limited number of genes (6/8 with one or two genes) and thus of limited relevance. At any event, the 8 pathways were related either to interferon signaling or to endosomal/antigen processing/presentation pathways (HLA molecules, BTK) (online Table 4**)**. After the application of our pathogenicity filter with multiple algorithms, we found 102 CCC-specific rare pathogenic variants. After filtering for evolutionary conservation, we found 87 variants. At this point, we prioritized the 87 gene variants in 9 pathobiological processes associated to CD. It highlighted 23 of our candidate genes (corresponding to 25 variants). From these variants, 88% (22/25, contained in 20 genes) were confirmed by Sanger sequencing (Table 2, online Fig. 1). All these 22 variants showed CADD scores above 15, consistent with pathogenicity. Indeed, for information, the CADD score of each variant is also shown in Table 2b. Two variants have a CADD score above 15, while the other 20 variants have a CADD score above 20. Markers with a CADD score over 20 are usually included in the top 1% deleterious variants in the genome. For the remaining 62 genes, we performed a second gene set enrichment analysis using Ingenuity Pathway Analysis and Reactome, but no significant pathways were identified. Each family had CCC-associated 1 to 7 pathogenic variants in 1 to 6 genes. Each of the 22 CCC-specific variants only appeared in a single nuclear family. However, for the APOB gene, two variants were detected in two different families. Figure 2 shows the number of genes containing associated variants in each pathophysiological process. Some genes may be common to several pathways. Table 2 shows the detailed information on the 22 gene variants (2A), pathogenicity and conservation scores (2B), participation in pathobiological processes relevant for inflammatory cardiomyopathy (2C), and their frequency in the databases (2D). The frequencies of our variants of interest in Latino reference subpopulations is described in online Table 6. We found a striking accumulation of CCC-associated variants in inflammation-related and mitochondrial genes (17 out of the 20 genes). All families carried at least one variant in mitochondrial or inflammation-associated genes; five families carried variants in mitochondrial genes and 5 in inflammation-related genes. A total of 10 pathogenic variants were found in 9 mitochondrial genes (ADCY10, DHODH, GIT1, MRPS18B, RPUSD3, LEPR, UMPS, MOCS1, and OBSCN). A total of 11 pathogenic variants were located in 10 inflammation-associated genes (ADGRG6, AKAP13, LEPR, LILRA2, MAML1, MAP4K4, SLC11A1, TNFRSF4, APOB, and DHODH) (Figs. 2 and 3, Table 2). This accumulation of genes was not an artifact of selection, since the number of prioritized variants in each pathway was not proportional to the total number of genes in the pathway/process. For instance, while we found 10 variants in 1532 mitochondrial genes, there were 4 variants in the 951 genes belonging to the fibrosis/extracellular matrix process.

**Table 2:** Description of pathogenic variants identified on the 6 nuclear families. A. Genetic data. B. pathogenicity and conservation. C. Participation in select pathobiological processes. D. Frequency of variants in different databases

A											
Family	Gene acronym	Chr	Start	End	Reference/mutated allele	Localization	Type of mutation	Nucleic acid change	Amino acid change	avsnp147	
1	LEPR	1	66,081,791	66,081,791	C/T	Exonic	non syn	exon14 2096C > T (NM_001198687)	T699M	rs34499590	
1	ADCY10	1	167,830,254	167,830,254	T/C	Exonic	non syn	exon12 1205A > G (NM_001167749)	Y402C	rs140663029	
1	MOCS1	6	39,877,666	39,877,666	G/A	Exonic	non syn	exon8 1015C > T (NM_005943)	R339W	rs148579886	
1	ADGRG6	6	142,724,940	142,724,940	G/A	Exonic; splicing	non syn	exon13 1873G > A (NM_001032394)	A625T	rs184235213	
1	AKAP13	15	86,124,694	86,124,694	T/C	Exonic	non syn	exon7 3395 T > C (NM_006738)	L1132S	rs745783128	
2	OBSCN	1	228,464,267	228,464,267	G/T	Exonic	non syn	exon22 6337G > T (NM_001098623)	G2113C	rs74623201	
3	APOB	2	21,247,996	21,247,996	C/A	Exonic; splicing	non syn	exon16 2245G > T (NM_000384)	D749Y	.	
3	MRPS18B	6	30,590,612	30,590,612	G/A	Exonic; splicing	non syn	exon5 358G > A (NM_014046)	V120M	rs116524936	
3	PKHD1	6	51,947,999	51,947,999	G/A	Exonic	non syn	exon3 107C > T (NM_138694)	T36M	rs137852944	
3	RNLS	10	90,122,344	90,122,344	C/T	Exonic	non syn	exon5 665G > A (NM_001031709)	R222H	rs191733133	
3	GIT1	17	27,901,773	27,901,773	C/T	Exonic	non syn	exon20 2233G > A (NM_014030)	A745T	.	
3	GIT1	17	27,910,559	27,910,559	C/T	Exonic	non syn	exon2 128G > A (NM_001085454)	R43H	.	
3	LILRA2	19	55,098,715	55,098,715	C/T	Exonic	non syn	exon6 1267C > T (NM_001290270)	R423C	rs149580797	
4	MAP4K4	2	102,440,480	102,440,480	A/G	Exonic	non syn	exon4 271A > G (NM_001242559)	K91E	.	
4	SLC11A1	2	219,257,728	219,257,728	C/T	Exonic	non syn	exon12 1189C > T (NM_000578)	R397C	rs74906275	
4	RPUSD3	3	9,880,802	9,880,802	C/T	Exonic	stopgain	exon8 806G > A (NM_001351738)	W269X	rs142984515	
4	UMPS	3	124,449,406	124,449,406	A/G	Exonic	non syn	exon1 88A > G (NM_000373)	S30G	rs17843776	
5	MAML1	5	179,192,418	179,192,418	G/A	Exonic	non syn	exon2 407G > A (NM_014757)	G136E	rs146382198	
5	DHODH	16	72,048,540	72,048,540	C/T	Exonic	non syn	exon3 403C > T (NM_001361)	R135C	rs201230446	
6	TNFRSF4	1	1,147,467	1,147,467	G/C	Exonic	non syn	exon5 489C > G (NM_003327)	D163E	.	
6	APOB	2	21,230,419	21,230,419	G/C	Exonic	non syn	exon26 9321C > G (NM_000384)	N3107K	rs72653101	
6	SERPINE2	2	224,866,427	224,866,427	A/G	Exonic	non syn	exon2 191 T > C (NM_001136528)	M64T	rs34078713	
B											
Family	Gene	Amino acid change	avsnp147	Polyphen2 HDIV prediction	Polyphen2 HVAR prediction	SIFT prediction	UMD prediction	CADD prediction score	Consurf conservation score		
1	LEPR	T699M	rs34499590	Damaging	Damaging	Damaging	Prob Patho	26.5	9		
1	ADCY10	Y402C	rs140663029	Damaging	Damaging	Damaging	Poly	22.2	6		
1	MOCS1	R339W	rs148579886	Damaging	Damaging	Damaging	Patho	33.0	8		
1	ADGRG6	A625T	rs184235213	Damaging	Damaging	Tolerate	Patho	26.0	9		
1	AKAP13	L1132S	rs745783128	Damaging	Prob Dam	Damaging	Prob Patho	15.6	7		
2	OBSCN	G2113C	rs74623201	Damaging	Damaging	Damaging	Prob Poly	25.0	9		
	APOB	D749Y	.	Damaging	Damaging	Damaging	Patho	28.5	7		
3	MRPS18B	V120M	rs116524936	Damaging	Prob Dam	Damaging	Prob Patho	29.4	3		
3	PKHD1	T36M	rs137852944	Damaging	Damaging	Damaging	Patho	30.0	9		
3	RNLS	R222H	rs191733133	Damaging	Damaging	Damaging	Prob Patho	33.0	7		
3	GIT1	A745T	.	Damaging	Damaging	Damaging	Patho	33.0	9		
3	GIT1	R43H	.	Damaging	Damaging	Damaging	Poly	35.0	9		
3	LILRA2	R423C	rs149580797	Damaging	Damaging	Damaging	Prob Poly	24.5	8		
4	MAP4K4	K91E	.	Damaging	Damaging	Damaging	Patho	24.4	3		
4	SLC11A1	R397C	rs74906275	Damaging	Damaging	Damaging	Poly	33.0	6		
4	RPUSD3	W269X	rs142984515	Damaging	Damaging	Damaging	Prob Poly	27.9	4		
4	UMPS	S30G	rs17843776	Prob Dam	Prob Dam	Damaging	Poly	23.4	9		
5	MAML1	G136E	rs146382198	Damaging	Prob Dam	Tolerate	Patho	15.3	3		
5	DHODH	R135C	rs201230446	Damaging	Damaging	Damaging	Patho	34.0	9		
6	TNFRSF4	D163E	.	Damaging	Damaging	Damaging	Poly	22.3	9		
6	APOB	N3107K	rs72653101	Damaging	Damaging	Damaging	Prob Poly	22.9	7		
6	SERPINE2	M64T	rs34078713	Prob Dam	Prob Dam	Damaging	Prob Patho	26.7	8		
C											
Family	Gene	Amino acid change	avsnp147	Inflammation	Mitochondrial genes	IFNγ modulated genes/Th1 response	Hypertrophy	Muscle contraction and contractility	Fibrosis extracellular matrix	Oxidative stress/antioxidant response	Familial CMP genes
1	LEPR	T699M	rs34499590	X	X		X		X		
1	ADCY10	Y402C	rs140663029		X						
1	MOCS1	R339W	rs148579886		X						
1	ADGRG6	A625T	rs184235213	X							
1	AKAP13	L1132S	rs745783128	X			X		X		
2	OBSCN	G2113C	rs74623201		X						X
3	APOB	D749Y	.	X				X			
3	MRPS18B	V120M	rs116524936		X						
3	PKHD1	T36M	rs137852944						X		
3	RNLS	R222H	rs191733133				X	X			
3	GIT1	A745T	.		X	X	X				
3	GIT1	R43H	.		X	X	X				
3	LILRA2	R423C	rs149580797	X							
4	MAP4K4	K91E	.	X		X					
4	SLC11A1	R397C	rs74906275	X		X				X	
4	RPUSD3	W269X	rs142984515		X						
4	UMPS	S30G	rs17843776		X	X					
5	MAML1	G136E	rs146382198	X							
5	DHODH	R135C	rs201230446	X	X					X	
6	TNFRSF4	D163E	.	X							
6	APOB	N3107K	rs72653101	X				X			
6	SERPINE2	M64T	rs34078713						X		
D											
Family	Gene	Amino acid change	avsnp147	Polymorphism frequency in the ESP6500 database	Polymorphism frequency in the 1000G database	Polymorphism frequency in theExAC database	Polymorphism frequency in the Brazilian genomic variant cohort (ABraOM)				
1	LEPR	T699M	rs34499590	1.28%	0.98%	0.32%	0.82%				
1	ADCY10	Y402C	rs140663029	0.04%	0.080%	0.02%	0.16%				
1	MOCS1	R339W	rs148579886	0.02%	0.040%	0.04%	NA				
1	ADGRG6	A625T	rs184235213	0.24%	0.26%	0.07%	0.08%				
1	AKAP13	L1132S	rs745783128	NA	NA	0.0008%	NA				
2	OBSCN	G2113C	rs74623201	0.59%	0.50%	0.16%	0.08%				
3	APOB	D749Y	.	NA	NA	NA	NA				
3	MRPS18B	V120M	rs116524936	NA	0.02%	0.002%	NA				
3	PKHD1	T36M	rs137852944	0.03%	0.02%	0.05%	NA				
3	RNLS	R222H	rs191733133	NA	0.02%	0.002%	NA				
3	GIT1	A745T	.	NA	NA	NA	NA				
3	GIT1	R43H	.	NA	NA	NA	NA				
3	LILRA2	R423C	rs149580797	0.21%	0.30%	0.09%	0.33%				
4	MAP4K4	K91E	.	NA	NA	NA	NA				
4	SLC11A1	R397C	rs74906275	0.04%	0.04%	0.03%	NA				
4	RPUSD3	W269X	rs142984515	0.008%	NA	0.007%	NA				
4	UMPS	S30G	rs17843776	0.02%	0.36%	0.38%	NA				
5	MAML1	G136E	rs146382198	0.008%	0.08%	0.07%	0.49%				
5	DHODH	R135C	rs201230446	0.04%	0.04%	0.04%	0.08%				
6	TNFRSF4	D163E	.	NA	NA	NA	NA				
6	APOB	N3107K	rs72653101	NA	NA	0.0008%	NA				
6	SERPINE2	M64T	rs34078713	0.78%	0.30%	0.75%	1.15%				

*Prob*, probable or probably; *Patho*, pathogenic; Poly = poly = polymorphism; *Dam*, damaging

*CMP*, cardiomyopathy

We excluded synonymous, non-exonic polymorphisms, keeping polymorphisms with a minor allele frequency (MAF) of <1% in at least one public databases (ESP6500; NHLBI GO Exome Sequencing Project (EVS, ESP6500SI-V2 release on http://evs.gs.washington.edu/EVS/); 1000 Genomes (April 2014 data release on http://browser.1000genomes.org); and Exome Aggregation Consortium (ExAC, January 2015 Version 0.3 data release onhttp://exac.broadinstitute.org)) using the VARAFT filtering and annotation tool (https://varaft.eu) on vcf files. Variant calls with Variant quality (QUAL) ≤60, depth of coverage (DP) <20, and mapping quality (MQ) ≤40 were filtered out. We also assessed an available Brazilian exome database of 609 elderly individuals from the city of Sao Paulo [18]. Only exonic nonsynonymous damaging variants were kept for downstream genetic analyses. We searched for variants, independently, in each family, under an autosomal dominant or under autosomal recessive models. In order to identify gene variants associated with CCC in each family, we selected variants that were shared by all CCC patients and absent in any and all IF patients in a given nuclear family, as well as in 14 unrelated IF controls. We assessed the pathogenic potential of missense variants using 4 algorithms embedded in VarAft: SIFT (https://sift.bii.a-star.edu.sg/), Polyphen 2 HumDiv and Polyphen 2 HumVar (http://genetics.bwh.harvard.edu/pph2/index.shtml), and UMD-Predictor (http://umd-predictor.eu/). Only rare variants tagged as pathogenic (or damaging) or possibly pathogenic (or probably damaging) in at least three databases and pathogenic/damaging in at least one algorithm were retained for downstream genetic analyses. We validated the abovementioned pathogenicity filter with the Combined Annotation-Dependent Depletion (CADD) tool (http://cadd.gs.washington.edu/score). Evolutionary conservation in the position was determined with the Consurf server (https://consurf.tau.ac.il)

Conversely, 3 variants were found in the 178 genes belonging to the contraction/contractility process. Figure 3 shows the participation of the variant genes in different mitochondrial and inflammation-related pathways. Eight out of 9 mitochondrial genes with CCC-specific variants are involved directly or indirectly with energy generation, in processes including mitochondrial biogenesis, mitochondrial DNA-encoded gene translation, fatty acid oxidation, and oxidative phosphorylation/electron transfer chain; interestingly, two genes are involved in pyrimidine biosynthesis. Of note, 8 out of 10 inflammation-related genes are involved in proinflammatory cytokine production via activation of NF-kB and MAP kinase pathways. Two mitochondrial genes (DHODH and LEPR) are also involved in inflammation (Table 2, Fig. 3 in bold). On the whole, 5 of the inflammation-related genes and 5 of the mitochondrial genes also played roles in other cardiomyopathy-related pathobiological processes. The RPUSD3 gene, involved in the assembly of the mitochondrial ribosome, displayed a stopgain variant at exon 8 in family 4, creating a truncated version lacking 24% of its C-terminal sequence.

The GIT1 gene showed two variants in family 3 that segregated together, suggesting compound heterozygosity. Only 3 variants occurred in genes that were not mitochondrial and/or inflammation-related: PKHD1 and SERPINE2, which belonged to the ECM/fibrosis process, and RNLS, belonging to the hypertrophy and contraction processes. Only one variant gene, OBSCN, had previously been described in genetic/familial cardiomyopathy. No variants were detected in arrhythmia/ion channel–related genes. Patients carrying heterozygous gene variants had a normal childhood and reported no debilitating disease before developing CCC as adults, and we inferred that the variants by themselves alone were not able to induce childhood-onset mitochondriopathy. Detailed information on the variants is described in Table 2.

We have performed Sanger sequencing in two healthy, eutrophic, seronegative siblings from family 5 searching for the CCC-associated variants in genes DHODH and MAML1. One of them (subject 6137) carried the heterozygote variant DHODH C/T (R135C) shared by the CCC family members, while the other (subject 6132) carried the “wild-type” homozygous DHODH n.403 C/C (R135). Regarding MAML1, the second variant gene in Family 5, both 6132 and 6137 carried the “wild-type” homozygous MAML1 n. 407 G/G (G136).

**Fig. 4:**
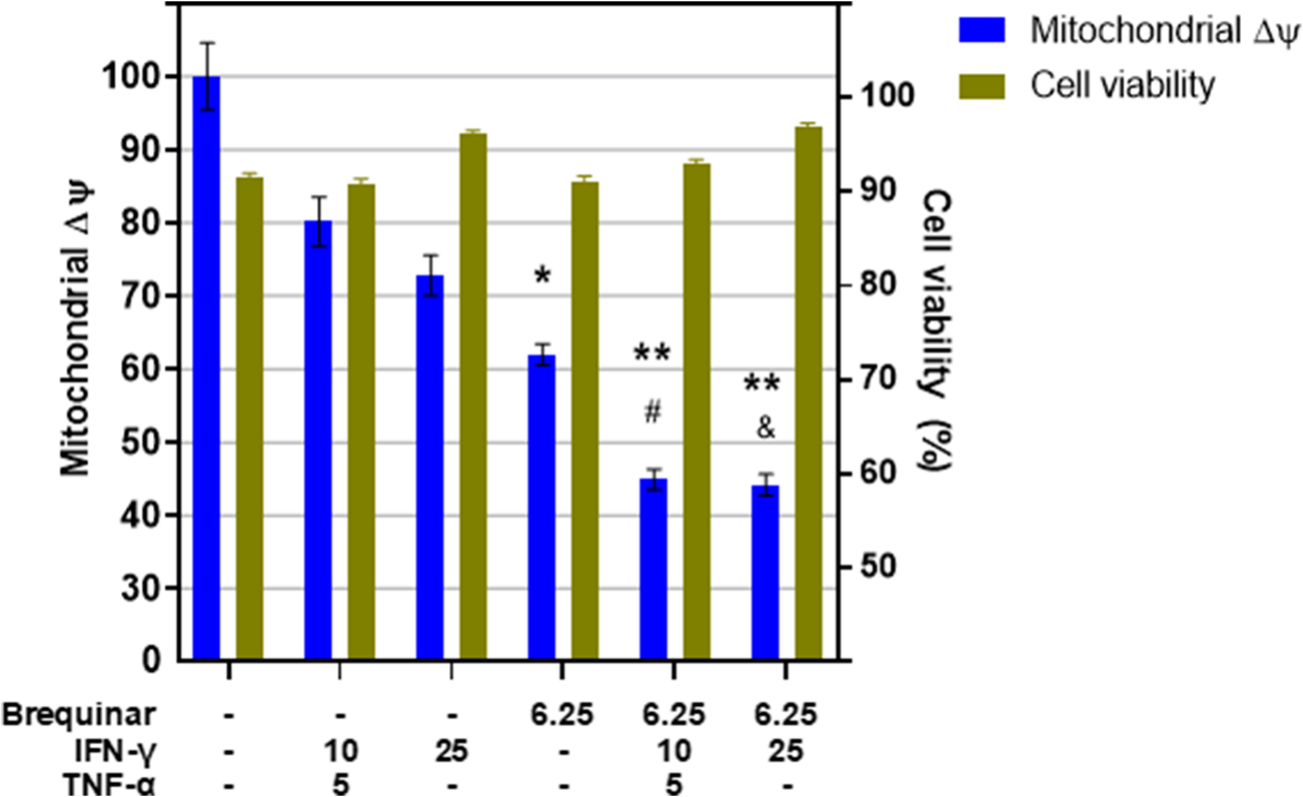
IFN-γ and brequinar treatment significantly reduced mitochondrial membrane potential. AC16 human cardiomyocyte cell line was stimulated with or without IFN-γ and with or without brequinar for 48 h. Total mitochondria were stained with MitoTracker green, and mitochondrial membrane potential (MMP) was evaluated using the TMRE dye that accumulates in fully polarized, but not in depolarized mitochondria. We measured TMRE fluorescence on MitoTracker green-labeled mitochondria. Cell viability was calculated as the ratio of the amount of live cells (propidium iodide-negative) and total cells (propidium iodide-negative plus propidium iodide-positive cells) × 100. * *p* < 0.05, ** *p* < 0.01, *** *p* < 0.001, and **** *p* < 0.0001

Since both IFN-γ and loss-of-function mitochondrial mutations cause mitochondrial dysfunction, we studied the effect of IFN-γ on cardiomyocytes made deficient in mitochondrial enzyme dihydroorotate dehydrogenase (DHODH) activity with the inhibitor Brequinar. This enzyme is important for the electron transport chain and showed a loss-of-function mutation (DHODH R135C) linked with CCC in one of the studied families. We found that both IFN-γ and brequinar treatment significantly reduced mitochondrial membrane potential (ΔψM) as expressed by mitochondrial TMRE fluorescence on the AC16 human cardiomyocyte cell line and that cells treated simultaneously with IFN-γ and brequinar showed an even larger decrease on ΔψM (Fig. 4).

## Discussion

In this study of whole exome sequencing of six nuclear families with multiple cases of CD, we found 22 CCC-associated rare heterozygous nonsynonymous high-impact pathogenic variants in 20 genes belonging to pathways relevant to inflammatory cardiomyopathy. Only individuals that were both seropositive and carriers of the heterozygous pathogenic variants developed CCC, but not seropositive patients carrying the wild-type sequences, nor seronegative siblings carrying the pathogenic variant. A striking accumulation of CCC-specific variants (86%) occurred in mitochondrial or inflammation-related genes, and all studied families displayed at least one CCC-associated variant gene belonging to these pathways. The finding that only a single family carried a variant in a gene previously associated with familial cardiomyopathy indicates the genetic landscape, and pathogenesis of CCC is distinct from that of familial cardiomyopathy. Importantly, we also found that IFN-γ potentiated mitochondrial dysfunction caused by an inhibitor of DHODH, a mitochondrial enzyme bearing a loss-of-function mutation (DHODH R135C) linked with CCC in one of our families, indicating synergy in the induction of mitochondrial dysfunction. To our knowledge, this is the first report that rare heterozygous pathogenic variants in mitochondrial and inflammation-related genes are linked to the development of complex multifactorial inflammatory cardiomyopathy.

Among the 9 mitochondrial genes showing CCC-specific pathogenic variants, 8 are involved in processes leading to mitochondrial ATP production (biogenesis, translation, fatty acid oxidation-FAOx, and the electron transfer chain/oxidative phosphorylation (OXPHOS)). The R135C variant observed in the DHODH gene was the only variant known to lead to complete loss-of-function and mitochondrial dysfunction [27]. Genetic truncation or expression of dominant-negative variants of DHODH, GIT1, RPUSD3, ADCY10, and MOCS1 in cells was shown to cause mitochondrial dysfunction, including reduced mitochondrial membrane potential, respiratory chain/oxidative phosphorylation (OXPHOS) activity, and ATP production [28–30]. Interestingly, patients carrying mutations or animals genetically deficient in 6 genes (DHODH, UMPS, MRPS18B, GIT1, OBSCN, and LEPR) developed cardiac phenotypes [27, 30–34]. Parents of patients with Miller syndrome (homozygous DHODH loss-of-function R135C or other variants; 70% with congenital cardiac abnormalities) carry one copy of the defective gene as members of family 5 but are nevertheless healthy [27]. Animals genetically deficient in MRPS18B and GIT1 showed evidence of mitochondrial dysfunction, altered mitochondrial morphology, and reduced myocardial contractility [35]. Details about the function of affected genes are shown in online Table 5.

Genetic disorders involving mitochondrial genes are the most common congenital genetic syndromes. Mitochondriopathies are caused by homozygous pathogenic rare variants in nuclear-encoded mitochondrial genes or in mitochondrial DNA. Homozygous pathogenic variants of more than 250 of the ~1500 nuclear-encoded mitochondrial genes were identified as causally related to mitochondrial disorders [35]. Each leads to drastic mitochondrial dysfunction and energetic and functional impairment in tissues of high energy demand, causing syndromes affecting single organs or organ combinations that usually present in early childhood; each clinical syndrome is associated with variants in specific mitochondrial genes [35], and clinical penetrance is variable. The diverse clinical presentation of mitochodriopathies is related to genetic heterogeneity and the central role of mitochondria in multiple processes, including energy generation, antioxidant defenses, Ca++ homeostasis, intracellular signaling, macromolecule and nucleotide biosynthesis, reprogramming nuclear gene expression, and cell death. Interestingly, up to 30% of mitochondriopathy patients develop cardiomyopathy, heart conduction defects, ventricular arrhythmia or sudden cardiac death, and autonomic nervous system imbalance [36], while up to 15% develop gastrointestinal motility disorders including achalasia/megaesophagus and megacolon [37]. The striking similarity between the clinical presentation and proportion of cardiac and digestive disorders in mitochondriopathies and the clinical spectrum of CD [3] suggested the pathogenesis of CCC may be dependent on mitochondrial dysfunction. Indeed, CCC myocardium displays signs of reduced mitochondrial activity and energy production. Decreased mitochondrial rRNA [10], rDNA [38], and in vivo ATP production [18] were observed in the CCC myocardium. Myocardial levels and activity of mitochondrial energy metabolism enzymes ATP synthase and creatine kinase are even lower than in CCC than other cardiomyopathies [17], which might contribute to the worse prognosis of CCC. This is in line with Nisha Garg’s group findings implicating myocardial mitochondrial dysfunction and oxidative stress in the pathogenesis of murine models of CCC (reviewed in [39]). IFN-γ and brequinar treatment significantly reduced mitochondrial membrane potential (ΔψM) on the AC16 human cardiomyocyte cell line. Significantly, cells treated simultaneously with IFN-γ, and brequinar showed an even larger decrease on ΔψM. This result is consistent with an increased susceptibility of cells with reduced mitochondrial enzyme activity to IFN-γ. Even though the identified genes play important roles in mitochondrial energy generation and physiology, the heterozygous mitochondrial variants did not cause childhood signs of altered development typical of mitochondriopathies and were only associated with progression to cardiac disease in *T. cruzi*-seropositive patients, whose age group was typical from CCC patients in general. Taken together, our results suggest that *T. cruzi*-induced IFN-γ is necessary to cause clinically significant disease in patients with heterozygous variants. We propose that long-term exposure to high levels IFN-γ associated with CD – particularly high in CCC myocardium [40] – is the additional mitochondrial insult suffered by carrier of heterozygous mitochondrial variants that leads them to mitochondrial dysfunction. IFN-γ has multiple deleterious effects on cardiomyocyte mitochondria. It induces TNF-alpha and potentiates TNF-alpha-mediated NF-kB signaling, leading to NOS2 production of NO [41], which in the presence of IFN-γ-induced reactive oxygen species turns into peroxynitrite [21], and ensuing mitochondrial fragmentation and reduction of mitochondrial membrane potential, lipid beta-oxidation [14], and ATP generation [22], leading to cardiomyocyte dysfunction and apoptosis [15]. Variants in the inflammation-related genes may also lead to a mitochondrial dysfunction phenotype. Gain-of-function variants of genes linked to NF-kB or MAP kinase signaling could lead to increased proinflammatory cytokine production, which might lead to further mitochondrial dysfunction in cardiomyocytes [22], and this mitochondrial damage can be further fueled by IFN-γ [42]. It is thus possible that the common final pathogenic mechanism for mitochondrial and inflammation-associated gene variants might involve enhancement of IFN-γ-induced cardiac mitochondrial damage. As mitochondrial damage and release of damage-associated molecular patterns can itself induce the production of proinflammatory cytokines, a self-perpetuating positive feedback loop between inflammation and mitochondrial damage [43] may contribute to the chronicity of inflammation in CCC heart tissue.

Our study has limitations. CCC patients included in the pedigrees mostly have major diagnostic ECG changes without left ventricular dysfunction. However, follow-up studies show that asymptomatic chagasic individuals with ECG abnormalities typical of CCC show higher total and cardiac mortality in relation to chagasic individuals with normal ECG; up to 50% of deaths in this groups are due to SCD [6, 8, 44–46]. Similarly, conduction defects and arrhythmias can be the presenting symptoms of genetic mitochondriopathies [47]. Even though CCC has a progressive character and inflammatory mediators and mitochondrial dysfunction are key participants of arrhythmia [48] and heart failure [43], we can only hypothesize that the genetic variants identified in our study may play a direct role in the inflammation [10, 11] and mitochondrial dysfunction [17, 38] observed in end-stage CCC.

## Conclusion

Results indicate that the genetic contribution to CCC is polygenic and driven by several rare variants in genes that differ between families but are related to mitochondria and inflammation. Results imply that mitochondrial dysfunction and inflammation, key processes in the pathophysiology of CCC, are at least in part genetically determined. To our knowledge, this is the first report that rare variants in mitochondrial and inflammation-related genes are linked to complex multifactorial cardiomyopathy. Our results also support the notion of a two-hit mechanism where IFN-γ and proinflammatory cytokines induced by chronic infection trigger mitochondrial dysfunction and clinical disease in carriers of heterozygous mitochondrial gene variants. Indeed, modulation of mitochondrial damage induced by IFN-γ and other cytokines could perhaps be a suitable therapeutic target in CCC. Treatment with mitochondria-protective agents such as antioxidants or agonists of sirtuin-1 and AMP-activated protein kinase (AMPK) was found to attenuate or even reverse cardiac damage in mouse models of CCC, by reducing NF-kB activation and the intensity of chronic myocarditis (reviewed in [38]). To conclude, it is possible that a similar two-hit mechanism, whereby genetic variants may increase mitochondrial susceptibility to inflammatory cytokine-induced dysfunction, may be relevant for the pathogenesis of other inflammatory cardiomyopathies and degenerative diseases associated with mitochondrial dysfunction.

## Supplementary Information

Online Fig. 1 Confirmation of the pathogenic variants by Sanger sequencing (PDF 2238 kb)

ESM 2 (DOCX 12 kb)

ESM 3 (DOCX 16 kb)

ESM 4 (DOCX 37 kb)

ESM 5 (DOCX 13 kb)

ESM 6 (DOCX 23 kb)

ESM 7 (DOCX 13 kb)

## Abbreviations

*T.* cruzi: *Trypanosoma cruzi*
CCC: Chronic Chagas cardiomyopathy
CD: Chagas disease
ASY: Asymptomatic form
DCM: Dilated cardiomyopathy
ECG: Electrocardiography
IFN: Interferon
TNF: Tumor necrosis factor
NO: Nitrite oxide
ATP: Adenosine triphosphate
AVM: Acute viral myocarditis
WES: Whole exome sequencing
SCD: Sudden cardiac death
HF: Heart failure
LEPR: Leptin receptor
ADCY10: Adenylate cyclase 10
MOCS1: Molybdenum cofactor synthesis 1
ADGRG6: Adhesion G protein-coupled receptor G6
AKAP13: AKinase-anchoring protein 13
OBSCN: Obscurin
APOB: Apolipoprotein B
MRPS18B: Mitochondrial ribosomal protein S18B
PKHD1: Polycystic kidney and hepatic disease 1
RNLS: Renalase, FAD-dependent amine oxidase
GIT1: G protein-coupled receptor kinase interactor 1
LILRA2: Leukocyte immunoglobulin–like receptor A2
MAP 4 K4: Mitogen-activated protein kinase kinase kinase kinase 4
SLC11A1: Solute carrier family 11 member 1
RPUSD3: RNA pseudouridine synthase D3
UMPS: Uridine monophosphate synthetase
MAML1: Mastermind like transcriptional coactivator 1
DHODH: Dihydroorotate dehydrogenase
TNFRSF4: TNF receptor superfamily member 1
SERPINE2: Serpin family E member 2
