# Supplementary material for: Rare Pathogenic Variants in Mitochondrial and Inflammation-Associated Genes May Lead to Inflammatory Cardiomyopathy in Chagas Disease
Source: J Clin Immunol. 2021 Mar 3;41(5):1048–63. doi: 10.1007/s10875-021-01000-y (PMC8249271; doi:10.1007/s10875-021-01000-y)
Supplement: Supplementary file 1 — Confirmation of the pathogenic variants by Sanger sequencing (PDF 2238 kb) [file 10875_2021_1000_MOESM1_ESM.pdf]

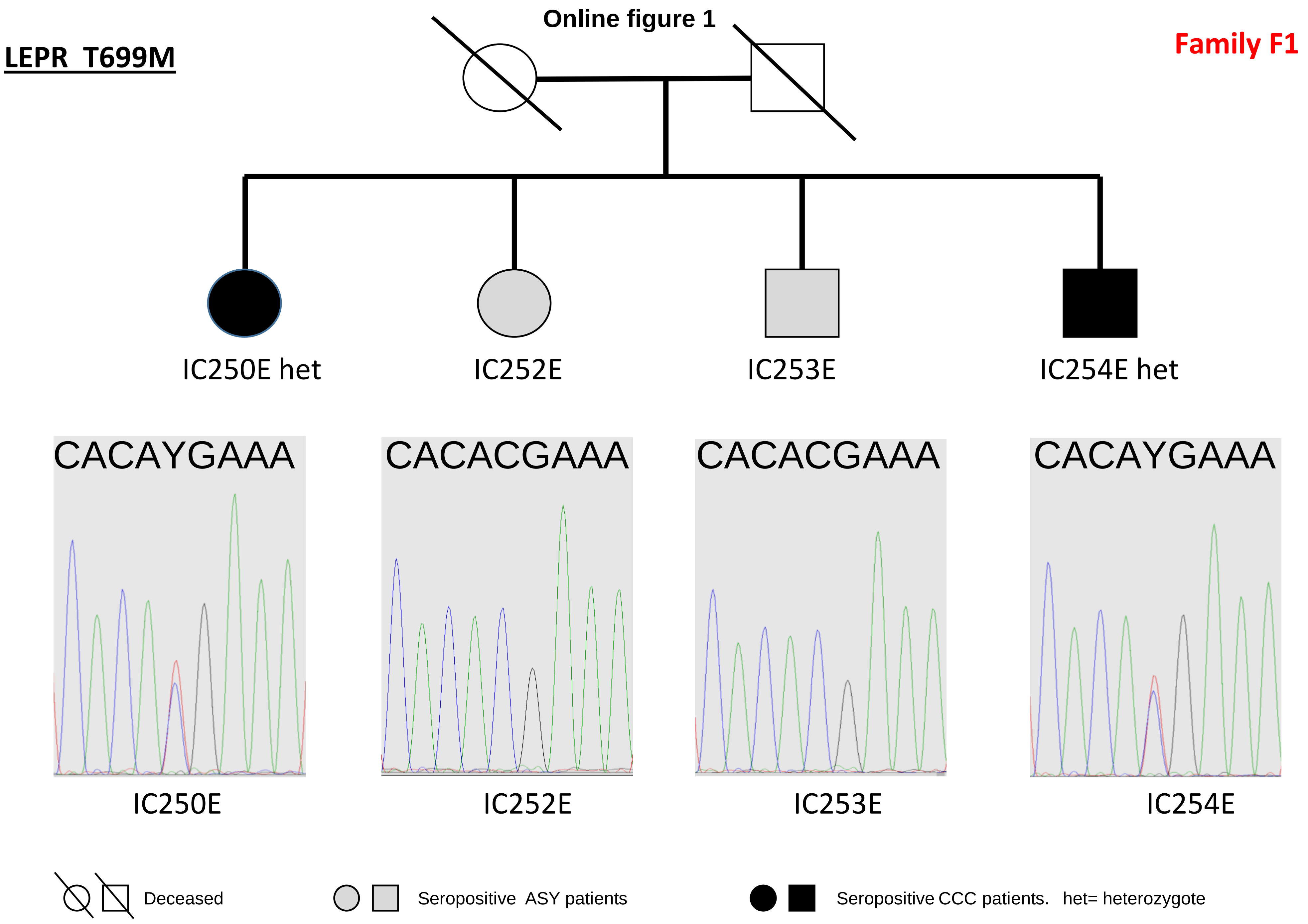
Online figure 1
LEPR T699M
Family F1
IC250E het
IC252E
IC253E
IC254E het
CACAYGAAA
CACACGAAA
CACACGAAA
CACAYGAAA
IC250E
IC252E
IC253E
IC254E
Deceased
Seropositive ASY patients
Seropositive CCC patients. het= heterozygote

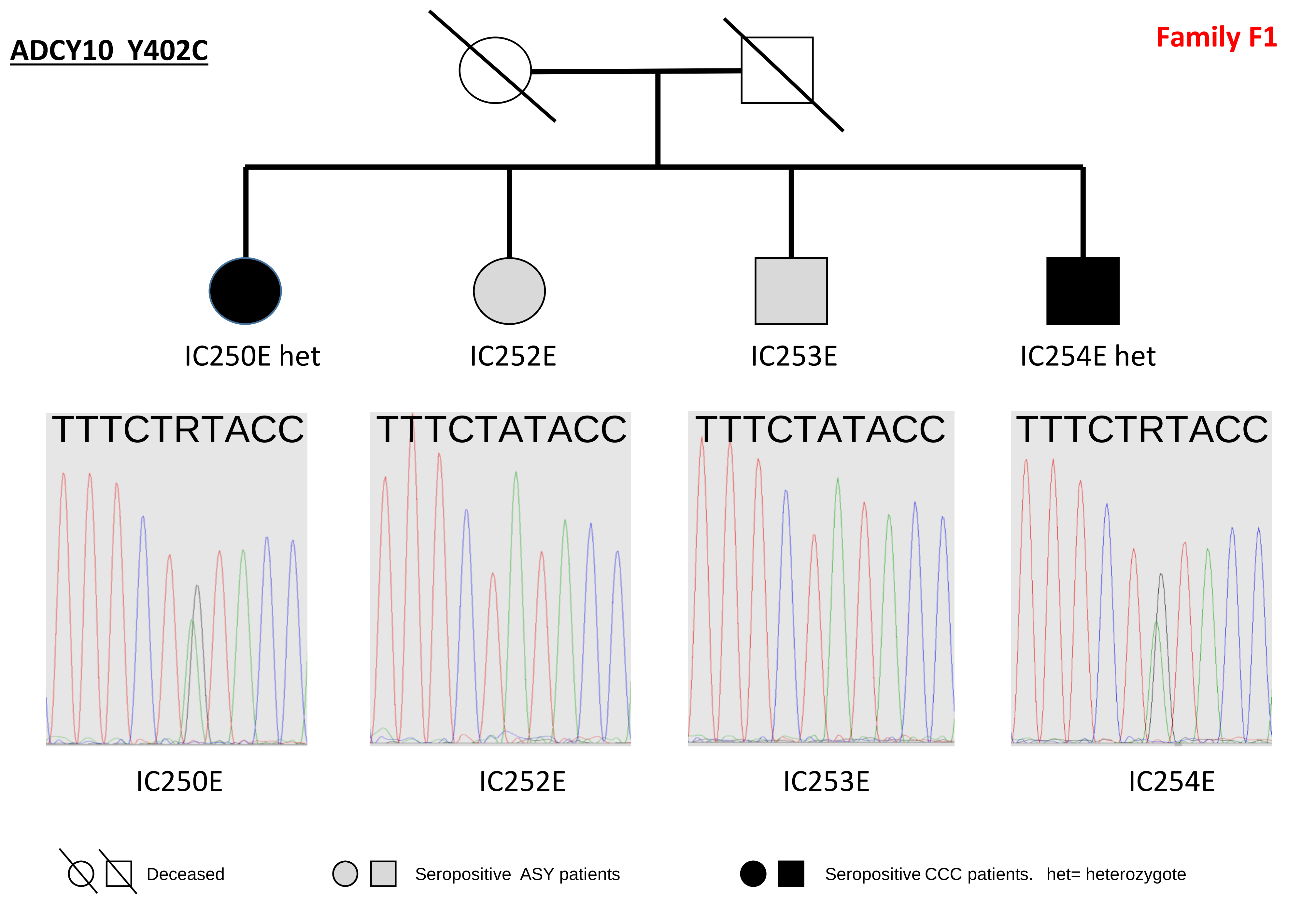
ADCY10 Y402C
Family F1
IC250E het
IC252E
IC253E
IC254E het
TTTCTRTACC
TTTCTATACC
TTTCTATACC
TTTCTRTACC
IC250E
IC252E
IC253E
IC254E
Deceased
Seropositive ASY patients
Seropositive CCC patients. het= heterozygote

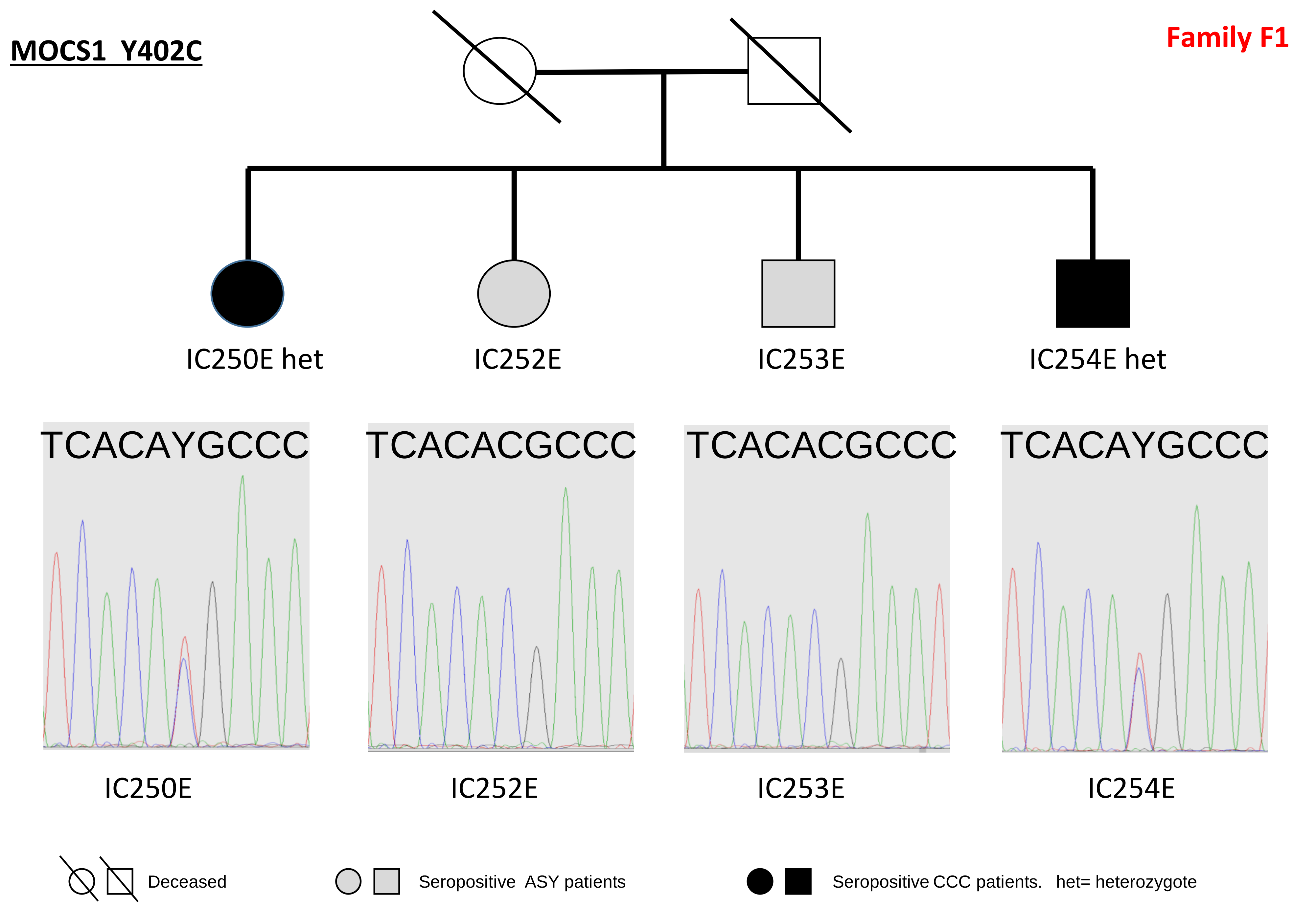
MOCS1 Y402C
Family F1
IC250E het
IC252E
IC253E
IC254E het
TCACAYGCCC
TCACACGCCC
TCACACGCCC
TCACAYGCCC
IC250E
IC252E
IC253E
IC254E
Deceased
Seropositive ASY patients
Seropositive CCC patients. het= heterozygote

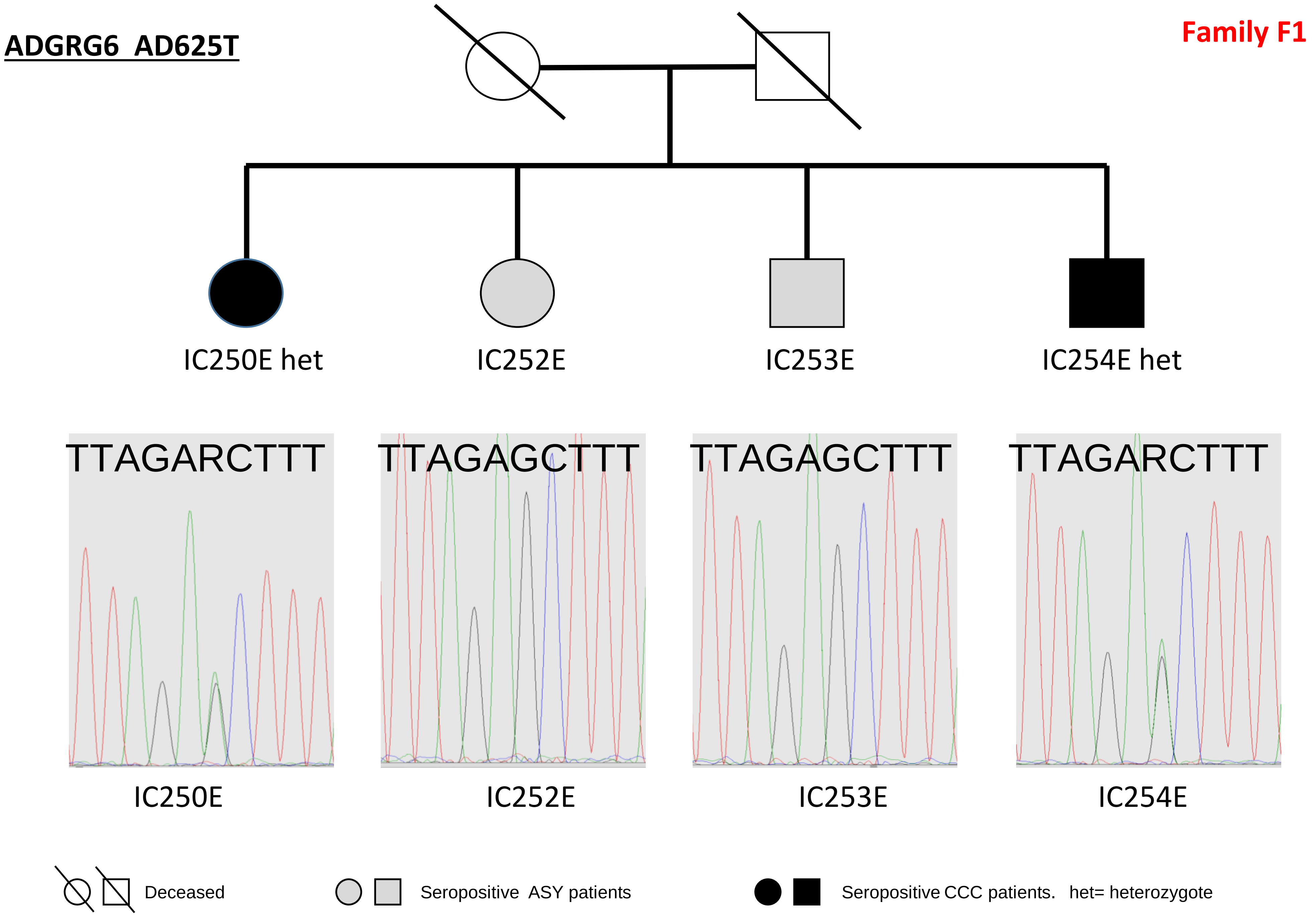

ADGRG6 AD625T
Family F1
IC250E het
IC252E
IC253E
IC254E het
TTAGARCTTT
TTAGAGCTTT
TTAGAGCTTT
TTAGARCTTT
IC250E
IC252E
IC253E
IC254E
Deceased
Seropositive ASY patients
Seropositive CCC patients. het= heterozygote

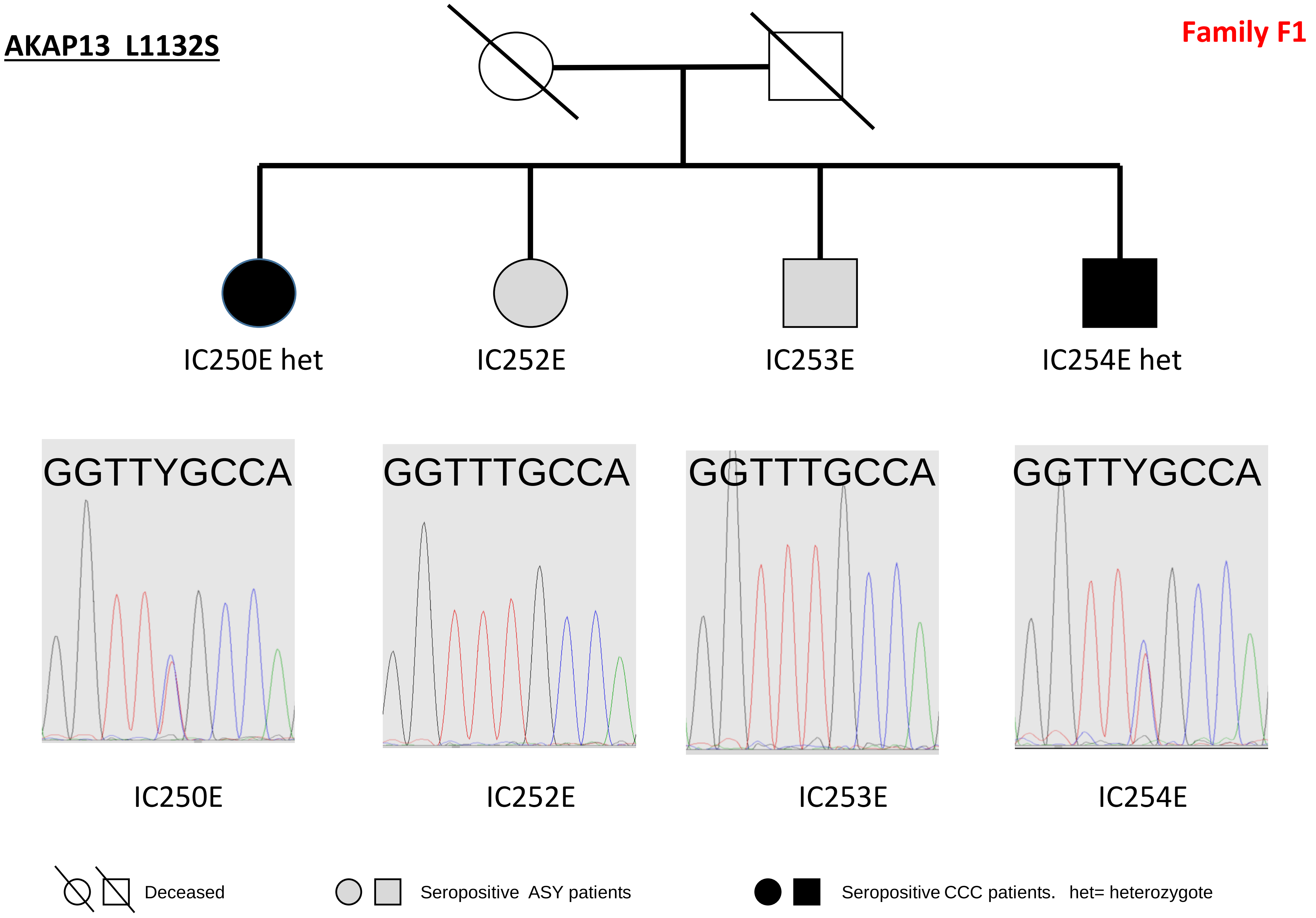
AKAP13 L1132S
Family F1
IC250E het
IC252E
IC253E
IC254E het
GGTTYGCCA
GGTTTGCCA
GGTTTGCCA
GGTTYGCCA
IC250E
IC252E
IC253E
IC254E
Deceased
Seropositive ASY patients
Seropositive CCC patients. het= heterozygote

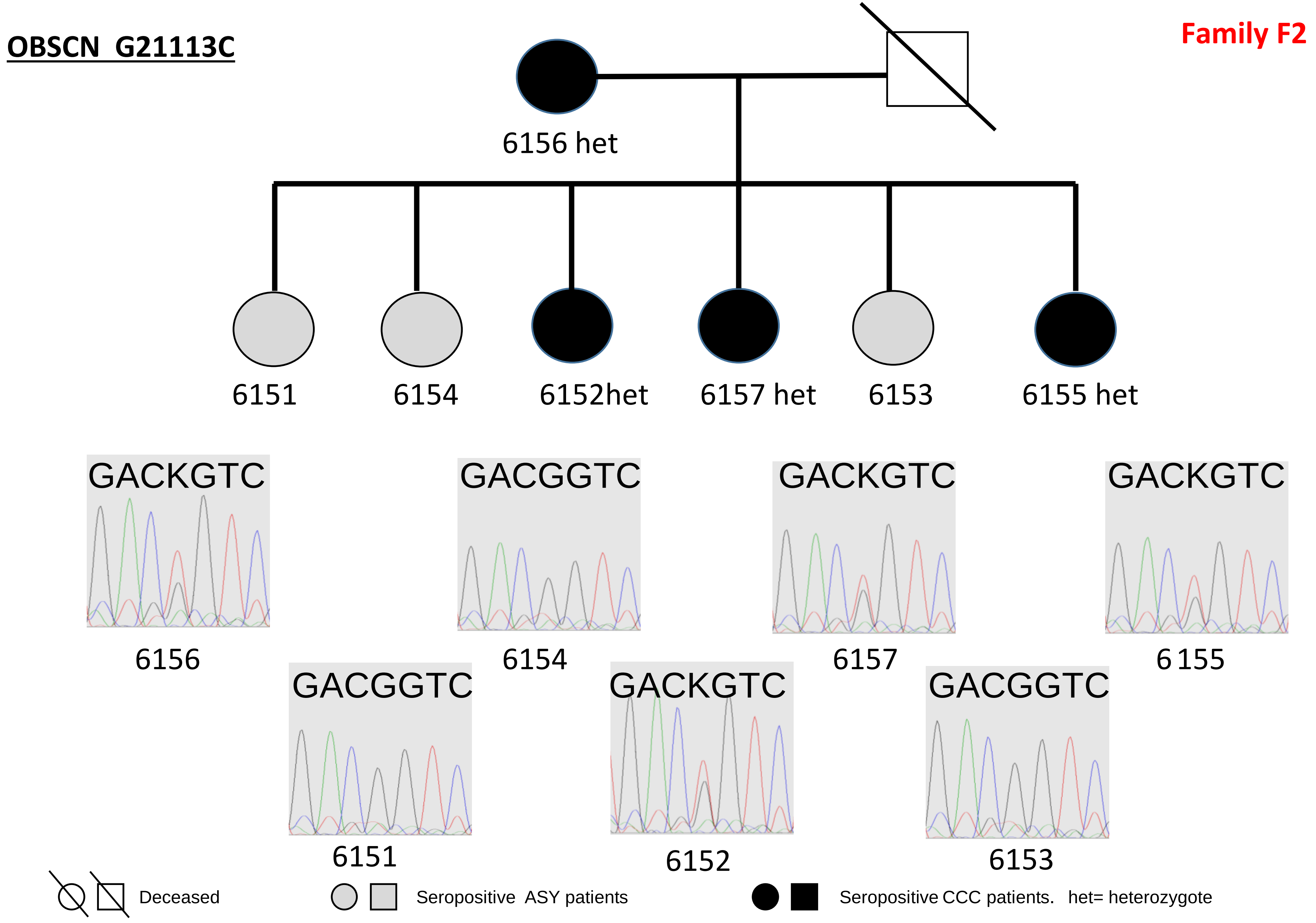

OBSCN G21113C
Family F2
6156 het
6151
6154
6152het
6157 het
6153
6155 het
GACKGTC
6156
GACGGTC
6154
GACKGTC
6157
GACKGTC
6 155
GACGGTC
6151
GACKGTC
6152
GACGGTC
6153
Deceased
Seropositive ASY patients
Seropositive CCC patients. het= heterozygote

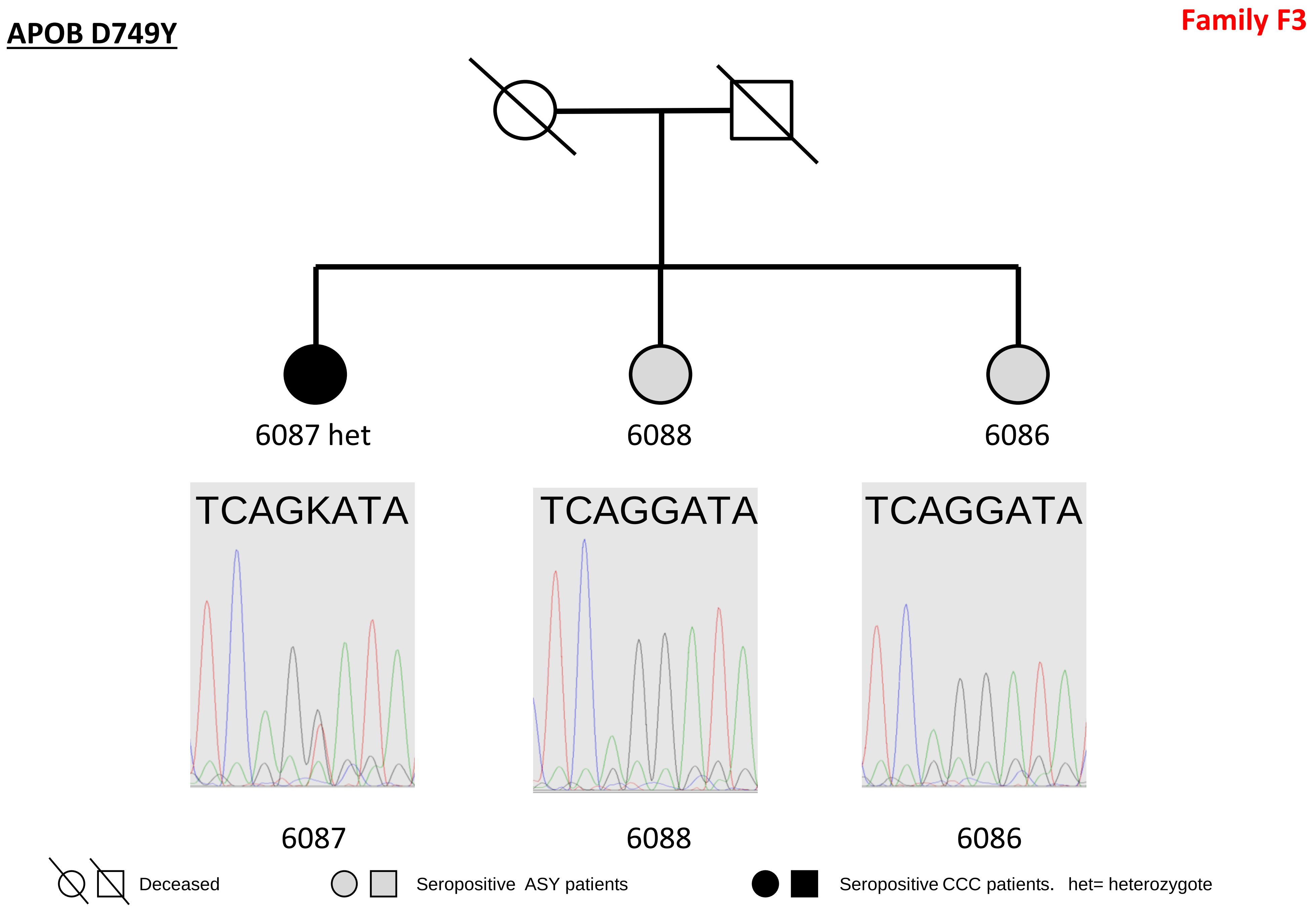

APOB D749Y
Family F3
6087 het
6088
6086
TCAGKATA
TCAGGATA
TCAGGATA
6087
6088
6086
Deceased
Seropositive ASY patients
Seropositive CCC patients. het= heterozygote

MRPS18B V120M

Family F3

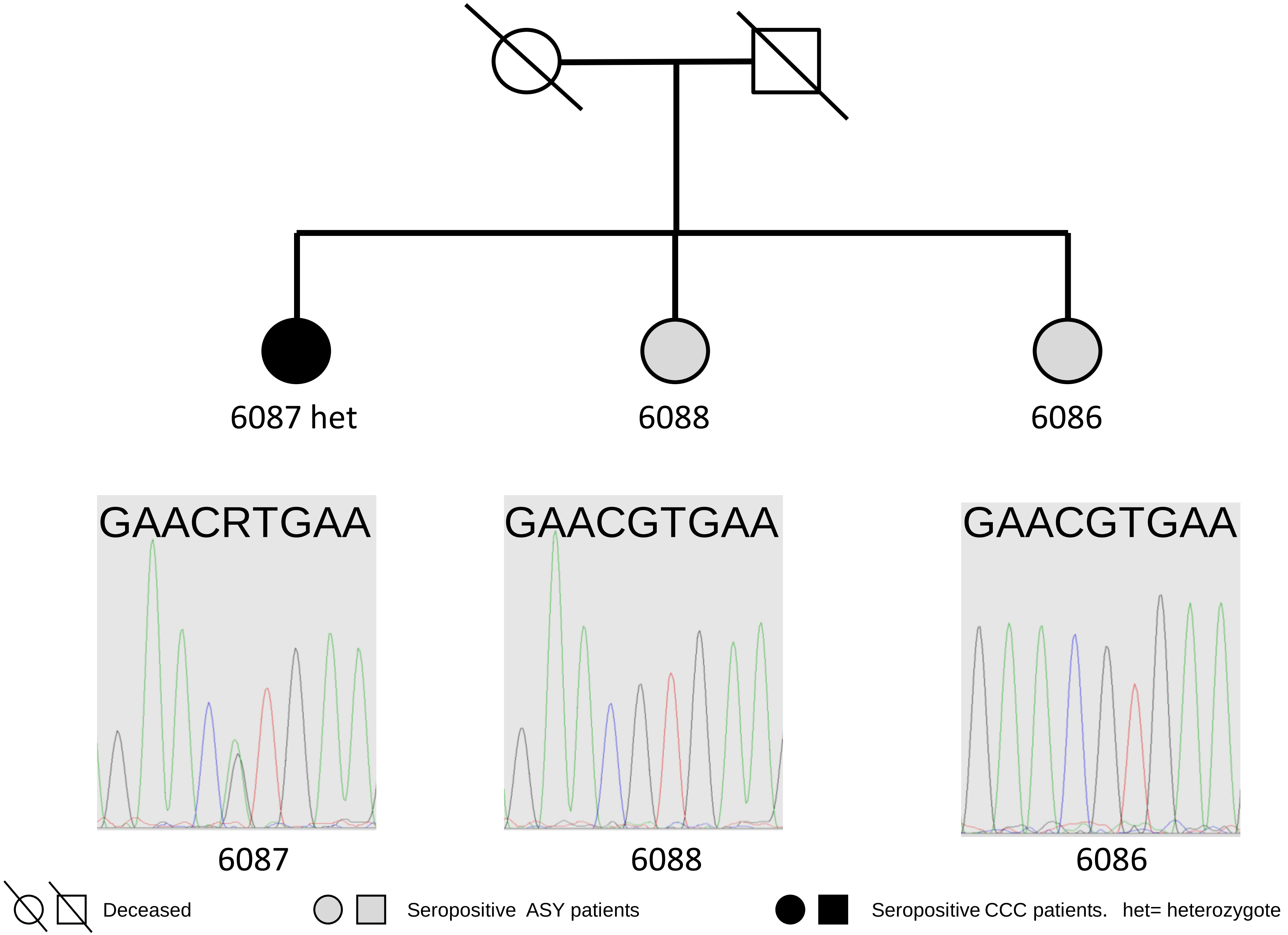

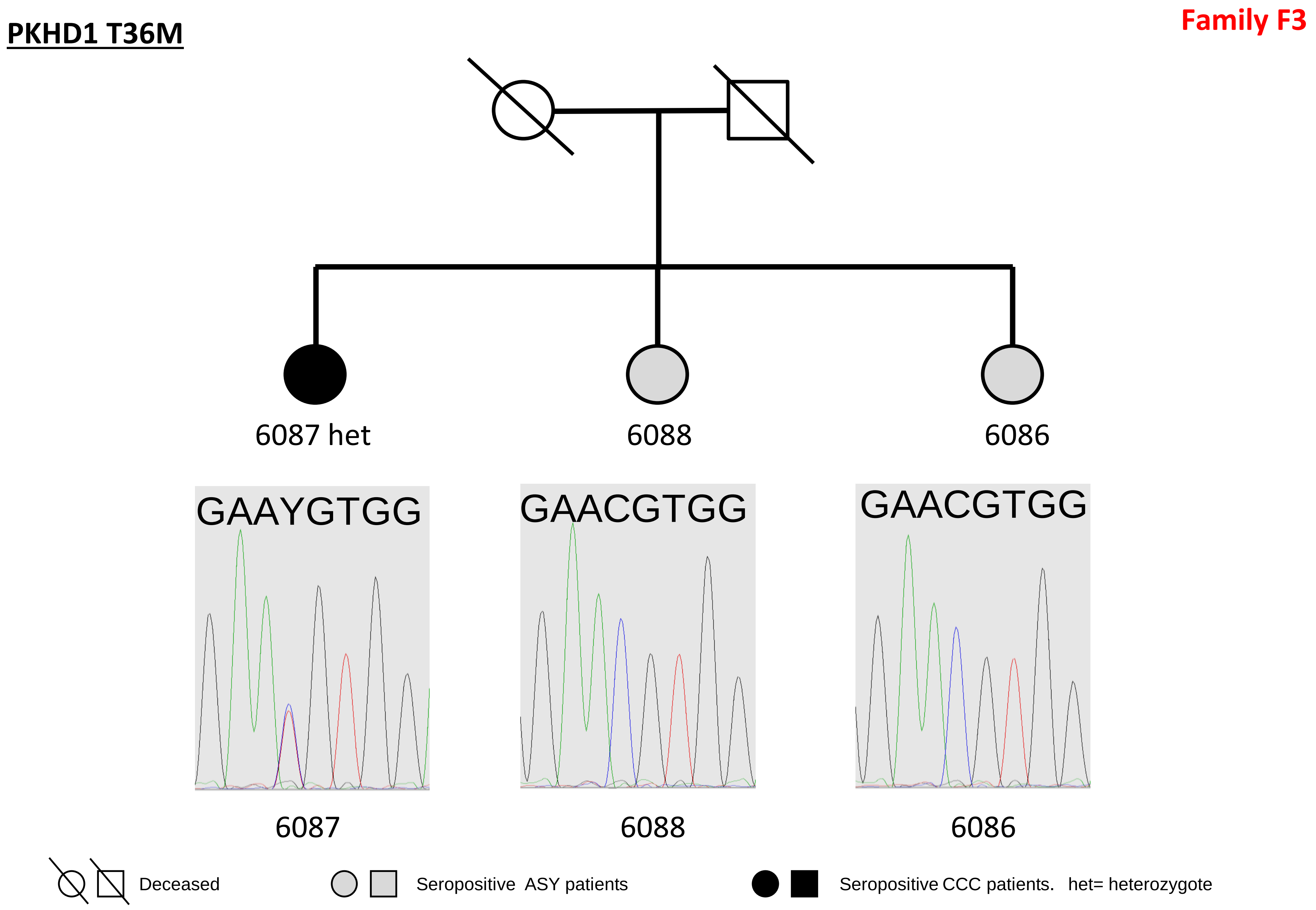
PKHD1 T36M
Family F3
6087 het
6088
6086
GAAYGTGG
GAACGTGG
GAACGTGG
6087
6088
6086
Deceased
Seropositive ASY patients
Seropositive CCC patients. het= heterozygote

RNLS R222H

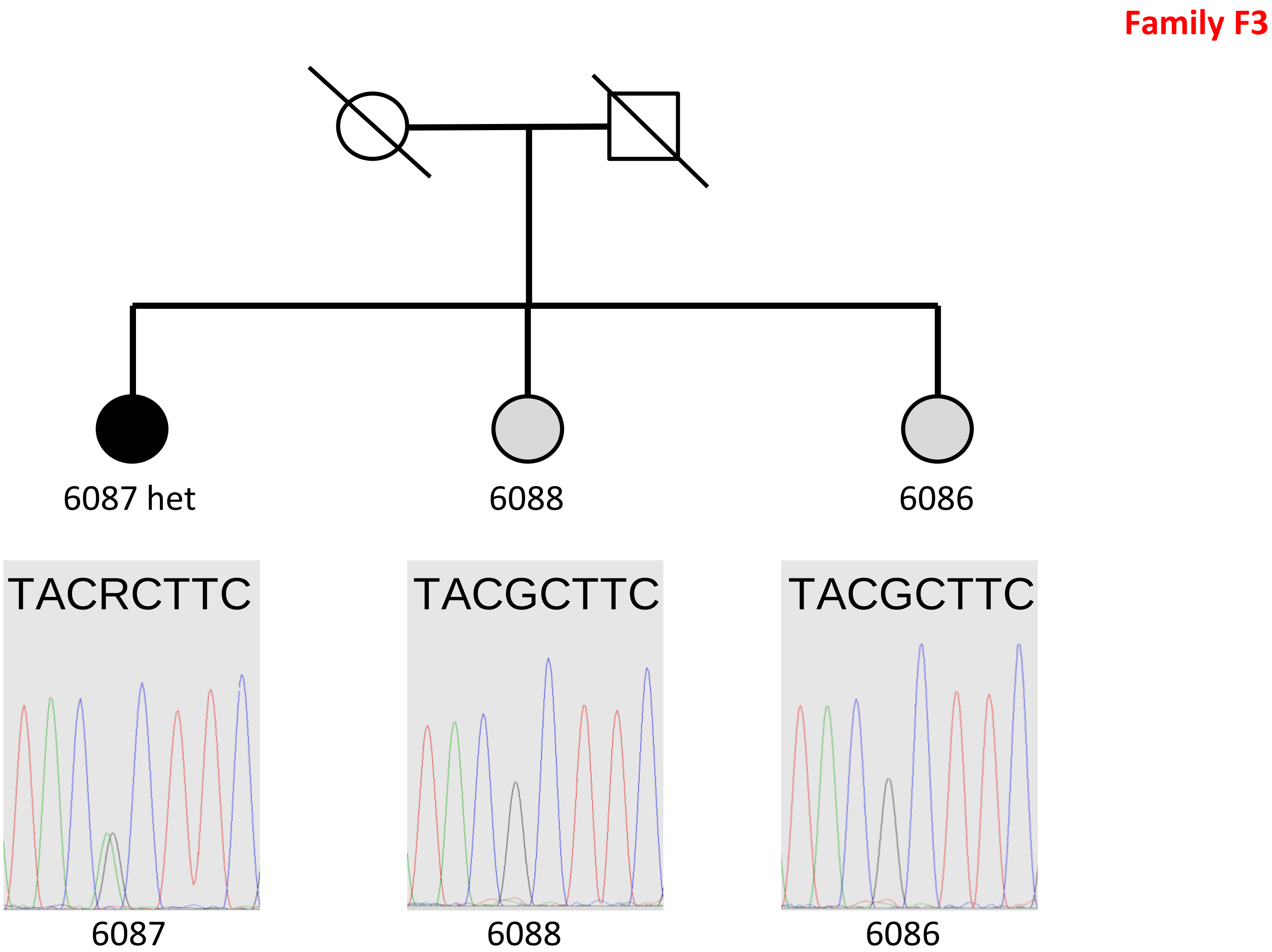

Deceased Seropositive ASY patients Seropositive CCC patients. het= heterozygote

GIT1 A745T

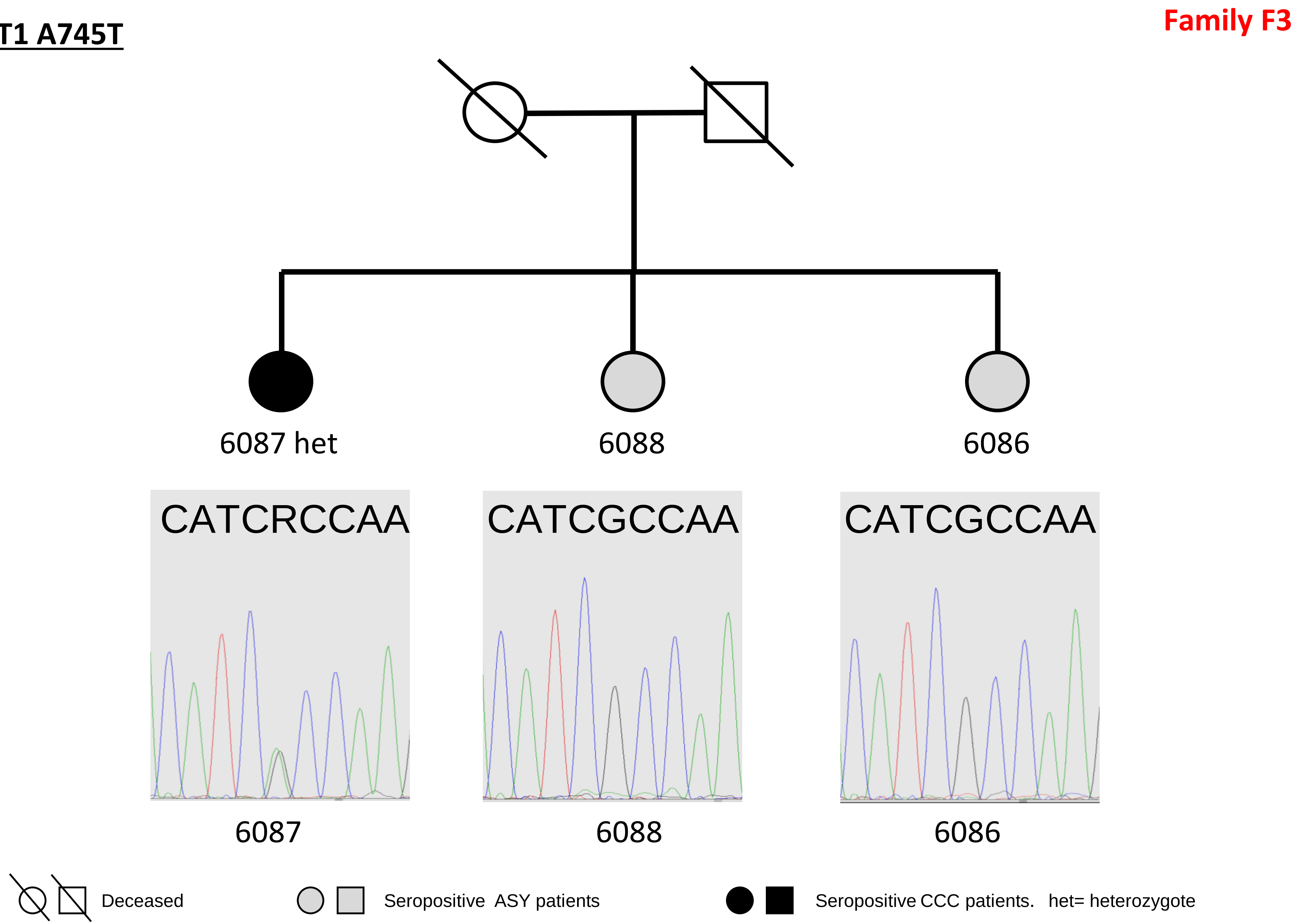
Family F3
6087 het
6088
6086
CATCRCCAA
CATCGCCAA
CATCGCCAA
6087
6088
6086
Deceased
Seropositive ASY patients
Seropositive CCC patients. het= heterozygote

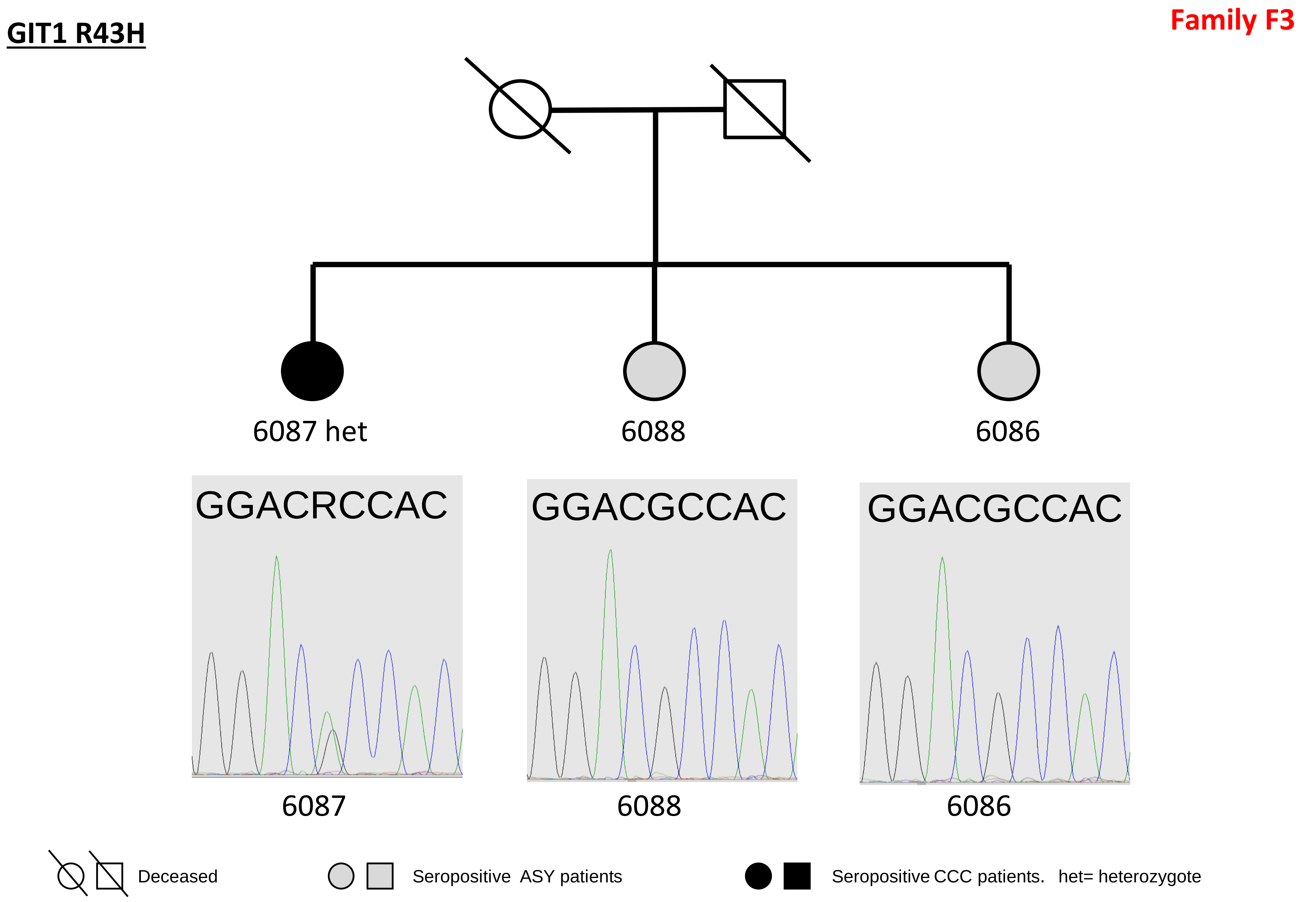
GIT1 R43H
Family F3
6087 het
6088
6086
GGACRCCAC
GGACGCCAC
GGACGCCAC
6087
6088
6086
Deceased
Seropositive ASY patients
Seropositive CCC patients. het= heterozygote

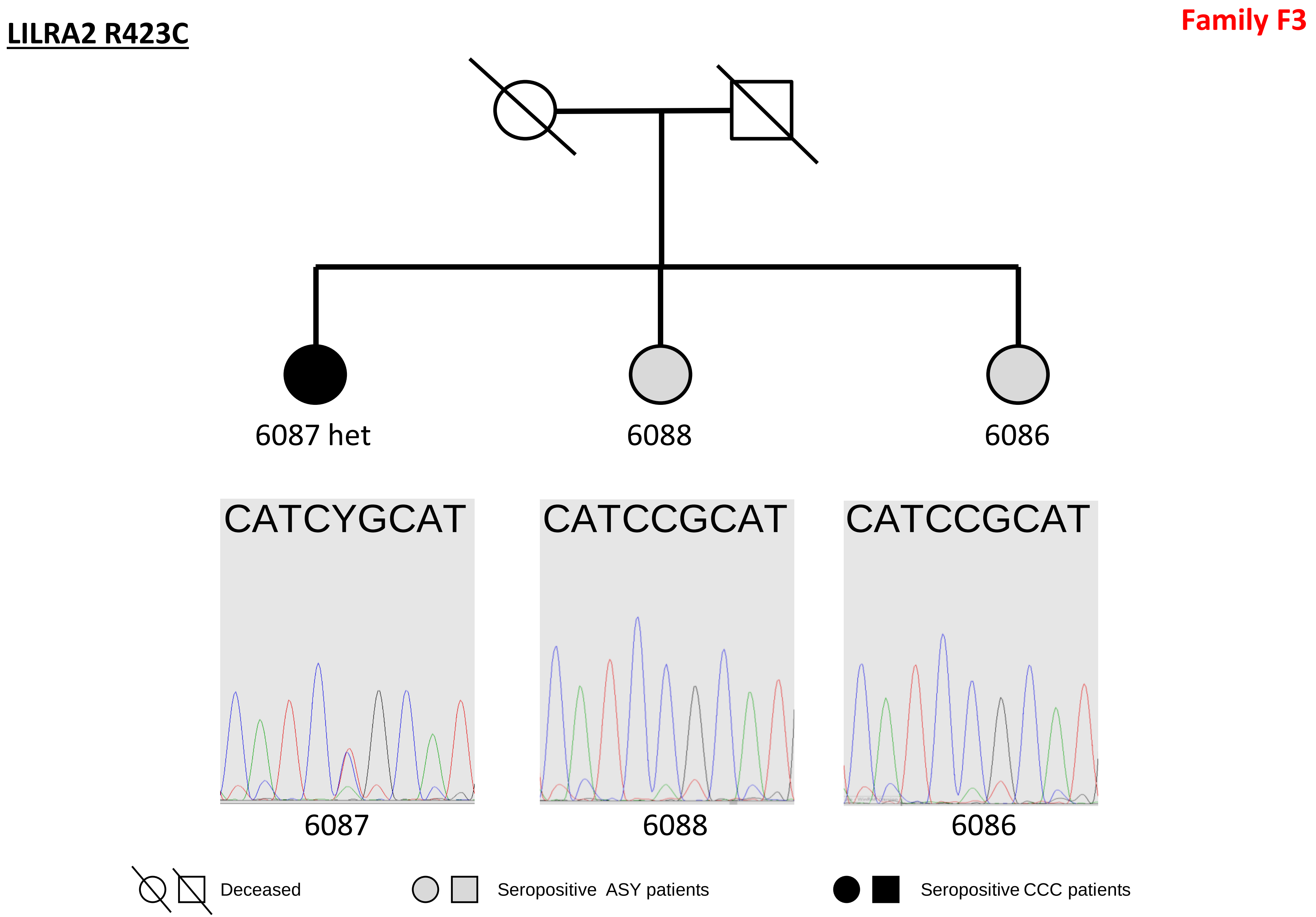
LILRA2 R423C
Family F3
6087 het
6088
6086
CATCYGCAT
CATCCGCAT
CATCCGCAT
6087
6088
6086
Deceased
Seropositive ASY patients
Seropositive CCC patients

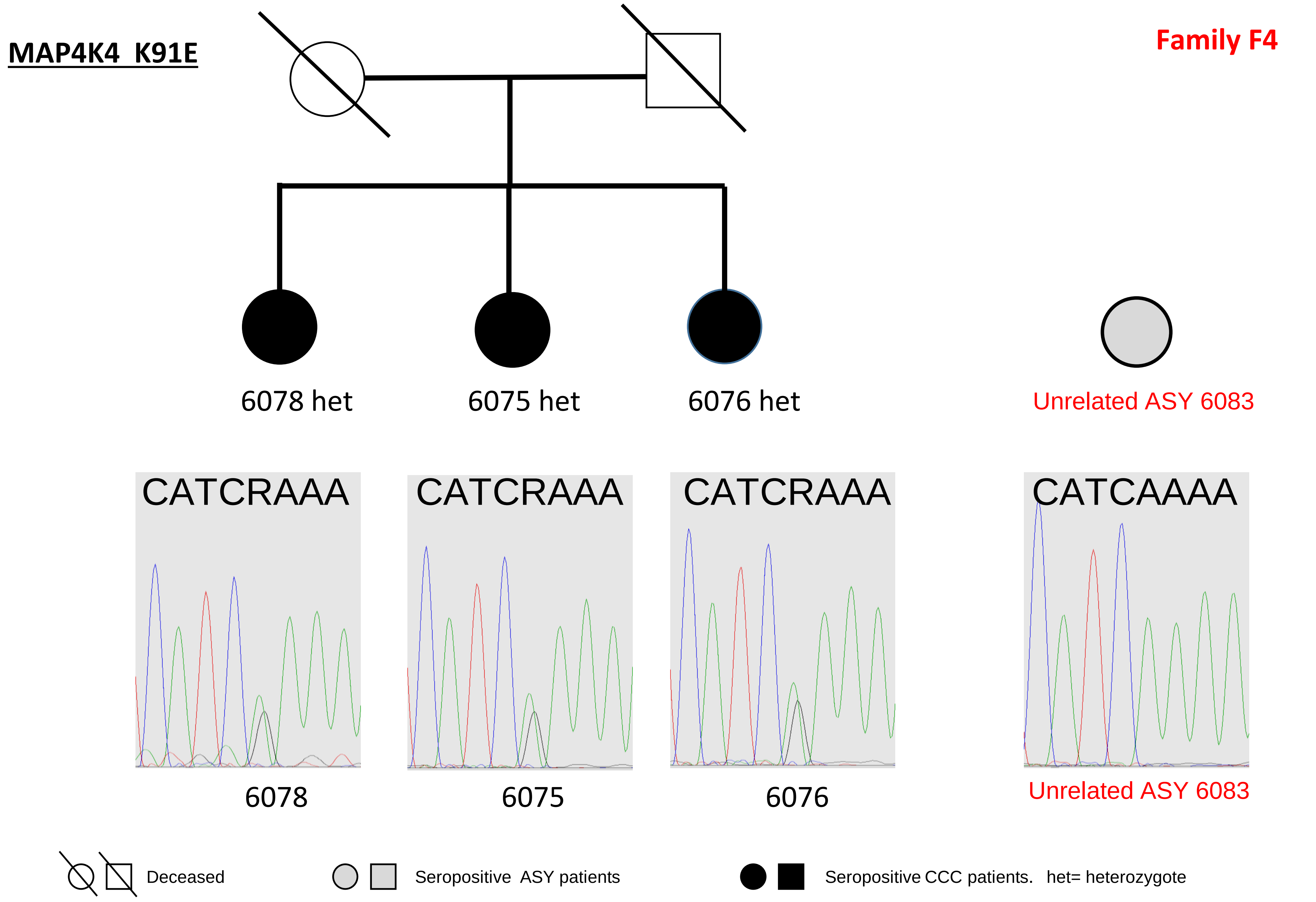
MAP4K4 K91E
Family F4
6078 het
6075 het
6076 het
Unrelated ASY 6083
CATCRAAA
CATCRAAA
CATCRAAA
CATCAAAA
6078
6075
6076
Unrelated ASY 6083
Deceased
Seropositive ASY patients
Seropositive CCC patients. het= heterozygote

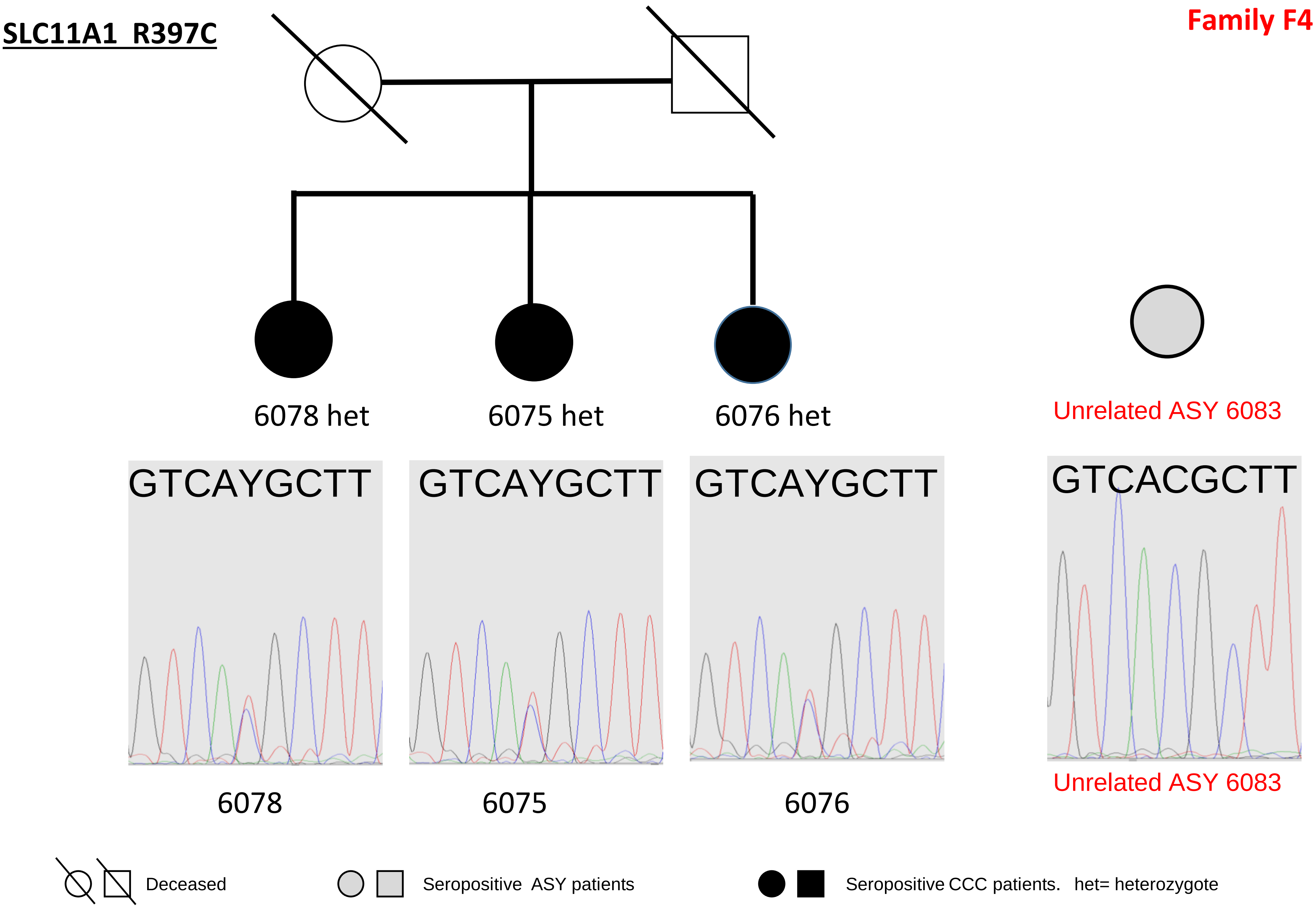
SLC11A1 R397C
Family F4
6078 het
6075 het
6076 het
Unrelated ASY 6083
GTCAYGCTT
GTCAYGCTT
GTCAYGCTT
GTCACGCTT
6078
6075
6076
Unrelated ASY 6083
Deceased
Seropositive ASY patients
Seropositive CCC patients. het= heterozygote

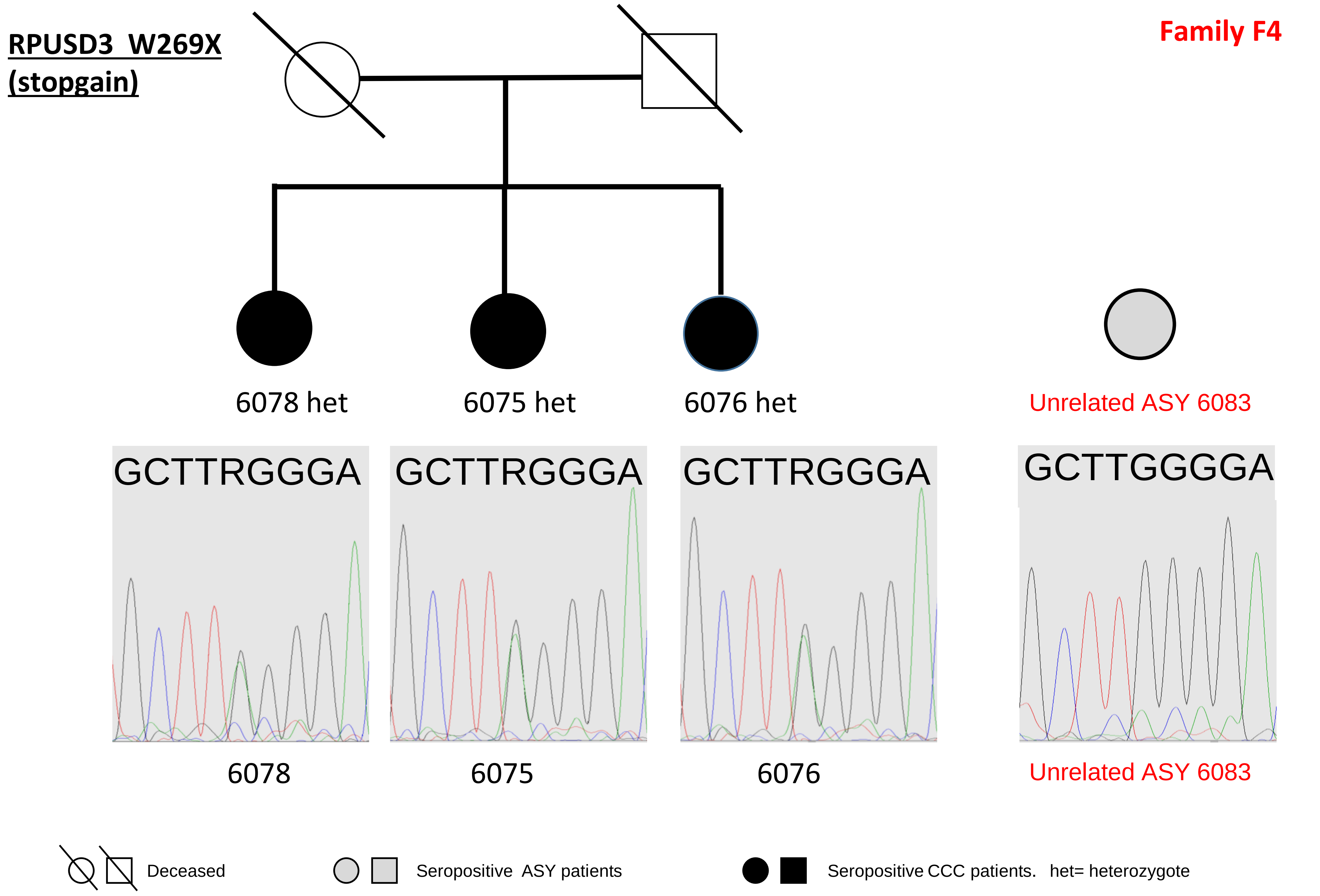

RPUSD3 W269X
(stopgain)
Family F4
6078 het
6075 het
6076 het
Unrelated ASY 6083
GCTTRGGGA
GCTTRGGGA
GCTTRGGGA
GCTTGGGGA
6078
6075
6076
Unrelated ASY 6083
Deceased
Seropositive ASY patients
Seropositive CCC patients. het= heterozygote

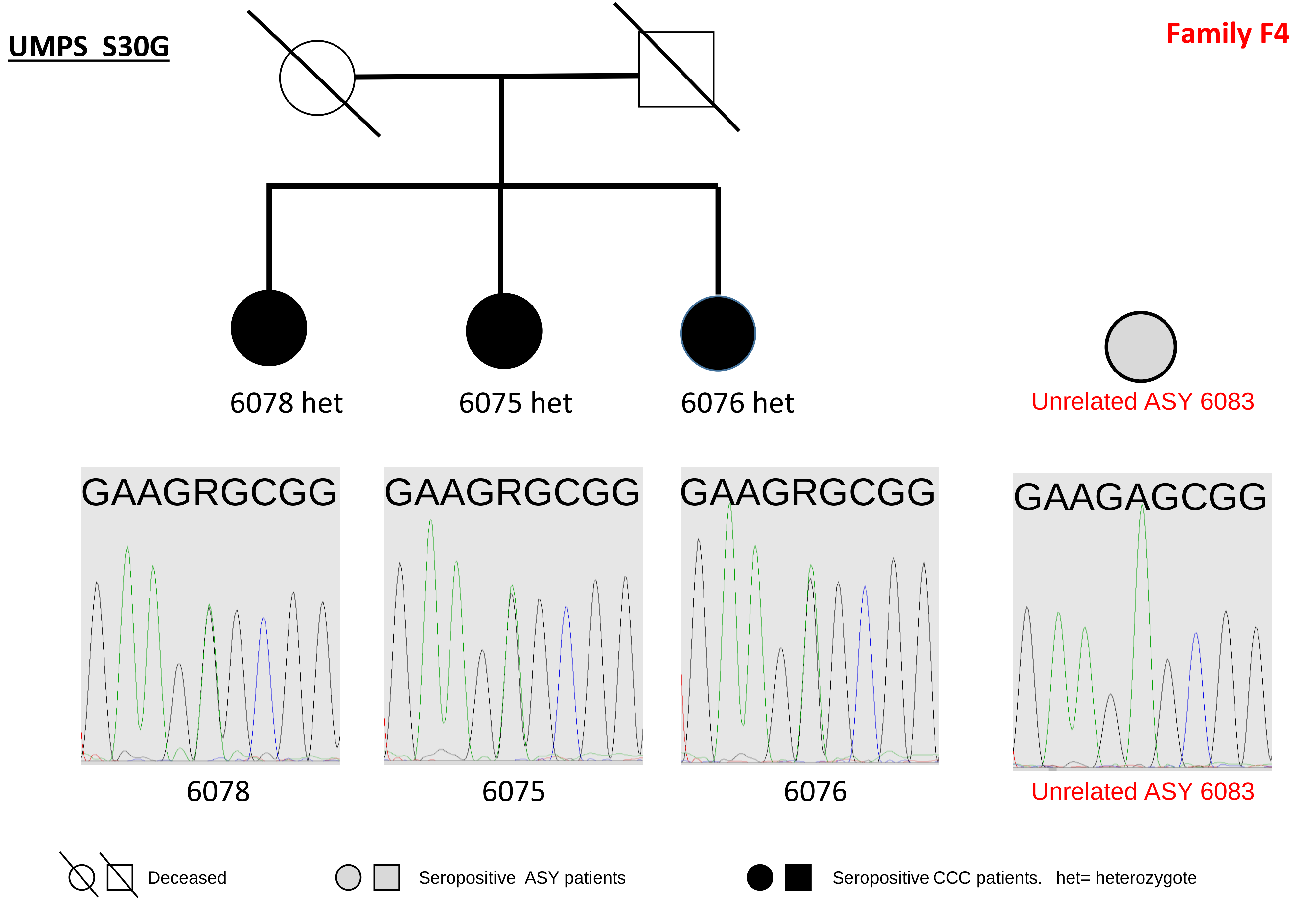
UMPS S30G
Family F4
6078 het
6075 het
6076 het
Unrelated ASY 6083
GAAGRGCGG
GAAGRGCGG
GAAGRGCGG
GAAGAGCGG
6078
6075
6076
Unrelated ASY 6083
Deceased
Seropositive ASY patients
Seropositive CCC patients. het= heterozygote

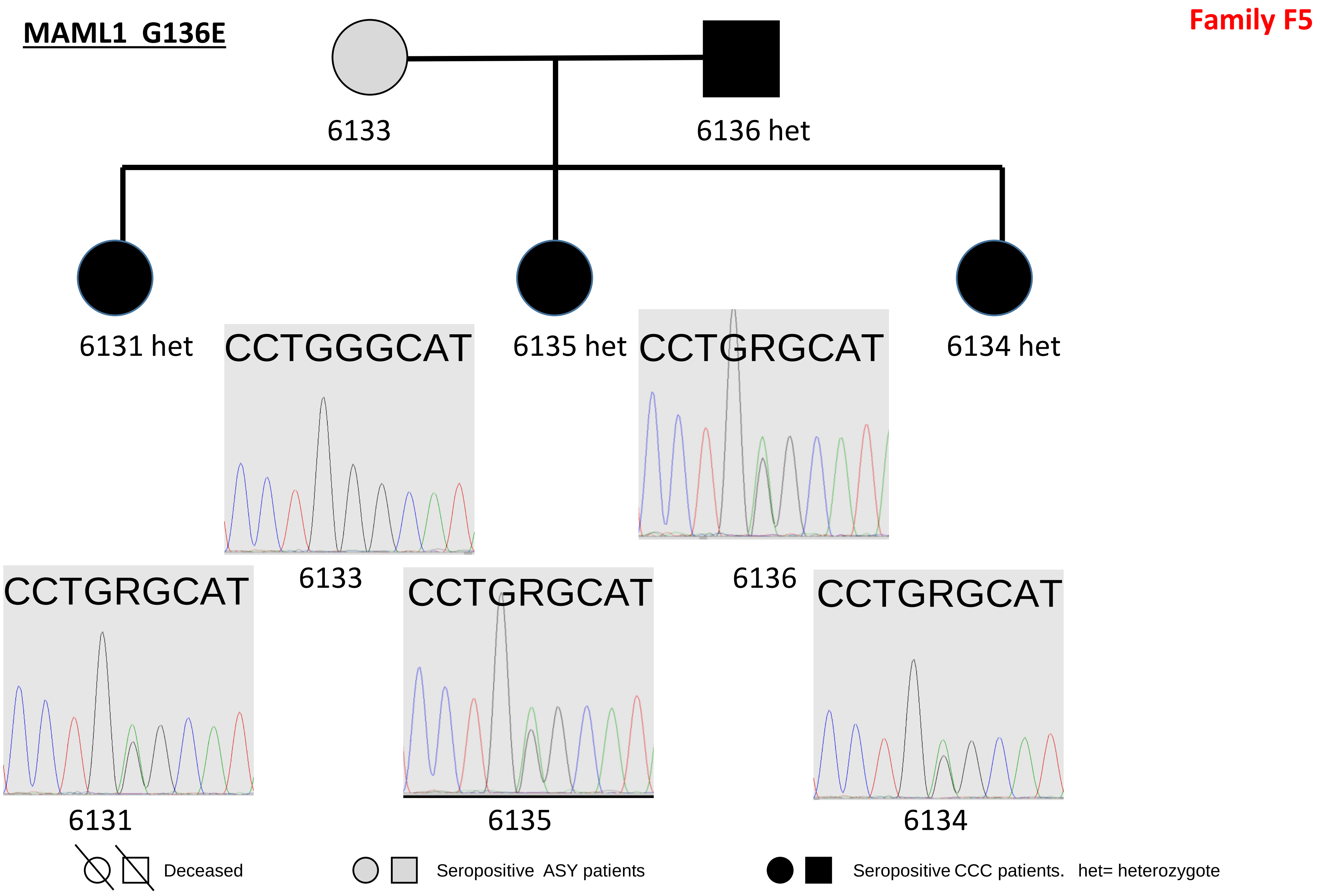
MAML1 G136E
Family F5
6133
6136 het
6131 het
CCTGGGCAT
6135 het
CCTGRGCAT
6134 het
CCTGRGCAT
6133
CCTGRGCAT
6136
CCTGRGCAT
6131
6135
6134
Deceased
Seropositive ASY patients
Seropositive CCC patients. het= heterozygote

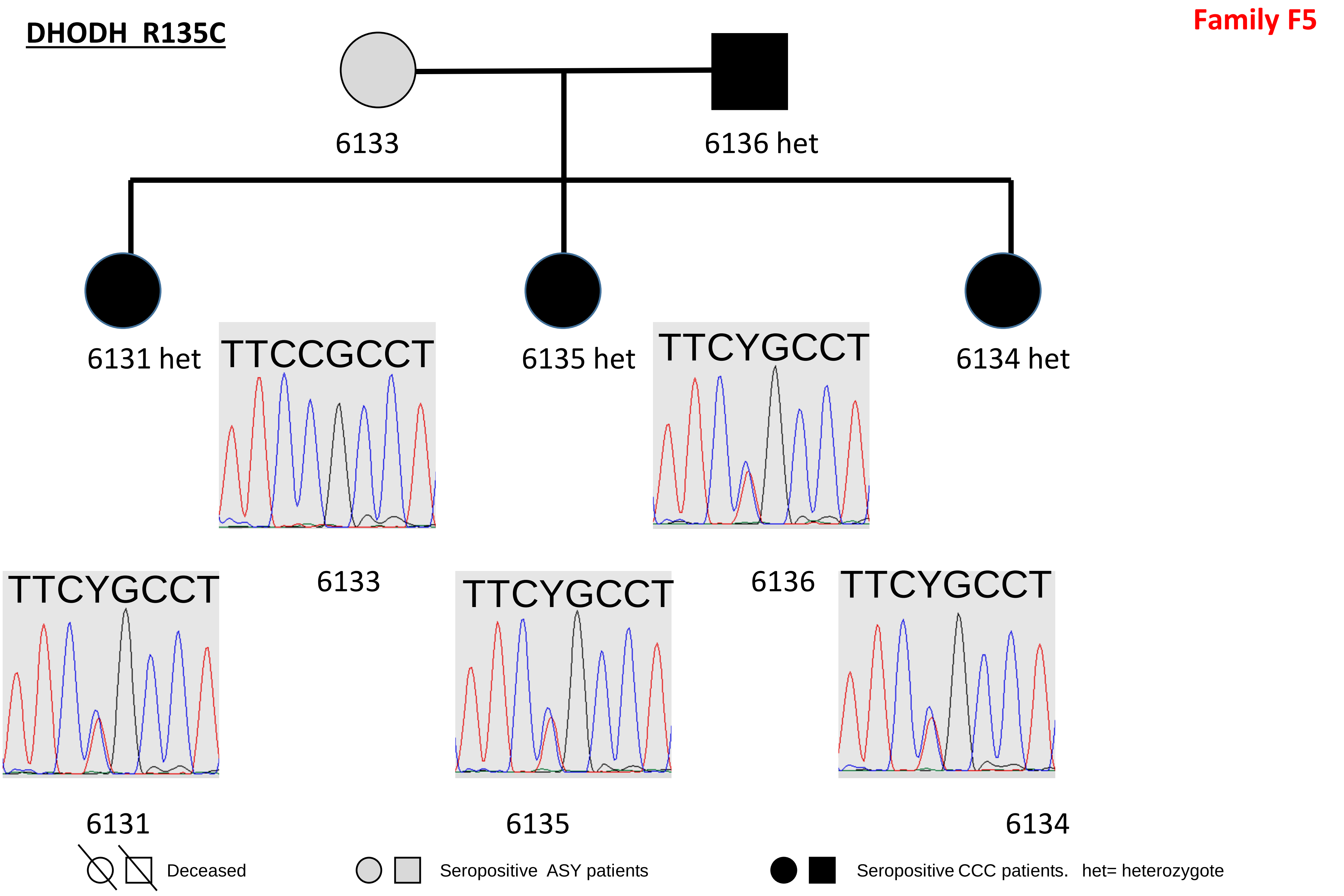

DHODH R135C
Family F5
6133
6136 het
6131 het
TTCCGCCT
6135 het
TTCYGCCT
6134 het
TTCYGCCT
6133
TTCYGCCT
6136
TTCYGCCT
6131
6135
6134
Deceased
Seropositive ASY patients
Seropositive CCC patients. het= heterozygote

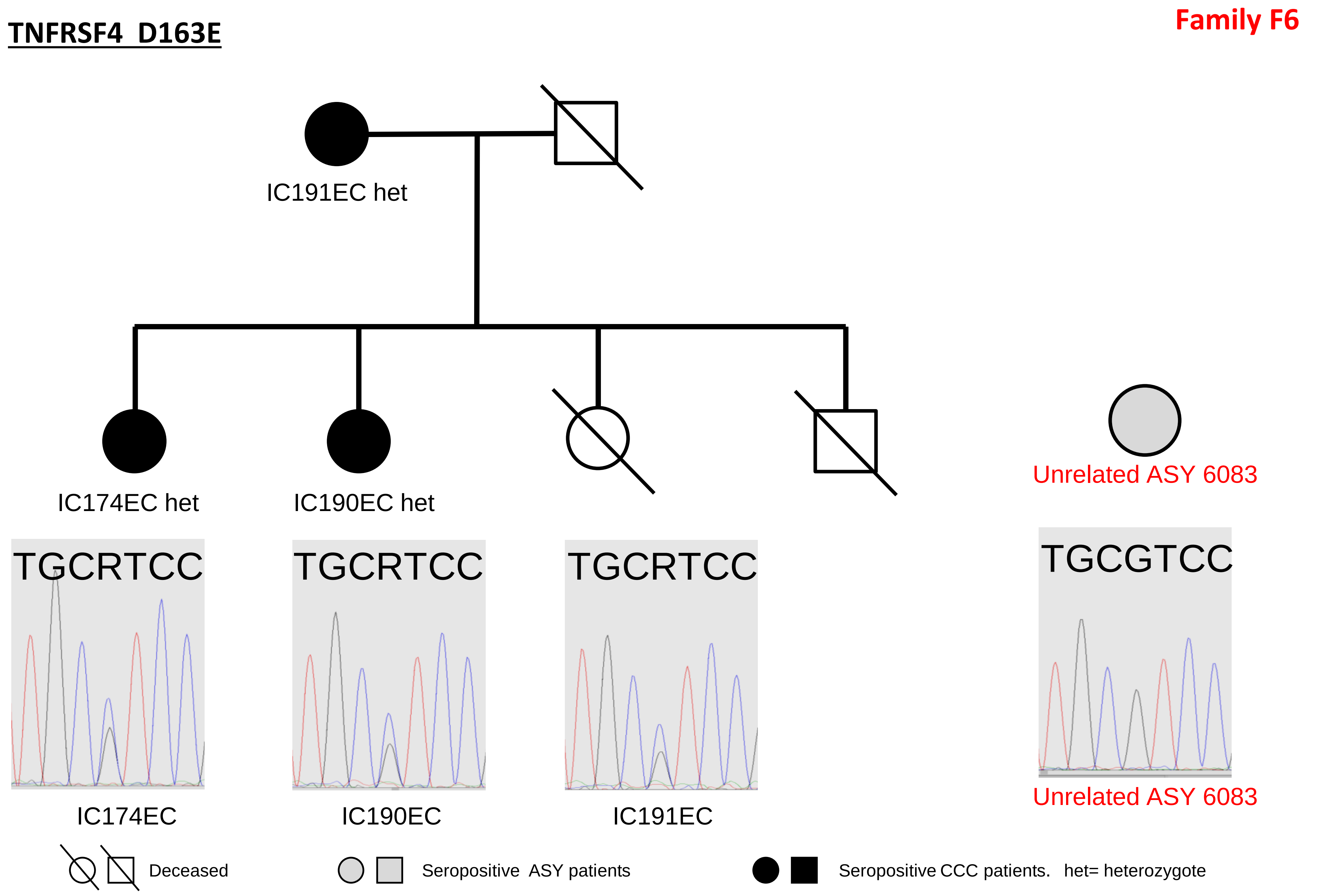

TNFRSF4 D163E
Family F6
IC191EC het
IC174EC het
IC190EC het
Unrelated ASY 6083
TGCRTCC
TGCRTCC
TGCRTCC
TGCGTCC
IC174EC
IC190EC
IC191EC
Unrelated ASY 6083
Deceased
Seropositive ASY patients
Seropositive CCC patients. het= heterozygote

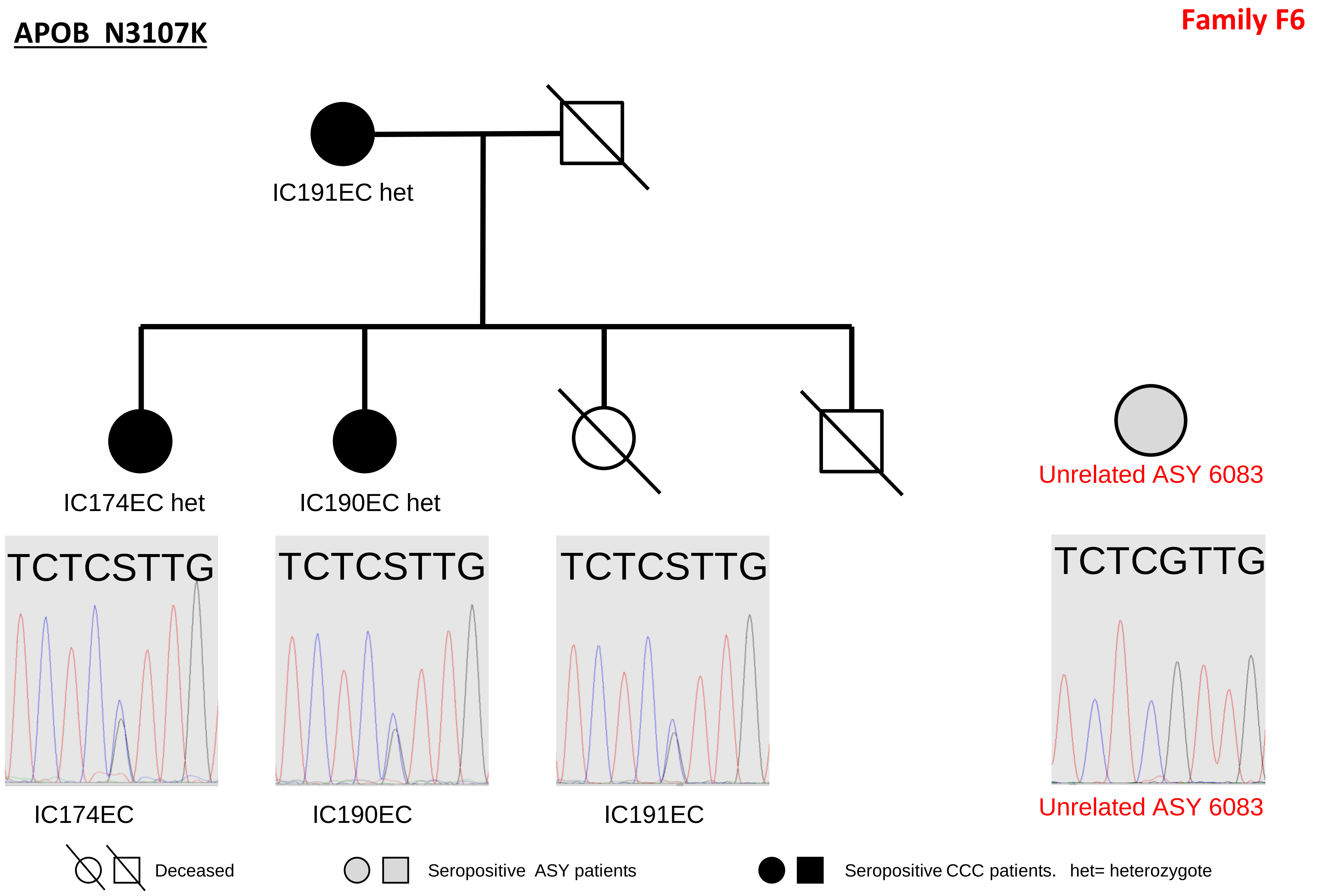
APOB N3107K
Family F6
IC191EC het
IC174EC het
IC190EC het
Unrelated ASY 6083
TCTCSTTG
TCTCSTTG
TCTCSTTG
TCTCGTTG
IC174EC
IC190EC
IC191EC
Unrelated ASY 6083
Deceased
Seropositive ASY patients
Seropositive CCC patients. het= heterozygote

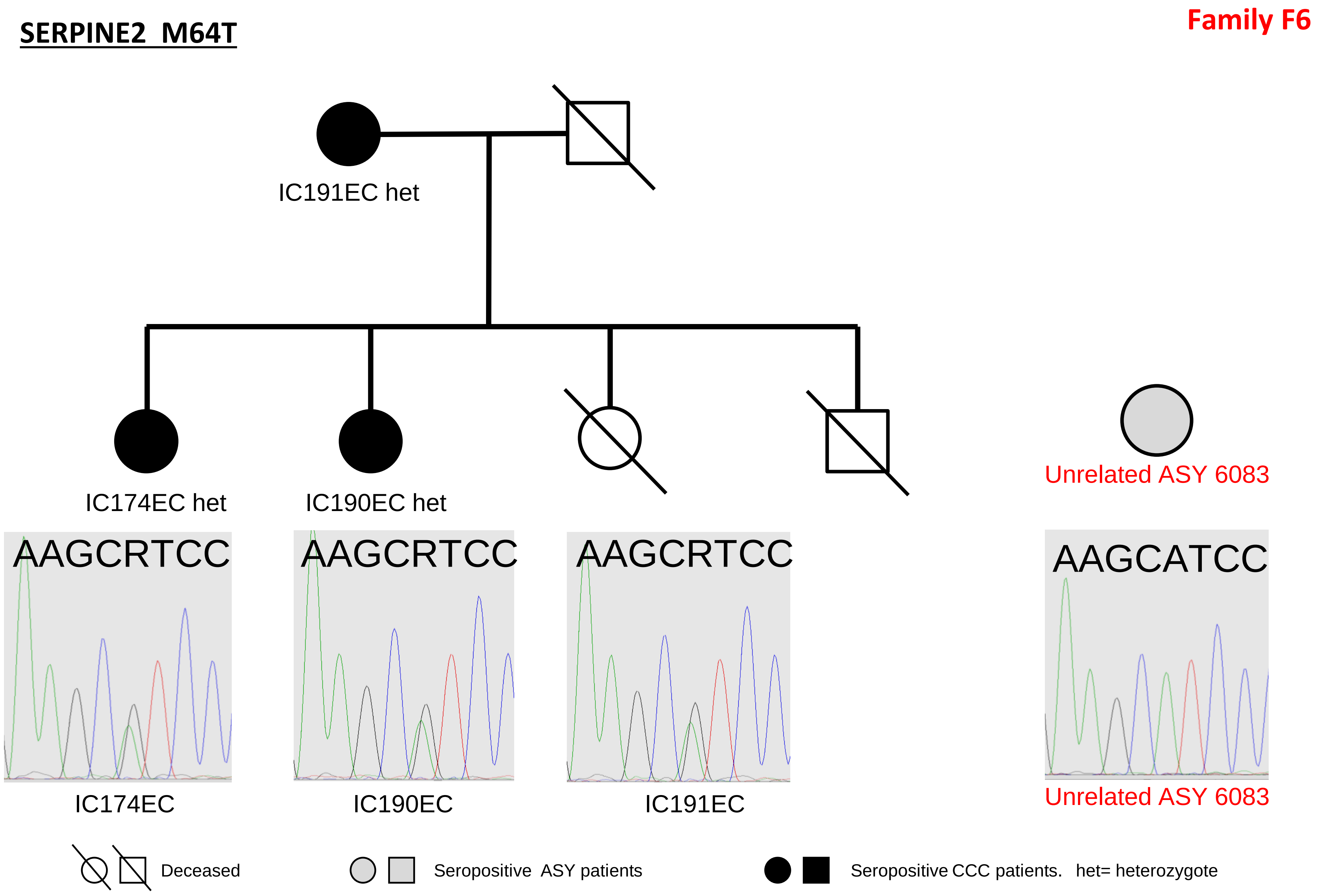
SERPINE2 M64T
Family F6
IC191EC het
IC174EC het
IC190EC het
Unrelated ASY 6083
AAGCRTCC
AAGCRTCC
AAGCRTCC
AAGCATCC
IC174EC
IC190EC
IC191EC
Unrelated ASY 6083
Deceased
Seropositive ASY patients
Seropositive CCC patients. het= heterozygote
