# Supplementary material for: Rare Pathogenic Variants in Mitochondrial and Inflammation-Associated Genes May Lead to Inflammatory Cardiomyopathy in Chagas Disease
Source: J Clin Immunol. 2021 Mar 3;41(5):1048–63. doi: 10.1007/s10875-021-01000-y (PMC8249271; doi:10.1007/s10875-021-01000-y)
Supplement: Supplementary file 2 — (DOCX 12 kb) [file 10875_2021_1000_MOESM2_ESM.docx]

**Online table 1:** The major ECG abnormalities defining CCC cases modified from the Minnesota Code Classification, as modified by Ribeiro et al. (11):

_______________________________________________________________________

- Old myocardial infarction (MI) (major Q-wave abnormalities [MC 1.1.x or 1.2.x])

- Possible MI (minor Q-waves abnormalities with ST segment or T-wave abnormalities [1.3.x and 4.1.x, 4.2, 5.1, or 5.2])

- Complete intraventricular blocks (7.1, 7.2, 7.4, or 7.8)

- Frequent supraventricular or ventricular premature beats (MC 8.1.x, except 8.1.4)

- Major isolated ST segment or T-wave abnormalities (MC 4.1.x, 4.2, 5.1 or 5.2)

-Atrial fibrillation or flutter or supraventricular tachycardia (MC 8.3.x. or 8.4.2)

- Other major arrhythmias (MC 8.2.x, except 8.2.1)

- Major atrioventricular conduction abnormalities or pacemaker use (MC 6.1, 6.2.x, 6.4, 6.8, 8.6.1 or 8.6.2)

- Major QT prolongation (>115%)

- Left ventricular hypertrophy (LVH) (MC 3.1 together with [4.1.x, 4.2, 5.1, or 5.2])

**______________________________________________________________________**
