# Supplementary material for: Rare Pathogenic Variants in Mitochondrial and Inflammation-Associated Genes May Lead to Inflammatory Cardiomyopathy in Chagas Disease
Source: J Clin Immunol. 2021 Mar 3;41(5):1048–63. doi: 10.1007/s10875-021-01000-y (PMC8249271; doi:10.1007/s10875-021-01000-y)
Supplement: Supplementary file 3 — (DOCX 16 kb) [file 10875_2021_1000_MOESM3_ESM.docx]

**Online table 2:** **Description of the primers used to confirm the variants by Sanger sequencing.**

| **Family** | **Gene**  **symbol** | **rs number**  **(db147)** | **Variant** | **Forward primer/**  **Reverse primer** | **Amplicon**  **Size (bp)** |
| --- | --- | --- | --- | --- | --- |
| Family 1 | LEPR | rs34499590 | Chr 1 66081791 C/G | CCCTGCCTCCTAATAAGAAATGT  CAATGGATTACCACTCTGTACCTC | 350 |
| Family 1 | ADCY10 | rs140663029 | Chr 1 167830254 C/T | GAAGCAGCAATAAAGAACCATTG  TTACCTGAACATGGAAAATGTCA | 349 |
| Family 1 | MOCS1 | rs148579886 | Chr 6 39877666 G/A | TAAGGAAGGCATATTGTCCTCA  ATGGAAGGAGAAGCATCACTGTA | 345 |
| Family 1 | ADGRG6 | rs184235213 | Chr 6 142724940 A/G | TTGGGGTAATTTTATGGGTGT  CTTGGAAGACCAATGCTAAAAT | 300 |
| Family 1 | AKAP13 | rs745783128 | Chr 15 86124694 A/G | AAAACTAGTGCCTGTGAGGTGAG  ACAGGATGTGCTTCATCGTCTAT | 329 |
|  |  |  |  |  |  |
| Family 2 | OBSCN | rs74623201 | Chr 1 228464267 G/T | GTTCTAAAGGCGGTGGTCTCAG  GAAGACCATAAGGCCACTATCCT | 284 |
|  |  |  |  |  |  |
| Family 3 | APOB | . | Chr 2 21247996 T/C | GCTTAGAGGAATTAGCCTGGACT  AGGAGCTGGAGGTCATGGAG | 297 |
| Family 3 | MRPS18B | rs116524936 | Chr 6 30590612 C/T | AGTGAATTGATGAAGCCAAACTG  TTAGGCTATAAGCAGGAGCCACT | 345 |
| Family 3 | PKHD1 | rs137852944 | Chr 6 51947999 C/T | TTCTGAGGCAGGTTAAATATTGC  TCTGTGACATTAGCAAGCCAGTA | 347 |
| Family 3 | RNLS | rs191733133 | Chr 10 90122344 C/G | TTTCTTCCACAGTAATTAGTGAATGC  TGGAGTTAAATGCTTGCTCTGTT | 298 |
| Family 3 | GIT1 | . | Chr 17 27901773 G/A | GATCTCAGCTGGTGGCTTAAAGT  GTGGGGATTAATGTCTGGAGTG | 348 |
| Family 3 | GIT1 | . | Chr 17 27910559 G/A | GACTGAGTGAGTGAATGTGTGGA  ATTCCAGATGGGGGTGTATGT | 345 |
| Family 3 | LILRA2 | rs149580797 | Chr 19 55098715 G/A | ACAGAGGGTCAGGTCCTGTAAA  TGTAGGTCCTAGATTGTCCTCCA | 346 |
|  |  |  |  |  |  |
| Family 4 | MAP4K4 | . | Chr 2 102440480 A/G | TGGAGAACTTGTGTTCCTTTCAT  CTGCCCCTCTTTATTGTTTTTCT | 342 |
| Family 4 | SLC11A1 | rs74906275 | Chr 2 219257728 C/G | CCAGTTCAACAGTGGAAAAACAG  CATCTCACCAGCAGGCTCT | 284 |
| Family 4 | RPUSD3 | rs142984515 | Chr 3 9880802 A/G | GATCTGAGAAAGGGGGTGGT  ACAGATAAGGTCTGGAGATGCTG | 330 |
| Family 4 | UMPS | rs17843776 | Chr 3 124449406 C/T | GGGAAAGCTGCAGACGAG  ACTCCTGTCACCCTCTTCCAT | 346 |
|  |  |  |  |  |  |
| Family 5 | MAML1 | rs146382198 | Chr 5 179192418 G/A | TGGCTTGTTACTGTCATAGCAGA  AGAGAGTGCTTCCCACTGGACT | 295 |
| Family 5 | DHODH | rs201230446 | Chr 16 72048540 C/A | TTGCTGCAGGATTTGACAAG  ATGCCTGGGGCACATAGTAG | 323 |
|  |  |  |  |  |  |
| Family 6 | TNFRSF4 | . | Chr 1 1147467 C/T | CTGGTGTCTGTGGTAGATGCTG  CCCTTCTCCTATTCGGGTTG | 367 |
| Family 6 | APOB | rs72653101 | Chr 2 21230419 C/T | TGAAAGTTCGTTTTCCATTAAGGT  TTTCCCATAGAGAGAAATCTTTCAG | 297 |
| Family 6 | SERPINE2 | rs34078713 | Chr 2 224866427 G/T | CCATCTGCTCCCACTTCAAT  CAGCAAAAACCAAGCCATAAAC | 291 |
