# Supplementary material for: Rare Pathogenic Variants in Mitochondrial and Inflammation-Associated Genes May Lead to Inflammatory Cardiomyopathy in Chagas Disease
Source: J Clin Immunol. 2021 Mar 3;41(5):1048–63. doi: 10.1007/s10875-021-01000-y (PMC8249271; doi:10.1007/s10875-021-01000-y)
Supplement: Supplementary file 4 — (DOCX 37 kb) [file 10875_2021_1000_MOESM4_ESM.docx]

**Online table 3: Pathway/process gene lists.**

| **Inflammation genes (n=2768)** | |
| --- | --- |
|  | A1BG, A4GNT, ABCA1, ABCA3, ABCA4, ABCB1, ABCB10, ABCB11, ABCB4, ABCC1, ABCC2, ABCC4, ABCD1, ABCD2, ABCF1, ABCG2, ABHD12, ABHD6, ABL1, ABR, ACADVL, ACAN, ACE, ACE2, ACHE, ACLY, ACO1, ACO2, ACOT1, ACOT11, ACOX1, ACOX2, Acp5, ACSL1, ACTA1, ACTA2, ACTB, ACTL6A, ACTN4, ACVR1, ADA, ADAM10, ADAM12, ADAM15, ADAM17, ADAM8, ADAM9, ADAMTS1, ADAMTS10, ADAMTS12, ADAMTS13, ADAMTS14, ADAMTS15, ADAMTS16, ADAMTS17, ADAMTS18, ADAMTS19, ADAMTS2, ADAMTS20, ADAMTS3, ADAMTS4, ADAMTS5, ADAMTS6, ADAMTS7, ADAMTS9, ADCY10, ADCY8, ADD2, ADGRG6, ADIPOQ, ADIPOR1, ADIPOR2, ADK, ADM, ADORA1, ADORA2A, ADORA2B, ADORA3, ADRA1A, ADRA1B, ADRA1D, ADRA2A, ADRA2B, ADRA2C, ADRB1, ADRB2, ADRB3, ADSS, AFAP1L2, AGER, AGR2, AGT, AGTR1, AGTR2, AHCY, AHI1, AHNAK, AHR, AHSG, AIF1, AIM2, AIMP1, AIRE, AK2, AKAP12, AKAP13, AKR1A1, AKR1D1, AKT1, AKT2, ALAS2, ALB, ALCAM, ALDH1A1, ALDH2, ALDOA, ALOX12, ALOX15, ALOX5, ALOX5AP, ALPK2, ALPL, ALPP, ALS2, AMACR, AMBP, AMPD1, AMPD3, ANAPC2, ANG, ANGPT1, ANGPT2, ANGPTL2, ANGPTL4, ANGPTL6, ANKH, ANO6, ANXA1, ANXA2, ANXA3, ANXA4, ANXA5, ANXA7, AOAH, AOC3, AOX1, AP3B1, APAF1, APC, APCS, APLP2, APOA1, APOA2, APOA4, APOB, APOE, APOH, APOL3, APOM, APP, APRT, AQP1, AQP4, AQP9, AR, ARAP2, ARAP3, ARCN1, AREG, ARF1, ARFGAP1, ARFGAP3, ARG1, ARHGAP10, ARHGAP25, ARHGAP33, ARHGDIA, ARHGDIB, ARHGEF7, ARID5A, ARIH1, ARIH2, ARL16, ARRB1, ARRB2, ART1, ART3, ASAP1, ASB2, ASH1L, ASNS, ASPN, ATAT1, ATF3, ATF4, ATF6B, ATG16L1, ATG5, ATG7, ATIC, ATM, ATP11C, ATP1A1, ATP1B1, ATP2B1, ATP2C1, ATP4A, ATP4B, ATP8A1, ATXN1, AVP, AXL, AZGP1, AZU1, B2M, B3GALT4, B3GNT6, B4GALT1, BACH2, BAD, BAG4, BAG6, BAK1, BAP1, BARD1, BATF, BAX, BBS12, BCL11B, BCL2, BCL2A1, BCL2L1, BCL2L11, BCL3, BCL6, BCR, BDKRB1, BDKRB2, BDNF, BGLAP, BGN, BHLHE40, BHMT, BID, BIN3, BIRC2, BIRC3, BIRC5, BLK, BLMH, BLNK, BMF, BMP1, BMP2, BMP4, BMP5, BMP6, BMP7, BMP8A, Bmp8b, Bmpr1b, BMX, BPI, BPIFA1, BRD2, BRD3, BRD4, BRF2, BRMS1, BSG, BSN, BST1, BTC, BTLA, BTN1A1, BTNL2, C14orf2, C18orf8, C19orf57, C1QA, C1QBP, C1QTNF3, C1QTNF6, C1R, C2, C3, C3AR1, C3orf62, C5, C5AR1, C5orf30, C6, C6orf15, C6orf47, C6orf62, C9orf152, C9orf72, C9orf78, CA1, CA10, CA12, CA13, CA14, CA2, CA3, CA4, CA5A, CA5B, CA6, CA7, CA9, CACNA1A, CACNA1B, CACNA1C, CACNA1D, CACNA1F, CACNA1H, CACNA1S, CACNA2D1, CACNA2D2, CACNA2D3, CACNA2D4, CACNB2, CACNG1, CALCA, CALCRL, CALR, CAMK1, CAMK1D, CAMK2D, CAMLG, CAMP, CAPG, CAPN2, CAPN3, CAPZB, CARD11, CARD9, CASC3, CASP1, Casp12, CASP3, CASP4, CASP7, CASP8, CASR, CAST, CAT, CAV1, CAV3, CBFA2T2, CBL, CBLB, CBR1, CCDC59, CCDC7, CCDC88A, CCDC88B, CCHCR1, CCL1, CCL11, CCL13, CCL14, CCL15, CCL16, CCL17, CCL18, CCL19, CCL2, CCL20, CCL21, CCL22, CCL23, CCL24, CCL25, CCL26, CCL27, CCL28, CCL3, CCL3L3, CCL4, CCL5, Ccl7, Ccl8, CCNA2, CCNB1, CCND1, CCND3, CCR1, CCR10, CCR2, CCR3, CCR4, CCR5, CCR6, CCR7, CCR8, CCR9, CCRL2, CD14, CD151, CD163, CD180, CD19, CD1D, CD2, CD200, CD200R1, CD22, CD244, CD247, CD27, CD274, CD276, CD28, CD300C, CD300LF, Cd33, CD36, CD37, CD38, CD3D, CD4, CD40, CD40LG, CD44, CD46, CD47, CD48, CD5, CD55, CD59, CD5L, CD6, CD63, CD68, CD69, CD7, CD70, CD72, CD74, CD79A, CD79B, CD80, CD81, CD83, CD84, CD86, CD8A, CD9, CD99L2, CDA, CDC25A, CDC25B, CDC42, CDC42EP3, CDH1, CDH11, CDH13, CDH26, CDK19, CDK2, CDK2AP2, CDK5R1, CDK6, CDK9, CDKN1A, CDKN2A, CDKN2D, CDO1, CEACAM1, CEACAM3, CEACAM6, CEBPA, CEBPB, CEBPD, CEBPE, CEBPG, CELA1, CELA3B, CELF2, CELF4, CEP41, CERS6, CFB, CFH, CFL1, CFLAR, CFP, CFTR, CH25H, CHCHD2, CHD1, CHD4, CHEK2, CHGA, CHI3L1, CHIA, CHM, CHRM1, CHRM2, CHRM3, CHRM4, CHRM5, CHRNA1, CHRNA10, CHRNA2, CHRNA3, CHRNA4, CHRNA5, CHRNA6, CHRNA7, CHRNA9, CHRNB1, CHRNB2, CHRNB3, CHRNB4, CHRND, CHRNE, CHRNG, CHST8, CHSY1, CHUK, CIITA, CILP, CINP, CIRBP, CISH, CKB, CKLF, CKM, CLCA1, CLCF1, CLCN5, CLCN7, CLEC12A, CLEC1B, CLEC3B, CLEC4A, CLEC4D, CLEC4E, CLEC4G, CLEC4M, CLEC5A, CLEC7A, CLEC9A, CLOCK, CLSTN2, CLU, CMKLR1, CNN3, CNR1, CNR2, CNTF, CNTN2, CNTNAP4, COCH, COIL, COL10A1, COL11A1, COL11A2, COL13A1, Col17a1, COL18A1, COL1A1, COL1A2, COL2A1, COL3A1, COL4A1, COL4A2, COL4A3, COL4A4, COL4A5, COL4A6, COL9A1, COL9A2, COL9A3, COLQ, COMP, CORO1A, CORT, COTL1, COX15, COX4I2, CPA5, CPB2, CPE, CPN1, CPNE1, CPNE7, CPVL, CR1, CR1L, CR2, CRBN, CREB1, CREB3, CREB3L3, CREG2, CRH, CRHBP, CRHR2, CRLF2, CROCC, CRP, CRTC2, CRTC3, CRY1, CRY2, CRYAB, CSF1, CSF1R, CSF2, CSF2RA, CSF2RB, CSF3, CSF3R, CSGALNACT1, CSK, CSPG4, CST3, CST5, CST6, CTF1, CTGF, CTLA4, CTNNA3, CTNNB1, CTNND1, CTNS, CTR9, CTRC, CTSB, CTSC, CTSD, CTSE, CTSG, CTSS, CUZD1, CX3CL1, CX3CR1, CXADR, CXCL1, CXCL10, CXCL11, CXCL12, CXCL13, CXCL14, CXCL16, CXCL17, CXCL2, CXCL3, CXCL5, CXCL6, Cxcl9, CXCR1, CXCR2, CXCR3, CXCR4, CXCR5, CYB5B, CYB5D1, CYBA, CYBB, CYC1, CYLD, CYP11A1, CYP11B1, CYP17A1, CYP19A1, CYP1A1, CYP1A2, CYP26B1, CYP2B6, CYP2C9, CYP2E1, CYP3A5, CYP3A7, CYP4F11, CYP4F3, CYP51A1, CYR61, CYSLTR1, CYSLTR2, CYTIP, CYTL1, CYYR1, DAPK1, DAPK2, DAXX, DCN, DCTN1, DCUN1D1, DDIT3, DDIT4, DDR1, DDR2, DDT, DDX25, DDX39B, DDX41, DDX5, DDX58, DEDD2, DEF6, DEFB1, DEFB114, DEFB116, DEK, DEPDC5, DHFR, DHODH, DHX16, DHX9, DIAPH1, DICER1, DIP2C, DKK1, DLL1, DLL4, DMBT1, DMD, DNAJA4, DNAJC4, DNASE1, DNASE2, DNM1, DNM1L, DOCK2, DOCK5, DOK6, DPAGT1, DPCR1, DPEP1, DPP4, DPYD, DPYS, DPYSL2, DRD1, DRD2, DRD3, DRD4, DRD5, DROSHA, DSC1, DSG1, DSG3, DTX1, DUOXA1, DUOXA2, DUSP1, DUSP10, DUSP14, DUSP2, DYM, DYNLL1, DYSF, E2F1, E2F2, E2F3, EAF2, EBI3, ECHDC1, ECM1, EDN1, EDN2, EDN3, EDNRA, EDNRB, EEF1A1, EEF1E1, EEF1G, EEF2, EFEMP2, EFNB1, EFS, EGF, EGFL8, EGFR, EGLN1, EGLN2, EGLN3, EGR1, EGR2, EHD4, EHMT2, EIF1B, EIF2AK1, EIF2AK2, EIF2AK3, EIF2AK4, EIF3E, ELANE, ELAVL1, ELF1, ELF3, ELF4, ELK1, ELMO1, ELN, ELOVL3, ENG, ENO1, ENPP2, ENPP3, ENTPD1, ENTPD4, ENTPD5, ENTPD7, EOMES, EP300, EPAS1, EPHA2, EPHA4, EPHB6, EPHX2, EPO, EPOR, EPS8, EPX, ERAP1, ERBB2, ERBB3, ERCC6, EREG, ERH, ERN1, ERN2, ERRFI1, ESR1, ESR2, ETS1, EVC, EYA4, F10, F11, F11R, F12, F13A1, F2, F2R, F2RL1, F2RL3, F3, F5, F7, F8, FAAH, FABP1, FABP4, FABP5, FADS2, FAF1, FAH, FAIM, FAN1, FANCA, FANCC, FANCD2, FAS, FASLG, FASN, FAU, FBXL7, FBXO32, FCAR, FCER1A, FCER1G, FCER2, FCGBP, FCGR2A, FCGR2B, FCGRT, FCN1, FDFT1, FDPS, FEM1A, FEM1B, FEN1, FER, FERMT1, FERMT3, FES, FGA, FGB, FGF1, FGF10, FGF2, FGFR1, FGFR1OP, FGFR2, FGFR3, FGG, FGL2, FGR, FKBP1A, FKBP4, FKBP5, FKBP8, FKTN, Flg, FLNA, FLOT1, FLT1, FLT3, FLT3LG, FLT4, FMN2, FMOD, FN1, FNDC4, FOS, FOSB, FOXA2, FOXD1, FOXF1, FOXF2, FOXJ1, FOXO1, FOXO3, FOXP1, FOXP3, FPGS, FPR1, FPR2, FRMD4B, FRRS1, FRY, FRZB, FSTL1, FTH1, FTL, FTO, FUBP1, FUK, FURIN, FUT7, FXR1, FYN, FZD5, G0S2, G3BP1, G6PC, G6PC3, GAA, GABBR1, GABBR2, GABRA1, GABRA2, GABRA3, GABRA4, GABRA5, GABRA6, GABRB1, GABRB2, GABRB3, GABRD, GABRG1, GABRG2, GABRG3, GABRP, GABRQ, GABRR1, GABRR2, GABRR3, GAD2, GADD45A, GADD45B, GADD45G, GAL, GALNT1, GALNT2, GALP, GAPDH, GAS6, GAST, GATA3, GATA5, GBA, GBF1, GBP2, GBP6, GC, GCA, GCNT1, GCNT3, GCSH, GDF15, GDF5, GDNF, GFAP, GFI1, GGPS1, GGT1, GGT5, GHR, GHRH, GHRHR, GHRL, GHSR, GINS2, GIPR, GIT1, GIT2, GJA1, GJB1, GJB6, GJC2, GLG1, GLI1, GLI2, GLIPR2, GLIS2, GLP1R, GLRB, GLRX5, GLUL, GNA11, GNA12, GNA13, GNAI2, GNAI3, GNAO1, GNAS, GNAZ, GNB1L, GNB2, GNL1, GNLY, GNMT, GNPAT, GNRH1, GNRH2, GNRHR, GOLGA1, GOLPH3, GP1BA, GPANK1, GPC3, GPC4, GPD1, GPI, GPR132, GPR15, GPR174, GPR18, GPR183, GPR65, GPR68, GPRC5B, GPSM1, GPSM3, GPT, GPX1, GPX2, GPX4, GPX7, GPX8, GRAMD1A, GRB10, GRB7, GREM1, GRIA2, GRIA3, GRIK4, GRIN1, GRIN2A, GRIN2B, GRIN2C, GRIN2D, GRIN3A, GRIN3B, GRK6, GRM4, GRN, GRP, GSDMC, GSK3B, GSN, GSR, GSS, GSTK1, GSTO1, GSTP1, GSTZ1, GTF2H4, GTF3C1, GTPBP1, GUCY1A2, GUCY1A3, GUCY1B3, GUCY2C, GUSB, GZMA, Hamp, HARS, Havcr1, HAVCR2, HBB, HBEGF, HCAR2, HCK, HCLS1, HCST, HDAC1, HDAC2, HDAC3, HDAC4, HDAC5, HDAC6, HDAC7, HDAC9, HDC, HEBP1, HELB, HES1, HEXA, HEXB, HGF, HIF1A, HIST1H2AC, HIST1H2BA, HLA-A, HLA-DMA, HLA-DMB, HLA-DOA, HLA-DOB, HLA-DQA1, HLA-DQB1, HLA-DRA, HLA-DRB5, HLA-E, HLA-G, HLTF, HMGB1, HMGCR, HMGCS2, HMMR, HMOX1, HMOX2, HNF4A, HNMT, HNRNPA0, HNRNPAB, HNRNPR, HOXA5, HOXA9, HOXB5, HOXC4, HOXC5, HP, HPGD, HPGDS, HPRT1, HPSE, HPX, Hrg, HRH1, HRH2, HRH3, HRH4, HS6ST1, HSD11B1, HSD17B8, HSF1, HSF2, HSP90AA1, HSP90AB1, HSP90B1, HSPA1L, HSPA5, HSPA8, HSPB1, HSPB8, HSPD1, HSPG2, HTR1A, HTR2A, HTR2B, HTR2C, HTR3B, HTR7, HTRA1, HTT, HYAL1, HYAL2, HYAL3, HYAL4, ICAM1, ICOS, ID1, ID2, ID3, IDE, IDI1, IDO1, IER3, IFI30, IFIH1, IFNA10, IFNA14, IFNA16, IFNA2, IFNA21, IFNA4, IFNA6, IFNA7, IFNA8, IFNAR1, IFNAR2, IFNB1, IFNE, IFNG, IFNGR1, IFNGR2, IFNK, IFNW1, IGF1, IGF1R, IGF2, IGFBP1, IGFBP3, IGFBP4, IGFBP5, IGFBP7, IGSF5, IKBKB, IKBKE, IKBKG, IKZF1, IKZF3, IKZF4, IL10, IL10RA, IL10RB, IL11, IL11RA, IL12A, IL12B, IL12RB1, IL12RB2, IL13, IL13RA2, IL15, IL15RA, IL16, IL17A, IL17B, IL17C, IL17D, IL17F, IL17RA, IL17RB, IL17RC, IL17RD, IL18, IL18BP, IL18R1, IL18RAP, IL19, IL1A, IL1B, IL1R1, IL1R2, IL1RL1, IL1RL2, IL1RN, IL2, IL20, IL20RA, IL21, IL21R, IL22, IL22RA2, IL23A, IL23R, IL24, IL25, IL26, IL27, IL27RA, IL2RA, IL2RB, IL2RG, Il3, Il31, IL31RA, IL33, IL34, IL36A, IL36RN, IL37, IL3RA, IL4, IL4R, IL5, IL5RA, IL6, IL6R, IL6ST, IL7, IL7R, IL9, IL9R, IMPDH1, IMPDH2, INHA, INHBA, INPP5A, INPP5D, INS, INSIG1, INSL3, INSR, IP6K3, IRAK1, IRAK3, IRAK4, IRF1, IRF2, IRF3, IRF4, IRF5, IRF6, IRF9, ISG15, ISL1, ITCH, ITFG1, ITGA1, ITGA2, ITGA4, ITGA5, ITGA6, ITGA7, ITGA9, ITGAE, ITGAL, ITGAM, ITGAV, ITGAX, ITGB1, ITGB2, ITGB3, ITGB6, ITGB7, ITGB8, ITK, ITM2B, ITPR1, ITPR3, JAG1, JAK1, JAK2, JAK3, JAM3, JMJD1C, JMJD6, JUN, JUNB, JUP, KARS, KAZN, KCNA1, KCNA3, KCNAB3, KCNE3, KCNH7, KCNJ15, KCNK2, KCNN3, KCNN4, KCTD20, KDR, KEAP1, KEL, KERA, KHSRP, KIF5A, KIT, KITLG, KL, KLC1, KLF12, KLF13, KLF2, KLF3, KLF4, KLK10, KLK13, KLK14, KLK4, KLK5, KLK6, KLK7, KLK8, KLKB1, KLRB1, KLRC1, KLRG1, KMO, KNG1, KRAS, KRT1, KRT14, KRT15, KRT16, KRT17, KRT18, KRT5, KRT8, KSR1, L1CAM, L3MBTL4, LAMA2, LAMA5, LAMB2, LANCL1, LAP3, LAT, LATS2, LBP, LCK, LCN2, LCP1, LCP2, LDHB, LDLR, LDLRAD3, LECT2, LEMD2, LEP, LEPR, LGALS1, LGALS3, LGALS3BP, LGALS4, LGALS9, LGI1, LGMN, LHCGR, LIAS, LIF, LILRA2, LILRB3, LILRB4, LIMK1, LIPA, LIPE, LIPF, LITAF, LMO4, LOR, LOXL2, LOXL3, LPA, LPAR2, LPIN1, LPL, LRFN1, LRIG2, LRP1, LRP1B, LRP2, LRP5, LRP6, LRRK2, LSP1, LSR, LTA, LTA4H, LTB, LTB4R, LTB4R2, LTBP1, LTBP3, LTBR, LTC4S, LTF, LUM, LY6E, LY6G5C, LY6G6C, LY6G6D, LY75, LY96, LYN, LYST, MAD2L2, Madcam1, MAF, MAFB, MAGI1, MAGI2, MAGI3, MALT1, MAML1, MAML3, MAN2A1, MAOA, MAOB, MAP1LC3B, MAP2K1, MAP2K2, MAP2K3, MAP2K5, MAP3K1, MAP3K14, MAP3K2, MAP3K3, MAP3K5, Map3k7, MAP3K8, MAP4K1, MAP4K4, MAPK11, MAPK14, MAPK3, MAPK7, MAPK8, MAPK9, MAPKAPK2, MAPKAPK3, MAPRE1, MAPT, MARCO, MARK2, MAS1, MAT1A, MATN3, MAVS, MAZ, MB21D1, MBD1, MBD2, MBL2, MBP, MC2R, MC3R, MCAM, MCL1, MCM5, MCPH1, MCTP2, MDC1, MDK, MDM2, MECOM, MEFV, MEN1, MEP1A, MEP1B, MERTK, MESP2, METRNL, METTL9, MFGE8, MFHAS1, MFSD2A, MGAM, MGAT2, MGAT5, MGAT5B, MGLL, MICA, MIF, MINK1, MIPEP, MIR17HG, MKI67, MLLT6, MLPH, MME, MMEL1, MMP1, MMP10, MMP11, MMP12, MMP13, MMP14, MMP15, MMP16, MMP17, MMP19, MMP2, MMP21, MMP24, MMP25, MMP26, MMP27, MMP28, MMP3, MMP7, MMP8, MMP9, MNAT1, MNT, MNX1, MOB3B, MOG, MOK, MOSPD2, MPL, MPO, MPP1, MR1, MRC1, MRFAP1, MRPS15, MRPS28, MRPS36, MRS2, MS4A1, MS4A2, MS4A6A, MS4A7, MSH2, MSH5, MSN, MSR1, MSRA, MST1, MST1R, MTA2, MTAP, MTDH, MTHFD2L, MTHFR, MTOR, MTR, MTTP, MUC1, Muc19, MUC2, MUC5AC, MUC5B, MUS81, MUT, MVK, MXI1, MYBPC1, MYBPC2, MYCBP2, MYD88, MYH10, MYH6, MYH9, MYL12A, MYL9, MYLK, MYO18A, MYO1C, MYO1F, MYO9B, NAA16, NAA25, NAAA, NAGK, NAGLU, NAIP, NAMPT, NAPA, NAV3, NBN, NBR1, NCF1, NCF4, NCKAP1L, NCL, NCOA2, NCOA5, NCSTN, NDFIP1, NDRG2, NDST2, NDUFB10, NEDD4L, NEDD9, NEIL1, NEIL2, NELL1, NEO1, NFAT5, NFATC1, NFATC2, NFATC3, NFATC4, NFE2L1, NFE2L2, NFIL3, NFIX, NFKB1, NFKB2, NFKBIA, NFKBIB, NFKBID, NFKBIL1, NFKBIZ, NFRKB, NFX1, NGF, NGFR, NINJ1, NKX2-3, NKX2-5, NKX3-2, NLRC3, NLRC4, NLRP10, NLRP12, NLRP3, NLRP6, NLRX1, NMI, NMT2, NMU, NOD1, NOD2, NODAL, NOM1, NONO, NOS1, NOS2, NOS3, NOTCH1, NOTCH2, NOTCH3, NOTCH4, NOV, NOX1, NOX4, NPC1, NPC1L1, NPPB, NPR1, NPS, NPW, NPY, NPY1R, NQO1, NQO2, NR0B2, NR1D1, NR1D2, NR1H2, NR1H3, NR1H4, NR1I2, NR2C1, NR2C2, NR2F1, NR3C1, NR3C2, NR4A1, NR4A2, NR4A3, NR5A2, NRG1, NRG4, NRM, NRP1, NRP2, NRSN2, NRTN, NT5E, NTAN1, NTF3, NTF4, NTN1, NTRK1, NTRK2, NTS, NUDCD2, NUMB, NUP62, NUP85, NUPR1, OCA2, OCLN, ODAM, ODC1, OFD1, OGG1, OLAH, OLFM4, OLR1, OMA1, OPRD1, OPRK1, OPRM1, OPTN, OR12D3, ORAI3, ORM1, ORMDL3, OSM, OSMR, OSTM1, OTUD7B, P2RX2, P2RX3, P2RX7, P2RY12, P2RY13, P2RY2, P2RY6, P4HA2, P4HB, PADI2, PADI4, PANK2, PANX3, PARD3B, PARK7, PARP1, PARP2, PARP3, PARP4, PAX1, PAX5, PAX6, PBK, PBLD, PBX2, PCDH15, PCDHGB4, PCM1, PDCD1, PDCD1LG2, PDCD4, PDE11A, PDE2A, PDE3A, PDE3B, PDE4A, PDE4B, PDE4C, Pde4d, PDE5A, PDE6B, PDE7A, PDE7B, PDE8A, PDGFB, PDGFD, PDGFRA, PDGFRB, PDGFRL, PDIA3, PDK2, PDK4, PDLIM1, PDX1, PDYN, PECAM1, PELI1, PELI3, PEMT, PENK, PER1, PF4, PF4V1, PFAS, PFKFB3, PFN1, PGAP3, PGF, PGK1, PGLYRP1, PGLYRP2, PGLYRP3, PGLYRP4, PGM3, PGR, PHACTR3, PHB, PHB2, PHC1, PHF1, PHF19, PHF20, PHGDH, PHIP, PHLPP1, PHLPP2, PHTF1, PHYH, PI4KA, PIAS3, PIGR, PIK3AP1, PIK3C3, PIK3CA, PIK3CB, PIK3CD, PIK3CG, PIK3R1, PIK3R5, PILRA, PIM1, PIM2, PIP, PIP5K1C, PITPNB, PKD1, PKN1, PKP3, PLA2G10, PLA2G1B, PLA2G2A, PLA2G2D, PLA2G2E, PLA2G2F, PLA2G3, PLA2G4A, PLA2G5, PLA2G6, PLA2G7, PLA2R1, PLAA, PLAT, PLAU, PLAUR, PLCB3, PLCE1, PLCG2, PLD1, PLEC, PLEKHA1, PLEKHA2, PLEKHA7, PLG, PLIN1, PLIN2, PLP1, PLS1, Pmaip1, PNKD, PNLIP, PNLIPRP1, PNMA5, PNOC, PNPLA3, POLA1, POLB, POLD1, POLE, POLE2, POLE3, POLE4, POLG, POLR2L, POMC, PON1, PON2, PON3, POR, POSTN, POU4F1, PPARA, PPARD, PPARG, PPARGC1A, PPAT, PPBP, PPFIA1, PPIA, PPIB, PPID, PPIL6, PPM1A, PPM1D, PPM1H, PPP1R11, PPP1R7, PPP2CA, PPP2R2B, PPP3CA, PPP3CB, PPP3CC, PPP3R1, PPP3R2, PPT1, PPT2, PPTC7, PRAP1, PRDM1, PRDX1, PRDX2, PRDX5, PRDX6, PREP, PREX1, PRF1, PRG2, PRG3, PRKAA1, PRKAR1B, PRKCA, PRKCB, PRKCD, PRKCE, PRKCG, PRKCQ, PRKCZ, PRKD1, PRKG1, PRMT1, PRMT2, PRMT3, PRNP, PROC, PROCR, PROM1, PROS1, PRR5, PRRC2A, PRSS1, PRSS2, PRSS3, PRTN3, PRUNE2, PSAP, PSEN1, PSEN2, PSENEN, PSMB1, PSMB2, PSMB5, PSMB8, PSMB9, PSMD1, PSMD2, PSTPIP1, PSTPIP2, PTAFR, PTEN, PTGDR, PTGDR2, PTGDS, PTGER1, PTGER2, PTGER3, PTGER4, PTGES, PTGIR, PTGIS, PTGS1, PTGS2, PTH, PTH1R, PTH2R, PTK2B, PTN, PTPN1, PTPN11, PTPN2, PTPN22, PTPN6, PTPRC, PTPRE, PTPRJ, PTPRN, PTPRN2, PTPRO, PTPRZ1, PTTG1, PTX3, PURA, PXK, PXN, PYCARD, PYDC2, Pzp, QKI, R3HDM2, RAB10, RAB11B, RAB1B, RAB27A, RAB2A, RAB34, RABGAP1, RABGEF1, RAC1, RAC2, RAC3, RAG1, RAG2, RALA, RALB, RALGDS, RAMP2, RAP1GAP, RAPGEF3, RAPH1, RARA, RARB, RARG, RARRES2, RASGRF1, RASGRP4, RASSF1, RASSF5, RASSF8, RB1, RBCK1, RBL1, RBM38, RBP4, RBP7, RBPJ, RC3H1, RCSD1, RDH8, RECK, REG1A, REG1B, REG3A, REL, RELA, RELB, REN, RENBP, RET, RETN, RETNLB, RFTN1, RFX3, RGL2, RGS1, RGS16, RGS6, RHBDD3, RHOA, RHOB, RHOBTB3, RICTOR, RIMS1, RIPK1, RIPK2, RIPK3, RIPK4, RNASE2, RNASEL, RNF128, RNF138, RNF139, RNF14, RNF149, RNF169, RNF39, RNF5, ROBO1, ROCK1, RORA, RORC, RPL11, RPL13A, RPL15, RPL18A, RPL19, RPL31, RPL32, RPS13, RPS16, RPS18, RPS19, RPS24, RPS3, RPS6KA4, RPS6KA5, RPSA, RPTOR, RRAS, RRM1, RRM2, RRN3, RSBN1, RTEL1, RUNX3, RUVBL1, RUVBL2, RXRA, RXRB, RXRG, RYR2, S100A10, S100A11, S100A12, S100A14, S100A4, S100A7, S100A7A, S100A8, S100A9, S100B, S100PBP, S1PR1, S1PR2, S1PR3, S1PR4, SAA1, SAFB, SALL4, SAMD9L, SASH3, SAT1, SBDS, SBNO2, SCAP, SCARA5, SCARB1, SCARF1, SCD, SCG2, SCG5, SCGB1A1, SCN10A, SCN11A, SCN1A, SCN1B, SCN2A, SCN2B, SCN3A, SCN3B, SCN4A, SCN4B, SCN5A, SCN7A, SCN8A, SCN9A, SCP2, SCT, SCTR, SCYL1, SDC1, SDC2, SDC3, SDC4, SEC14L3, SEC24B, SEC61A1, SEC62, SEL1L, SELE, SELENBP1, SELL, SELP, SELPLG, SEMA4A, SEMA4D, SEMA7A, SERINC2, SERINC3, SERPINA1, SERPINA3, SERPINB1, SERPINB13, SERPINB2, SERPINB3, SERPINB4, SERPINB5, SERPINC1, SERPINE1, SERPINF1, SERPING1, SETD6, SFN, SFRP1, SFRP5, SFTPA1, SFTPC, SFTPD, SGCA, SGCB, SGK1, SGMS1, SGPL1, SH2D1A, SH2D2A, SH3BP2, SH3BP4, SHARPIN, SHC1, SHCBP1, SHH, SHPK, SIAE, SIGIRR, SIGLEC10, SIGLEC8, SIGLEC9, SIGMAR1, SIL1, SIPA1, SIRPA, SIRT1, SIRT3, SIRT6, SIT1, SKIV2L, SLAMF1, SLAMF8, SLC10A1, SLC11A1, SLC15A1, SLC17A3, SLC18A1, SLC18A2, SLC19A1, SLC19A3, SLC1A1, SLC1A2, SLC1A3, SLC1A4, SLC1A5, SLC1A6, SLC1A7, SLC22A1, SLC22A12, SLC22A4, SLC22A6, SLC22A8, SLC25A32, SLC26A1, SLC2A4, SLC2A9, SLC33A1, SLC37A4, SLC39A4, SLC39A5, SLC39A8, SLC44A4, SLC5A2, SLC6A2, SLC6A3, SLC6A4, SLC7A1, SLC7A11, SLC7A2, SLC7A9, SLC8A1, SLC9A1, SLC9A2, SLC9A4, SLFN12L, SLFN5, SLIT2, SLPI, SMAD1, SMAD2, SMAD3, SMAD4, SMAD7, SMARCA4, SMG7, SMPD1, SMPDL3B, SMTN, SMURF2, SNAI2, SNAP25, SNCA, SNCAIP, SND1, SNRNP200, SOAT1, SOCS1, SOCS2, SOCS3, SOCS5, SOD1, SOD2, SOD3, SORL1, SOX18, SOX5, SPACA3, SPAG1, SPAG16, SPARC, SPATS2L, SPDEF, SPHK1, SPHK2, SPI1, SPINK1, SPINK5, SPN, SPOCK1, SPON2, SPP1, SPRY1, SPRY2, SPTA1, SQLE, SQSTM1, SRC, SRCIN1, SRD5A2, SREBF2, SRP14, SSBP2, SST, ST14, ST3GAL3, ST6GAL1, ST6GALNAC4, STAB1, STAC, STAP1, STAP2, STARD7, STAT1, STAT3, STAT4, STAT5A, STAT5B, STAT6, STEAP2, STEAP4, STIM1, STIM2, STIP1, STK17B, STK19, STK25, STK39, STRA6, STUB1, STX2, SUCNR1, SUMO1, SUMO3, SWAP70, SWT1, SYCN, SYK, SYN3, SYNE1, SYNE2, SYT3, SYT6, SYT7, SYT9, SYVN1, TAAR1, TAC1, TACR1, TAF13, TAF7L, TAGAP, TALDO1, TANK, TAP2, TAPBP, TAX1BP1, TBC1D23, TBCD, TBCE, TBK1, TBRG1, TBX21, TBXA2R, TBXAS1, TCF4, Tcf7, TCF7L2, TCIRG1, TEK, TERC, TERT, TF, TFF1, TFF2, TFF3, TFG, TFPI, TFPI2, TFRC, TGFA, TGFB1, TGFB2, TGFBR1, TGFBR2, TGM2, TGM3, TGM6, TH, THBD, THBS1, THBS2, THBS4, THOP1, TIA1, TIAM1, TICAM1, TIE1, TIGIT, TIMD4, TIMP1, TIMP2, TIMP3, TIMP4, TIRAP, TJP1, TK1, TK2, TKT, TKTL1, TLE3, TLR1, TLR10, TLR2, TLR3, TLR4, TLR5, TLR6, TLR7, TLR8, TLR9, TMEFF2, TMEM102, TMEM127, TMEM135, TMEM173, TMEM51, TMF1, TMX4, TNC, TNF, TNFAIP3, TNFAIP6, TNFAIP8, TNFAIP8L2, TNFRSF10A, TNFRSF11A, TNFRSF11B, TNFRSF12A, TNFRSF13B, TNFRSF14, TNFRSF18, TNFRSF1A, TNFRSF1B, TNFRSF21, TNFRSF25, TNFRSF4, TNFRSF6B, TNFRSF8, TNFRSF9, TNFSF10, TNFSF11, TNFSF12, TNFSF13, TNFSF13B, TNFSF14, TNFSF15, TNFSF18, TNFSF4, TNFSF8, Tnfsf9, TNIP1, TNIP2, TNNC1, TNNC2, TNNI3, TNRC6B, TNS1, TNXB, TOB1, TOLLIP, TOP2A, TOP2B, TOP3B, TOPBP1, TP53, TP53BP1, TP53BP2, TP53INP1, TP63, TP73, TPI1, TPM3, TPMT, TPSG1, TPST1, TRADD, TRAF1, TRAF2, TRAF3, TRAF3IP2, TRAF4, TRAF6, TRAK2, TRAM2, TRAPPC2L, TREM1, TREM2, TREML2, TREX1, TRIM10, TRIM15, TRIM21, TRIM26, TRIM28, TRIM31, TRIM35, TRIM40, TRIO, TRIOBP, TRNT1, TROVE2, TRPA1, TRPC6, TRPM2, TRPM4, TRPM8, TRPV1, TRPV2, TRPV3, TRPV4, TSC1, TSLP, TSPAN2, TSPO, TSTA3, TTC39C, TUBA1A, TUBA1C, TUBA4A, TUBA8, TUBB, TUBB1, TUBB2A, TUBB3, TUBB4A, TUBB4B, TUBD1, TUBE1, TUBG1, TUBG2, TUSC2, TWIST1, TWSG1, TXK, TXN, TYK2, TYMP, TYMS, TYR, TYRO3, TYROBP, TYRP1, UACA, UBAC1, UBASH3A, UBASH3B, UBE2G2, UBE2H, UBE2I, UBE2L6, UBE2N, UCHL1, UCN, UFL1, ULBP1, UMOD, UNC13D, UNC93A, UNC93B1, Uox, UPP1, UQCRC2, USP11, USP15, USP18, USP7, UTRN, UTS2, UVRAG, VARS, VARS2, VASH1, VAV1, VAV3, VCAM1, VCL, VCP, VDAC2, VDR, VEGFA, VEGFB, VEGFC, VHL, VIM, VIP, VIPR1, VIPR2, VNN1, VPS35, VPS37C, VPS52, VPS53, VRK1, VSIG10, VSIG4, VTCN1, VTN, VWF, WAS, WDR46, WFDC1, WHAMM, WIPF1, WNK1, WNT1, WNT10A, WNT10B, WNT11, WNT3, WNT3A, WNT4, WNT5A, WNT5B, WNT7B, WNT9A, WNT9B, WRN, WT1, WWTR1, XBP1, XCL1, XCR1, XDH, XIAP, XPA, XRCC6, YAP1, YARS, YBX1, YIPF6, YTHDC2, ZAP70, ZBP1, ZBTB12, ZBTB16, ZBTB22, ZBTB9, ZC3H12A, ZC3H12D, ZC3HAV1L, ZCCHC6, ZCRB1, ZFP36, ZMYND11, ZMYND8, ZNF143, ZNF148, ZNF280B, ZNF281, ZNF326, ZNF395, ZNF546, ZNF580, ZNF7, ZNF784, ZP3, ZYX |
| **Mitochondria genes (n=1532)** | |
|  | AADAT, AARS2, AASS, ABAT, ABCA13, ABCA9, ABCB10, ABCB6, ABCB7, ABCB8, ABCB9, ABCD1, ABCD2, ABCD3, ABCE1, ABCF2, ABCG1, ABCG2, ABHD10, ABHD11, ABHD6, ABL1, ACAA1, ACAA2, ACACA, ACACB, ACAD10, ACAD11, ACAD8, ACAD9, ACADL, ACADM, ACADS, ACADSB, ACADVL, ACAT1, ACBD3, ACCS, ACLY, ACN9, ACO1, ACO2, ACOT2, ACOT7, ACOT8, ACOT9, ACOX1, ACOX3, ACP6, ACSBG2, ACSF2, ACSF3, ACSL1, ACSL4, ACSL5, ACSL6, ACSM1, ACSM2A, ACSM2B, ACSM3, ACSM4, ACSM6, ACSS1, ACSS3, ACYP2, ADCK1, ADCK2, ADCK3, ADCK4, ADCK5, ADCY10, ADH5, ADHFE1, ADO, ADPRHL2, ADSL, AFF4, AFG3L2, AGAP2, AGK, AGMAT, AGPAT5, AGPS, AGR2, AGTPBP1, AGXT, AGXT2, AHCYL1, AIFM1, AIFM2, AIFM3, AK2, AK3, AK4, AKAP1, AKAP10, AKAP8, AKR1B10, AKR7A2, AKT1, ALAS1, ALAS2, ALDH18A1, ALDH1B1, ALDH1L1, ALDH1L2, ALDH2, ALDH3A2, ALDH4A1, ALDH5A1, ALDH6A1, ALDH7A1, ALDH9A1, ALDOC, ALKBH1, ALKBH3, ALS2CR3, AMACR, AMT, ANGEL2, ANXA10, AP2M1, APEX1, APEX2, APOA1BP, APOO, APOOL, APOPT1, ARAF, ARF5, ARG2, ARGLU1, ARL2, ARMC1, ARMC10, ARMS2, ARSB, AS3MT, ASAH2, ASB9, ATAD1, ATAD3A, ATAD3B, ATCAY, ATG4D, ATIC, ATP10D, ATP5A1, ATP5B, ATP5C1, ATP5D, ATP5EP2, ATP5F1, ATP5G1, ATP5G2, ATP5G3, ATP5H, ATP5I, ATP5J, ATP5J2-PTCD1, ATP5L, ATP5O, ATP5S, ATP5SL, ATP6, ATP6V1A, ATP6V1E1, ATP7B, ATP8, ATPAF1, ATPAF2, ATPIF1, ATRX, ATXN2, AUH, AZIN2, BAD, BAG5, BAK1, BARX2, BAX, BBC3, BBOX1, BCAT1, BCAT2, BCKDHA, BCKDHB, BCKDK, BCL2, BCL2L1, BCL2L10, BCL2L11, BCL2L13, BCL2L2, BCO2, BCS1L, BDH1, BDH2, BFSP1, BID, BLID, BLOC1S1, BLOC1S2, BNIP1, BNIP3, BNIP3L, BOK, BOLA1, BOP, BPHL, BRAF, BRD8, BRINP3, BSG, BZRAP1, C10orf10, C10orf2, C12orf10, C12orf65, C14ORF119, C14orf159, C14orf2, C15orf40, C15orf48, C15orf61, C15ORF62, C16orf91, C17orf89, C19ORF12, C19orf52, C19orf70, C1QBP, C20orf24, C21orf33, C2orf47, C2orf69, C3orf33, C5orf63, C6orf136, C6orf203, C6orf57, C7orf55, C8orf82, C9ORF89, CA5A, CA5B, CA5BP1, CAMK2A, CAPN10, CAPRIN2, CARKD, CARS2, CASP2, CASP4, CASP8, CASP8AP2, CASP9, CASQ1, CAT, CBR3, CBR4, CCBL2, CCDC109B, CCDC127, CCDC136, CCDC19, CCDC51, CCDC58, CCDC90B, CCT7, CD3EAP, CDC25C, CDK1, CDK7, CDKN2A, CECR5, CEP89, CERK, CHAT, CHCHD1, CHCHD10, CHCHD2P9, CHCHD3, CHCHD4, CHCHD5, CHCHD6, CHCHD7, CHDH, CHMP2B, CHPF, CHPT1, CIAPIN1, CIDEA, CISD1, CISD2, CISD3, CKB, CKMT1B, CKMT2, CLIC1, CLIC4, CLN3, CLN8, CLPB, CLPP, CLPX, CLTC, CLU, CLYBL, CMC4, COA4, COA7, COASY, COL4A3BP, COMT, COMTD1, COQ10A, COQ10B, COQ2, COQ3, COQ4, COQ5, COQ6, COQ7, COQ9, COX1, COX10, COX14, COX15, COX16, COX17, COX18, COX19, COX20, COX3, COX4I1, COX4I2, COX5A, COX5B, COX6A1, COX6A2, COX6B1, COX6B2, COX6C, COX7A1, COX7A2, COX7A2L, COX7B, COX7C, COX8A, COX8C, CPOX, CPS1, CPT1A, CPT1B, CPT1C, CPT2, CRAT, CRLS1, CROT, CRY1, CRY2, CRYAB, CRYM, CRYZ, CS, CTPS2, CTSA, CTSB, CTSD, CTU1, CXORF23, CYB5A, CYB5B, CYB5R1, CYB5R2, CYB5R3, CYBA, CYBB, CYC1, CYCS, CYP11A1, CYP11B1, CYP11B2, CYP17A1, CYP1A1, CYP1B1, CYP24A1, CYP27A1, CYP27B1, CYP2D6, CYP2E1, CYTB, D2HGDH, DACT2, DAOA, DAP3, DARS2, DBI, DBT, DCAF5, DCAKD, DCPS, DCXR, DDAH1, DDAH2, DDHD2, DDIT4, DDX23, DDX28, DECR1, DEPP, DGAT2, DGUOK, DHCR24, DHFRL1, DHODH, DHRS1, DHRS2, DHRS4, DHRS7B, DHRSX, DHTKD1, DHX29, DHX30, DHX32, DIABLO, DISC1, DLAT, DLD, DLST, DMGDH, DMPK, DNA2, DNAJA1, DNAJA3, DNAJC11, DNAJC15, DNAJC19, DNAJC27, DNAJC28, DNAJC30, DNAJC4, DNAJC5, DNLZ, DNM1L, DNM3, DPYSL2, DRG2, DSP, DUS2, DUSP26, DUT, E9PI62, EARS2, ECH1, ECHDC1, ECHDC2, ECHDC3, ECHS1, ECI1, ECI2, ECSIT, EEFSEC, EFHD1, EHHADH, ELAC2, ELK3, EMC8, ENDOG, ENOSF1, EPHX2, ERAL1, ERCC6L2, ERN1, ESR2, ETFA, ETFB, ETFDH, ETNPPL, EXOG, F5H5T6, FABP1, FADS1, FAHD1, FAHD2A, FAM136A, FAM162A, FAM185A, FAM213A, FAM32A, FAM65B, FAM72A, FANCG, FARS2, FASN, FASTK, FASTKD1, FASTKD2, FASTKD3, FASTKD5, FBXL4, FBXO7, FDPS, FDX1, FDX1L, FDXR, FECH, FEN1, FH, FHIT, FIBP, FIS1, FITM2, FKBP10, FKBP4, FKBP8, FLAD1, FLVCR1, FOXO1, FOXRED1, FPGS, FSIP2, FTH1, FTMT, FTSJ2, FUNDC1, FUNDC2, FXN, FYN, G0S2, GADD45GIP1, GAPDH, GARS, GATC, GATM, GBAS, GBF1, GCAT, GCDH, GCK, GCKR, GCSH, GDF5OS, GFER, GFM1, GFM2, GHITM, GHR, GIMAP8, GIT1, GK, GLDC, GLOD4, GLRX, GLRX2, GLRX5, GLS, GLS2, GLUD1, GLUD2, GLUL, GLYAT, GLYATL1, GLYATL2, GLYATL3, GLYCTK, GM2A, GML, GMPPB, GNB2L1, GNG5, GNL3L, GNPAT, GOLPH3, GOT2, GPAM, GPAT2, GPD1, GPD2, GPI, GPRC5C, GPT2, GPX1, GPX4, GRAMD4, GRHPR, GRN, GRPEL1, GRSF1, GSDMC, GSR, GSTK1, GSTO1, GSTP1, GSTZ1, GTPBP10, GTPBP3, GTPBP6, GTPBP8, GUF1, GUK1, H6PD, HADH, HADHA, HADHB, HAGH, HAO2, HAP1, HARS2, HAX1, HCCS, HCFC1, HCLS1, HDDC2, HDHD3, HEATR1, HEBP1, HEBP2, HEMK1, HIBADH, HIBCH, HIGD1A, HIGD2A, HINT1, HINT2, HINT3, HIVEP1, HK1, HK2, HK3, HLCS, HMBS, HMGCL, HMGCS2, HOGA1, HOXB9, HRK, HRSP12, HSCB, HSD17B10, HSD17B4, HSD17B8, HSDL1, HSDL2, HSH2D, HSP90AA1, HSP90AB1, HSPA1A, HSPA2, HSPA4, HSPA9, HSPB7, HSPD1, HSPE1, HTATIP2, HTRA2, HTT, IARS2, IBA57, ICT1, IDE, IDH1, IDH2, IDH3A, IDH3B, IDH3G, IDI1, IFI27, IFI6, IFIT3, ILF3, IMMP1L, IMMP2L, IMMT, IQCE, IREB2, IRP1, ISCA1, ISCA2, ISCU, ISOC2, IVD, JTB, KANK2, KARS, KCNJ11, KCNJ8, KIAA0100, KIAA0141, KIAA0391, KIAA1279, KIAA1683, KIF1B, KLK6, KMO, KRAS, KRT5, KYNU, L2HGDH, LACE1, LACTB, LACTB2, LAMC1, LAP3, LARS2, LBR, LDHA, LDHAL6B, LDHB, LDHD, LEPR, LETM1, LETM2, LETMD1, LIAS, LIG1, LIG3, LIPF, LIPT1, LIPT2, LONP1, LONP2, LRP5, LRPPRC, LRRC75A-AS1, LRRK1, LRRK2, LYPLA1, LYPLAL1, LYRM1, LYRM2, LYRM4, LYRM5, MAATS1, MALSU1, MAOA, MAOB, MAP2K1, MAP2K2, MAPK1, MAPK10, MAPK12, MAPK14, MAPK3, MAPK8, MAPK8IP1, MAPK9, MARC1, MARC2, MARCH5, MARK2, MARS, MAT2B, MAVS, MCAT, MCCC1, MCCC2, MCCD1, MCEE, MCL1, MCUR1, MDH1, MDH2, ME1, ME2, ME3, MECR, MED7, METAP1D, METTL12, METTL15, METTL17, METTL5, METTL8, MFF, MFN1, MGME1, MGST1, MGST3, MIEF2, MINOS1-NBL1, MIPEP, MLH1, MLYCD, MMAB, MMACHC, MMADHC, MMP2, MOAP1, MOBP, MOCS1, MPC2, MPDU1, MPO, MPP7, MPST, MPV17, MPV17L, MPV17L2, MRM1, MRPL1, MRPL10, MRPL11, MRPL12, MRPL13, MRPL14, MRPL15, MRPL16, MRPL17, MRPL18, MRPL19, MRPL2, MRPL20, MRPL21, MRPL22, MRPL23, MRPL24, MRPL27, MRPL28, MRPL3, MRPL30, MRPL32, MRPL33, MRPL34, MRPL35, MRPL36, MRPL37, MRPL38, MRPL39, MRPL4, MRPL40, MRPL41, MRPL42, MRPL43, MRPL44, MRPL45, MRPL46, MRPL47, MRPL48, MRPL49, MRPL50, MRPL51, MRPL52, MRPL53, MRPL54, MRPL57, MRPL9, MRPS10, MRPS11, MRPS12, MRPS14, MRPS15, MRPS16, MRPS17, MRPS18A, MRPS18B, MRPS18C, MRPS2, MRPS21, MRPS22, MRPS23, MRPS24, MRPS25, MRPS26, MRPS27, MRPS28, MRPS30, MRPS31, MRPS33, MRPS34, MRPS35, MRPS36, MRPS5, MRPS6, MRPS7, MRPS9, MRRF, MRS2, MSRA, MSRB2, MSRB3, MTCH1, MT-CO2, MT-CYB, MTERF4, MTERFD1, MTERFD2, MTERFD3, MTFMT, MTFP1, MTFR2, MTG2, MTHFD1, MTHFD1L, MTHFD2, MTHFD2L, MTHFS, MTIF2, MTIF3, MTO1, MTPAP, MTRF1, MT-RNR2, MTUS1, MTX1, MTX2, MUL1, MUT, MUTYH, MYCBP, MYL10, MYO1C, NADK2, NAGS, NAIF1, NAPG, NARS, NARS2, NBR1, NCBP1, NCEH1, NCOA4, ND1, ND2, ND3, ND4, ND4L, ND5, ND6, NDFIP2, NDUFA1, NDUFA10, NDUFA11, NDUFA12, NDUFA13, NDUFA2, NDUFA3, NDUFA4, NDUFA5, NDUFA6, NDUFA7, NDUFA8, NDUFA9, NDUFAB1, NDUFAF1, NDUFAF2, NDUFAF3, NDUFAF7, NDUFB1, NDUFB10, NDUFB11, NDUFB2, NDUFB3, NDUFB4, NDUFB5, NDUFB6, NDUFB7, NDUFB8, NDUFB9, NDUFC1, NDUFC2, NDUFS1, NDUFS2, NDUFS3, NDUFS4, NDUFS5, NDUFS6, NDUFS7, NDUFS8, NDUFV1, NDUFV2, NDUFV3, NEFH, NEU4, NFKB1, NFS1, NFU1, NGB, NGRN, NIF3L1, NIPSNAP1, NIPSNAP3A, NIPSNAP3B, NIT1, NIT2, NLN, NLRP5, NLRX1, NME1, NME3, NME4, NMES1, NMNAT3, NNT, NOA1, NOL3, NOL6, NOP14, NOS1, NOX4, NPTX1, NRD1, NSUN3, NSUN4, NT5C3A, NT5DC2, NT5DC3, NT5M, NTHL1, NUBPL, NUCB2, NUDT1, NUDT13, NUDT19, NUDT2, NUDT5, NUDT6, NUDT8, NUDT9, NXNL1, OAS1, OAS2, OAT, OBSCN, OCIAD1, OCIAD2, OGDH, OGDHL, OGG1, OGT, OLFM4, OMA1, OPA1, OPA3, OSBPL1A, OSGEPL1, OTC, OXA1L, OXCT1, OXLD1, OXNAD1, OXR1, OXSM, P4HA1, P4HB, PABPC5, PACRG, PACS2, PACSIN2, PAICS, PAK7, PAM16, PANK2, PARG, PARK2, PARK7, PARL, PARS2, PC, PCBD2, PCCA, PCCB, PCK2, PDF, PDHA1, PDHA2, PDHB, PDHX, PDK1, PDK2, PDK3, PDK4, PDP1, PDP2, PDPR, PDSS1, PDSS2, PECR, PEMT, PET100, PET117, PEX11B, PEX5, PFDN2, PFDN4, PGAM5, PGS1, PHB, PHB2, PHYH, PHYKPL, PI4K2A, PI4KA, PICK1, PIF1, PIN4, PINK1, PISD, PITRM1, PKM, PLA2G15, PLA2G2A, PLGRKT, PLIN5, PLN, PLSCR3, PMAIP1, PMPCA, PMPCB, PNKD, PNPLA7, PNPLA8, PNPO, PNPT1, POLD3, POLDIP2, POLG, POLG2, POLRMT, PON2, POR, PPA2, PPARGC1B, PPIF, PPL, PPM1E, PPM1K, PPOX, PPP1CC, PPP2CA, PPP2R1A, PPP2R2B, PPP3CA, PPTC7, PPWD1, PRDX2, PRDX3, PRDX4, PRDX5, PRDX6, PRELID1, PRELID2, PREPL, PRKACA, PRKCA, PRKCD, PRKCE, PRODH, PRODH2, PROSC, PRR5L, PRSS35, PSAP, PSEN1, PSMA6, PSMB3, PSTK, PTCD1, PTCD2, PTCD3, PTEN, PTGES2, PTPMT1, PTPN11, PTPN4, PTRF, PTRH1, PTRH2, PTS, PUS1, PUSL1, PXMP2, PXMP4, PYCARD, PYCR1, Q6ZSR3, QIL1, QRSL1, QTRT1, QTRTD1, RAB11A, RAB11B, RAB11FIP5, RAB1B, RAB24, RAB32, RAB35, RAB3D, RAB40AL, RAB8B, RAD51, RAD51C, RAI14, RANBP2, RAP1GDS1, RARS, RARS2, RBFA, RCN2, RDH11, RDH13, RDH14, RECQL4, REEP1, REXO2, RFK, RGS2, RHBDD1, RHOA, RHOT1, RHOT2, RILP, RIPK1, RMDN3, RMND1, RNASEH1, RNASEL, RNF168, RNF5, RNMTL1, ROMO1, RPIA, RPL10A, RPL34, RPL35A, RPP14, RPS14, RPS15A, RPS18, RPS6KB1, RPUSD3, RPUSD4, RRP15, RSAD1, RSAD2, RTN4IP1, SACS, SAMM50, SARDH, SARM1, SARS, SARS2, SCCPDH, SCO1, SCO2, SCP2, SDHA, SDHAF1, SDHAF2, SDHB, SDHC, SDHD, SDR39U1, SDS, SDSL, SECISBP2, SELO, SERAC1, SERHL2, SETD9, SFXN1, SFXN2, SFXN3, SFXN4, SFXN5, SGK1, SH3BP5, SHC1, SHMT1, SHMT2, SIAH3, SIRT1, SIRT3, SIRT4, SIRT5, SIVA1, SLC16A1, SLC16A11, SLC16A7, SLC22A4, SLC25A1, SLC25A10, SLC25A11, SLC25A12, SLC25A13, SLC25A14, SLC25A15, SLC25A16, SLC25A17, SLC25A18, SLC25A19, SLC25A20, SLC25A21, SLC25A22, SLC25A23, SLC25A24, SLC25A25, SLC25A26, SLC25A27, SLC25A28, SLC25A29, SLC25A3, SLC25A30, SLC25A31, SLC25A32, SLC25A33, SLC25A34, SLC25A35, SLC25A36, SLC25A37, SLC25A38, SLC25A39, SLC25A4, SLC25A40, SLC25A41, SLC25A42, SLC25A43, SLC25A44, SLC25A45, SLC25A46, SLC25A47, SLC25A48, SLC25A53, SLC25A6, SLC27A2, SLC27A3, SLC30A6, SLC30A9, SLC35F6, SLC37A4, SLC8A3, SLC9A1, SLC9A6, SLIRP, SLIT3, SLMO1, SLMO2, SMAD1, SMIM4, SNAP29, SNCA, SNCB, SND1, SOD1, SOD2, SORD, SOX4, SPARC, SPATA19, SPATA20, SPATA5, SPG7, SPHKAP, SPR, SPRYD4, SPTLC2, SQRDL, SRC, SREK1, SRP19, SSBP1, STAP1, STAR, STARD13, STARD3, STARD7, STK11, STOM, STOML1, STOML2, STX17, STXBP1, SUCLA2, SUCLG1, SUGCT, SUOX, SUPV3L1, SURF1, SYNE2, SYNJ2BP, TACO1, TANGO2, TAP1, TARS, TARS2, TAT, TATDN3, TAZ, TBC1D15, TCAIM, TCHP, TCIRG1, TDH, TEFM, TFAM, TFB1M, TFB2M, TGM2, TH, THEM4, THG1L, THNSL1, TIMM10B, TIMM13, TIMM17A, TIMM21, TIMM22, TIMM23, TIMM44, TIMM50, TIMM8A, TIMM8B, TIMM9, TIMMDC1, TK2, TKT, TMBIM4, TMEM11, TMEM126A, TMEM126B, TMEM143, TMEM14C, TMEM160, TMEM177, TMEM186, TMEM205, TMEM70, TMEM8B, TMLHE, TMTC1, TNNC1, TOMM20, TOMM22, TOMM34, TOMM40, TOMM40L, TOMM5, TOMM6, TOMM7, TOMM70A, TOP1MT, TOP3A, TP53, TP53AIP1, TPI1, TPO, TPP1, TRAF3, TRAF6, TRAK1, TRAK2, TRAP1, TRIAP1, TRIM31, TRIM39, TRIT1, TRMT10C, TRMT11, TRMT2B, TRMT61B, TRMU, TRNT1, TRUB2, TSFM, TSHZ3, TSPO, TST, TSTD3, TTC19, TTC3, TUBB3, TUFM, TUSC2, TUSC3, TXN, TXN2, TXNDC12, TXNRD1, TXNRD2, TYMS, TYSND1, UACA, UBA1, UBIAD1, UCP1, UCP2, UCP3, UMPS, UQCC2, UQCR10, UQCR11, UQCRB, UQCRC1, UQCRC2, UQCRFS1, UQCRH, URI1, UROS, USMG5, USP30, USP48, UXS1, VAMP8, VARS, VARS2, VASN, VDAC1, VDAC2, VDAC3, VHL, VWA8, WARS2, WBSCR16, WDR81, WWOX, XAF1, XPNPEP3, XRCC3, XRCC6BP1, YARS2, YBEY, YKT6, YME1L1, YRDC, YWHAE, YWHAZ, ZBED8, ZDHHC8, ZFHX3, ZMIZ2, ZNF428 |
| **Fibrosis/extracellular matrix genes (n=951)** | |
|  | ABCA3, ABCB4, ABL1, ACACB, ACADL, ACADM, ACE, ACE2, ACER2, ACSL1, ACTA1, ACTC1, ADA, ADAM15, ADAM17, ADAM8, ADAM9, ADAMTS12, ADAMTS20, ADAMTS4, ADAMTS5, ADAMTSL2, ADAMTSL4, ADCYAP1, ADIPOQ, ADK, ADM, ADORA1, ADORA2A, ADORA2B, ADORA3, ADRA2A, ADRA2C, ADRB1, ADRB2, AGA, AGER, AGT, AGTR1, AGTR2, AHR, AKAP13, AKT1, ALB, ALK, ALOX5, AMBN, ANGPT1, ANGPT2, ANGPTL3, ANPEP, ANTXR1, ANXA1, APBB1, APBB2, APCS, APLP1, APLP2, APOA1, APOE, APP, APRT, AR, ARG1, ARG2, ARID4A, ARNT, ARRB2, ASXL1, ATF3, ATG7, ATP2A2, ATP7A, ATXN1L, AVP, AZIN1, B4GALT1, BARX2, BAX, BCAM, BCAP31, BCL2, BCL2L1, BCL2L11, BCL3, BCL6, BDKRB1, BDKRB2, BECN1, BHLHE40, BID, BLMH, BMP1, BMP2, BMP6, BMP7, BMPR1A, BMPR1B, BMPR2, BSCL2, BTC, BUB1B, C1GALT1, C3, C5, C6orf15, CACNA1C, CACNA1H, CALR, CAMLG, CAPNS1, CASP1, CASP8, CAV1, CAV2, CAV3, CBL, CC2D2A, CCDC80, CCL17, CCL2, CCL24, CCL5, CCND1, CCNE1, CCNG2, CCR1, CCR2, CCR5, CCR6, CD151, CD19, CD1D, CD28, CD36, CD4, CD40, CD40LG, CD44, CD46, CD55, CD5L, CD63, CD74, CD82, CD9, CD96, CDC37, CDH1, CDH11, CDK2, CDK4, CDK5, CDKN2A, CDSN, CEBPA, CEBPB, CEL, CELA2A, CFLAR, CFTR, CHRDL2, CIB1, CIDEC, CISH, CLSTN1, CLU, CMA1, CNOT3, CNR1, CNR2, CNTN2, COL13A1, COL17A1, COL18A1, COL19A1, COL1A1, COL1A2, COL27A1, COL2A1, COL3A1, COL4A3, COL4A6, COL5A3, COMP, COQ9, COX4I2, CPB2, Crb3, CRBN, CREB1, CREBBP, CREM, CRISPLD2, CRK, CRLF2, CSF1, CSF1R, CSF2, CSF3, CSGALNACT1, CSRP3, CST3, CTGF, CTNNA1, CTNNB1, CTSB, CTSH, CTSK, CTSS, CTTN, CX3CL1, CX3CR1, CXCL12, CXCL13, CXCL17, CXCL9, CXCR3, CYBB, CYP11B2, CYP1A2, CYP3A4, CYP3A5, CYP4B1, CYR61, CYSLTR2, DACT3, DAG1, DAGLA, DAGLB, DCN, DDIT3, DDR1, DDR2, DDX5, DES, DHFR, DICER1, DIO3, DLK1, DLL1, DMP1, DNAJB6, DNM1L, DNMT3A, DPP4, DPYSL2, DSP, DTNA, DYSF, EBI3, ECM2, ECSCR, Eda, EDN1, EDNRA, EDNRB, EGF, EGFL6, EGFLAM, EGFR, EGR1, EIF4EBP1, EIF4EBP2, ELANE, ELAVL1, ELF3, ELK1, ELN, EMILIN1, EMP2, ENPP1, ENPP2, ENTPD1, EPHA2, EPO, ERCC2, ERG, ETNK2, ETS1, EXOC8, EZH2, F11, F2R, F2RL1, F3, F5, F7, FAM111B, FAP, FAS, FASLG, FAT4, FBLN1, FBLN5, FCGR2B, FERMT2, FGA, FGB, FGF10, FGF6, FGFR1, FGFR2, FGFR3, FGFR4, FGG, FHL2, FKBP1A, FLOT1, FLOT2, FLT1, FLT3, FLT4, FMOD, FN1, FOSL2, FOXF1, FOXF2, FOXM1, FREM1, FSTL1, FSTL3, FYN, G0S2, GAS7, GCLC, GDF5, GDF6, GDNF, GFAP, GFOD2, GH1, GHRL, GLIS2, GLIS3, GNA13, GNAS, GNB3, GNE, GOLM1, GP5, GPX1, GRB2, GREM1, GSK3A, GSK3B, GSN, GSS, GSTP1, GZMB, HAS2, HBB, HBEGF, HCAR2, HCK, HDGF, HEXIM1, HEY2, HGF, HIF1A, HILPDA, HLA-DRA, HMGA2, HMGCS1, HMOX1, HNF4A, HOPX, HOXA7, HOXD3, HP, HPRT1, HPSE, HPSE2, HPX, HRAS, HS6ST1, HSD17B12, HSPB1, HSPB3, HSPB6, HSPB8, HSPG2, HTR1B, HTR2B, IBSP, ICAM1, ID1, IDH1, IDH2, IFNAR1, IFNAR2, IFNG, IFNGR1, IFNGR2, IFT88, IGF1, IGF1R, IGFBP3, IGKV1D-13, IHH, IKBKB, IKBKE, IKBKG, IL10, IL11, IL11RA, IL12A, IL12B, IL13, IL13RA2, IL16, IL17A, IL17RA, IL17RB, IL1B, IL1R1, IL1R2, IL1RN, IL2, IL21, IL22, IL23A, IL25, IL2RA, IL2RB, IL2RG, IL32, IL33, IL4, IL4R, IL5, IL5RA, IL6, IL6R, IL6ST, IL7, ILK, IMPDH1, IMPDH2, INHBA, INS, IRAK2, IRAK4, IRF2, IRF3, IRF5, ITGA1, ITGA10, ITGA11, ITGA2, ITGA2B, ITGA3, ITGA4, ITGA5, ITGA6, ITGA7, ITGA8, ITGAL, ITGAV, ITGB1, ITGB1BP1, ITGB2, ITGB3, ITGB4, ITGB5, ITGB6, ITGB7, JAK1, JAK2, JAM3, JUN, JUND, JUP, KAZALD1, KCNN4, KCP, KDR, KIF13A, KIF3B, KIF5B, KIF9, KIT, KL, KLF15, KLF5, KLK1, KLK4, KNG1, KRAS, KRT19, KRT8, L1CAM, LAMA2, LAMA4, LAMC1, LATS2, LCK, LCN2, LCP1, LDLR, LEP, LEPR, LGALS12, LGALS3, LGMN, LHX2, LIF, LIMS1, LIMS2, LIPE, LMNA, LMX1B, LOR, LOX, LPAR1, LPAR2, LPL, LRP1, LRP6, LRRC15, LTBP4, LYN, LYPD3, LYVE1, MADCAM1, MAGI2, MAN2A1, MAN2C1, MAP2K5, MAP3K5, MAPK11, MAPK14, MAPK8, MAS1, MB, MBNL1, MBNL2, MEF2C, MEF2D, MET, MIA, MIF, MKLN1, MLIP, MMP1, MMP11, MMP12, MMP13, MMP14, MMP2, MMP20, MMP3, MMP7, MMP8, MMP9, MPL, MPO, MPZL3, MSTN, MSX2, MTOR, MTSS1, Muc4, MUC5B, MYBPC1, MYBPC3, MYD88, MYF5, MYH2, MYH6, MYL2, MYLK3, MYO1E, MYOC, NACA, NAGLU, NAMPT, NAPEPLD, NCF1, NCOA5, NCOR2, NDNF, NDUFS6, NEXN, NF1, NF2, NFE2, NFE2L1, NFE2L2, NFKB1, NFKB2, NFKBIA, NGF, NID1, NID2, NLRP3, NME1, NOG, NOL3, NOS1, NOS2, NOS3, NOX1, NOX4, NOXO1, NPC1, NPC2, NPHP3, NPM1, NPNT, NPPA, NPPB, NPPC, NPR1, NPY1R, NR1H3, NR1H4, NR2E1, NR3C1, NR3C2, NRAS, NT5E, OLFML2A, OLFML2B, OR7E14P, ORMDL3, OSM, OTOA, P2RY1, P2RY12, PARN, PARVG, PAX7, PBK, PDCD1, PDCD6IP, PDE3B, PDE5A, PDGFB, PDGFC, PDGFRA, PDGFRB, PECAM1, PF4, PFKFB1, PFKFB3, PHB, PHEX, PIK3CA, PIK3CB, PIK3R1, PIKFYVE, PKD1, PKHD1, PLA2G10, PLA2G2A, PLA2G4A, PLA2G5, PLAC8, PLAT, PLAU, PLAUR, PLCL1, PLCL2, PLG, PLIN1, Pln, PLOD2, PLXND1, PNKD, PNPLA2, POLA1, POLB, POLD1, POMT1, POSTN, PPARA, PPARD, PPARG, PPAT, PPFIA1, PPFIA2, PPP1CB, PPP3CA, PPP3CB, PPP3CC, PPP3R1, PPP3R2, PRDX3, PRDX4, PRF1, PRKAA1, PRKAA2, PRKAR2B, PRKCB, PRKG1, PRKG2, PROC, PSEN1, PSEN2, PTEN, PTGER1, PTGIR, PTGIS, PTGS2, PTH, PTHLH, PTK2, PTPN11, PTX3, PXDN, RAB25, RAC1, RAF1, RAG1, RASA1, RASSF1, RASSF2, RBM17, RECK, RELA, RELB, REN, RERE, RET, RETN, RETNLB, RGS16, RGS5, RHOA, RNASEL, ROR2, RORA, RORC, RPGRIP1L, RRM2B, RTEL1, RUNX1, RUNX2, RXFP1, S100A10, S100A4, S1PR2, SCD, SCGB3A2, SCT, SDC2, SDC3, SDC4, SELE, SELL, SELP, SELPLG, SEMA3E, SEMA7A, SERAC1, SERPINB1, SERPINB5, SERPINB7, SERPINC1, SERPINE1, SERPINE2, SERTAD2, SETBP1, SF3B1, SFRP4, SFTPA1, SFTPA2, SFTPC, SFTPD, SGCA, SGCB, SGCD, SGCE, SGCG, SH2B3, SH3PXD2B, SHOX2, SIRPA, SIRT3, SIRT6, SIRT7, SKI, SKIL, SKP2, SLC18A3, SLC27A6, SLC2A4, SLC2A9, SLC4A1, SLC6A4, SLC7A11, SLC8A1, SLC9A1, SLIT2, SLN, SMAD2, SMAD3, SMAD4, SMAD7, SMARCA4, SMOC1, SMOC2, SMPD1, SMPD3, SMPX, SMURF2, SNAI1, SNCG, SOAT1, SOCS1, SOCS3, SOD1, SOD2, SOD3, SORBS1, SOST, SOX9, SP7, SPARC, SPHK1, SPINK5, SPINT1, SPOCK2, SPP1, SRC, SREBF1, SRF, SRSF2, SS18, STAB1, STAB2, STAT1, STAT3, STAT4, STAT6, STC1, STK11, STK25, STK3, STK4, STMN1, SYT7, SYVN1, TBX5, TBXA2R, TEAD1, TEK, TERC, TERT, TET2, TGFA, TGFB1, TGFB1I1, TGFB2, TGFBI, TGFBR1, TGFBR2, TGIF1, THBD, THBS1, THBS2, THBS4, THY1, TIAM1, TIMP1, TIMP2, TIMP3, TLN1, TLR2, TLR4, TM4SF1, TMEM67, TMEM8B, TNF, TNFAIP3, TNFAIP6, TNFRSF11B, TNFRSF13B, TNFRSF1A, TNFRSF1B, TNFSF11, TNFSF12, TNFSF14, TNN, TNNC1, TNNI3, TNNT2, TNR, TNS1, TNXB, TOP1, TP53, TP63, TPM1, TPM2, TPSG1, TRAF3IP2, TRAF6, TRDN, TREX1, TSC1, TSC22D1, TSLP, TSTA3, TUBA1A, TUBA1C, TUBA4A, TUBA8, TUBB1, TUBB2A, TUBB3, TUBB4A, TUBB4B, TUBD1, TUBE1, TUBG1, TUBG2, TWIST1, TWSG1, TYK2, TYMS, UBD, UGCG, UMOD, Uox, VAV2, VAV3, VCAM1, VCAN, VCL, VDR, VEGFA, VEGFB, VHL, VIM, VIT, VTN, VWA1, VWF, WFDC2, WNT3A, WRN, WT1, XDH, XIRP1, XYLT2, YAP1, YES1, YY1, ZYX |
| **IFNγ-modulated genes/Th1 response genes (n=1179)** | |
|  | ABCB8, ABHD14A, ACAT2, ACIN1, ACOT9, ACOX1, Acp5, ACSL1, ACTB, ACTG2, ACTN1, ACVRL1, ADA, ADAM8, ADAR, ADCY9, ADD1, ADORA2A, AGAP1, AGPAT5, AGRN, AGTRAP, AHNAK, AHR, AIM1, AK2, AKR1B10, AKT1, AKT3, ALCAM, ALDH2, ALDOA, ALDOC, ALG3, AMFR, ANK1, ANKH, ANKRD28, ANKRD40, ANKRD46, ANTXR2, ANXA1, ANXA2, AOAH, AP3M1, APBB1, APLP2, APOA1, APOD, APP, AQP4, ARAP3, ARF6, ARFGEF1, ARHGEF3, ARL4D, ARL5A, ARL5B, ARL6IP4, ASAP1, ASPH, ASS1, ASXL1, ATF3, ATG4B, ATL2, ATM, ATP13A3, ATP1A1, ATP1B1, ATP1B3, ATP2A2, ATP2C1, ATP5A1, ATP5B, ATP5C1, ATP5F1, ATP5G1, ATP5G2, ATP5G3, ATP5J, ATP6V1A, ATP6V1E1, ATP9A, ATPIF1, ATXN10, ATXN7L3, AUH, AXL, B2M, B4GALT6, BATF3, BAZ2A, BAZ2B, BCL2L13, BCL3, BCL6, BIRC2, BMP1, BOP1, BRD2, BROX, BST1, BTG1, BTLA, BUB3, C15orf39, C16orf72, C19orf60, C1orf43, C1QA, C1R, C2, C20orf24, C3, C3AR1, C3orf38, C5AR1, C6orf106, C8orf33, CA5A, CAB39, CAMK1, CAMLG, CAPN1, CAPN2, CAPNS1, CARD9, CASD1, CASP1, CASP3, CASP4, CASP6, CASP9, CAT, CBL, CCL2, CCL22, CCL3, CCL3L3, CCL4, CCL5, CCL7, CCND3, CCNG2, CCNH, CCR1, CCR2, CCR5, CCR6, CCRL2, CCS, CCT6A, CCZ1, CD14, CD180, CD1D, CD226, CD244, CD247, CD274, CD276, CD28, CD38, CD40, CD40LG, CD44, CD47, CD55, CD74, CD80, CD81, CD86, CD9, CDC16, CDC25A, CDC5L, CDK18, CDK2AP1, CDKN1A, CDKN2A, CDR2, CDR2L, CEACAM1, CEBPB, CEBPZ, CENPW, CFB, CFLAR, CHCHD7, CHMP4B, CHRNA7, CIITA, CISD1, CKB, CKMT1A, CLCN3, CLCN4, CLCN5, CLEC4D, CLEC7A, CLIC1, CLIP1, CLK1, CLNS1A, CLTA, CMAS, CMPK2, CNN2, CNOT6L, COIL, COL12A1, COL4A1, COL4A2, COL5A1, COL7A1, COL9A3, COPS3, CORO1A, CORT, COX16, Cox5b, COX6A1, COX8A, CPXM2, CR1L, CR2, CRAT, CREB3, CRNKL1, CRP, CRTAM, CRYZ, CSF2, CSF3R, CSTF2, CSTF3, CTLA4, CTNNA1, CTNND1, CTSC, CTSD, CTSH, CTSS, CTSZ, CUL2, CX3CL1, CXCL10, CXCL11, CXCL12, CXCL3, CXCL9, CXCR2, CXCR3, CXCR4, CYBB, CYC1, CYCS, DAB2, DARS, DAXX, DBH, DBI, DBNL, DCAF12, DCK, DCTPP1, DDB1, DDHD2, DDX1, DDX54, DEGS1, DENND5A, DGCR2, DHRS7, DHX16, DHX32, DHX40, DICER1, DLAT, DLGAP4, DLL1, DLL4, DNAJB1, Dnajb3, DNAJC2, DNAJC5, DPP7, DPT, DPYSL3, DRD2, DRD3, DRG1, DUSP1, DYNC1H1, E2F3, EBI3, EEF1B2, EEF1D, EEF2, EEF2K, EFNB1, EI24, EIF2B1, EIF4B, EIF4G1, ELF3, ELK1, ELOVL1, ELOVL3, EMP1, ENG, ENO2, ENPP2, ENPP5, ENTPD1, EPRS, ETFB, ETS1, EVI5, EVL, EXOC8, EZH2, FABP4, FABP5, FAM105A, FAM107B, FAM114A2, FAM136A, FAM13B, FAM20C, FAM50A, FAM65B, FANCC, FARS2, FARSB, FAS, FASLG, FASN, FASTK, FBXO11, FBXO6, FCER1G, FCGR2A, FCHO1, FDPS, FGF11, FGFR1, FGR, FHL1, FKBP1A, FKBP4, FLII, FLNA, FLNB, FLT3LG, FNBP1, FNTA, FOS, FOXC2, FOXF1, FRMD6, FRRS1, FTH1, FURIN, FUT4, FUT7, FXYD5, G3BP2, G6PD, GABARAPL2, GABRQ, GADD45B, GADD45G, GALNT4, GAMT, GANAB, GAS6, GATA3, GBA, GBP2, GBP3, GBP4, GBP7, GCLC, GCNT1, GDF5, GDI1, GET4, GFPT1, GFRA3, GGA2, GGCT, GIT1, GLI2, GLRX3, GLUD1, GNA13, GNAI2, GNAS, GNB2, GNB4, GNG5, GNS, GOLM1, GOT1, GOT2, GPC1, GPD2, GPR12, GPR137B, GPR65, GPS1, GPX3, GPX4, GRHL1, GRWD1, GSN, GSTM3, GSTM5, GSTO1, GSTZ1, GTF2B, GTF2E1, GTF2H5, GTF3C1, GTF3C4, GTPBP2, GUCA1A, GUSB, H2AFV, Havcr1, HAVCR2, HDAC2, HELB, HERPUD1, HEXDC, HFE, HIF1A, HIST1H1C, HIVEP1, HK2, HLA-A, HLA-DMA, HLA-DMB, HLA-DOA, HLA-DQA1, HLA-DQB1, HLA-DRB1, HLA-DRB5, HLA-E, HLA-G, HMBOX1, HMGA1, HMGB1, Hmgb3, HMGCL, HMGCS1, HMOX1, HNRNPA0, HNRNPA2B1, HNRNPC, HNRNPL, HOXB4, HPGD, HPS1, HRAS, HRH1, HRH2, HSD17B4, HSF1, HSPA4, HSPA4L, HSPA8, HSPB1, HUWE1, IARS2, ICAM1, ICAM2, ICOS, ID1, IDE, IDH1, IDH2, IDI1, IFI16, Ifi27, IFI30, IFI35, IFIH1, IFIT1B, IFIT2, IFIT3, IFITM2, IFITM3, IFNA10, IFNA14, IFNA16, IFNA2, IFNA21, IFNA4, IFNA6, IFNA7, IFNA8, IFNAR1, IFNB1, IFNE, IFNG, IFNGR2, IFNK, IFNW1, IFT20, IGFBP1, IGFBP4, IGHMBP2, IL10, IL10RA, IL10RB, IL12A, IL12B, IL12RB1, IL12RB2, IL15, IL15RA, IL16, IL17A, IL17RA, IL18, IL18BP, IL18R1, IL1B, IL1RN, IL2, IL20RB, IL21, IL21R, IL23A, IL23R, IL27, IL27RA, IL2RG, IL33, IL4, IL4R, IL6, IL9, IL9R, IMP4, IMPA1, INHBB, INPP1, INPP5J, IPO4, IRAK1, IRAK4, IRF1, IRF2, IRF3, IRF4, IRF5, IRF7, IRF8, ISG15, ISYNA1, ITGA3, ITGA4, ITGAL, ITGAM, ITGB1, ITGB2, ITGB5, ITGB7, ITK, JAK3, JARID2, JUN, KARS, KCNJ3, KCNMA1, KDELR1, KDELR2, KIAA1217, KLF10, KLF6, KLRC1, KLRD1, Lage3, LAMC1, LAMP1, LAMP2, LARP1, LARS, LCN2, LCP2, LDLR, LEF1, LEP, LGALS1, LGALS3BP, LGALS9, LILRB3, LIMA1, LMAN2, LMO4, LPGAT1, LPIN2, LRP6, LRRC40, LRRC8C, LSS, LTA, LTBP3, LXN, LY6E, LY86, MAF, MAFB, MAFG, MAGED2, MAK, MALT1, MAN1A1, MAN2A1, MAP2K2, MAP2K3, MAP3K4, MAP3K8, MAP4K4, MAPK1, MAPK3, MAPK8, MAPK9, MAPRE1, MARK3, MARK4, MAST3, MAX, MBD6, MBP, MCRS1, MDM2, MECR, MED22, MERTK, MFAP1, MFGE8, MGP, MGST3, MIF, MITF, MLEC, MLST8, MMP12, MOB2, MOG, MOGS, MORC4, MORF4L2, MOV10, MPEG1, MPO, MPP1, MPRIP, MPZ, MRC1, MRPL18, MRPL3, MRPL30, MRPL39, MRPL49, MRPS31, MTCH1, MTCH2, MTM1, MTOR, MTPN, MYCBP2, MYD88, MYEF2, MYL6, MYO1C, MYO1E, N4BP1, NAA10, NAB2, NACA, NAE1, NAMPT, NAP1L4, NAV1, NCBP2, NCF1, NCL, NCOA4, NCOR1, NCOR2, NDRG1, NDUFB5, NDUFS3, NDUFS4, NDUFV2, NEDD8, NF2, NFATC2, NFATC3, NFKBIZ, NKIRAS1, NKX1-2, NLRP10, NME3, NMI, NMT1, NOA1, NOC4L, NOD2, NOP2, NOS2, NOTCH2, NOTCH3, NPY1R, NR1H2, NR2F6, NRAS, NT5DC2, NT5E, NUB1, NUDT19, NUFIP1, NUMB, NUS1, NXF1, OAT, OAZ1, OAZ2, OGFR, ORC2, OSBPL11, OSBPL9, Otud5, OTUD7B, OXR1, PAFAH1B3, PARK7, PARP1, PARP8, PC, PCBD2, PCID2, PCSK1, PCSK4, PDCD2, PDCL3, PDGFA, PDIA3, PDLIM2, PDSS2, PDZK1IP1, PEA15, PEBP1, PELI1, PEPD, PES1, PF4, PFKP, PGLYRP1, PGM1, PGS1, PHC2, PHLDA1, PHLDB2, PIAS3, PIK3CA, PIK3CD, PIK3CG, PIK3R1, PIM1, PIP4K2C, PISD, PKD2, PLA2G2C, PLA2G4A, PLA2G7, PLAC8, PLAUR, PLBD1, PLCB3, PLD4, PLP1, PLXNA1, PML, PMP22, PNP, POLR2G, POLR2J, POSTN, POU2AF1, POU2F3, PPAN, PPARG, PPFIBP2, PPIA, PPIE, PPP1R15B, PPP2CB, PPP3CA, PPT1, PRC1, PRDX5, PRDX6, PRKAA1, PRKAB1, PRKACA, PRKAG1, PRKAG2, PRKCQ, PRNP, PROCR, PRPF38A, PRPS2, PSEN1, PSEN2, PSMA2, PSMA3, PSMA5, PSMA7, PSMB10, PSMB2, PSMB8, PSMB9, PSMC6, PSMD12, PSMD7, PSME1, PSME2, PSMG4, PTGER1, PTGIR, PTGR1, PTGS2, PTH1R, PTK2, PTPN2, PTPN22, PTPN6, PTPRG, PTPRS, PTTG1, PURA, PYCARD, QKI, RAB14, RAB1A, RAB2A, RAB31, RAB33B, RAB3D, RABEP1, RABGAP1L, RAD51B, RAE1, RAG2, RALA, RALGDS, RAN, RANBP10, RANGRF, RAP1GDS1, RARS, RASA1, RAX, RBCK1, RBM22, RBM38, RBM39, RBM4, RBP3, RBP4, RCAN1, RCC2, RCOR1, REEP5, REG3G, REL, RELB, RELL1, RENBP, RETSAT, RGL1, RGS1, RGS14, RGS16, RGS19, RHOB, RHOQ, RIPK2, RIT2, RLIM, RNASE4, RNASEH2A, RNASEK, RNASET2, RNF138, RNF14, RNF145, RNF19A, ROCK1, ROCK2, RORC, RPL10, RPL10A, RPL12, RPL13, RPL17, RPL18, RPL21, RPL23, RPL27A, RPL28, Rpl36a, RPL5, RPL7A, RPL8, RPLP2, RPS13, RPS15, RPS15A, RPS16, RPS17, RPS18, RPS2, RPS26, RPS3, RPS4Y1, RPS5, RPS6KA3, RPS6KC1, RPS8, RPSA, Rrbp1, S100A4, S100PBP, S1PR1, S1PR4, SACM1L, SAMHD1, SAMSN1, SARDH, SBF2, SCOC, SDC3, SDHB, SEC14L1, SEC16A, SEC22B, SEC61A1, SELE, SELL, SELP, SELPLG, SEMA4A, SEMA4B, SEMA6D, SERF2, SERPINA3, SERPINB2, SERPINB6, SERPINB9, SERPING1, SFPQ, SFXN2, SH2D1A, SH3GL1, SHC1, SIDT2, SIT1, SKI, SLC11A1, SLC11A2, SLC16A2, SLC1A5, SLC22A17, SLC22A18, SLC23A2, Slc25a1, SLC25A4, SLC25A5, SLC27A1, SLC29A1, SLC2A1, SLC30A4, SLC31A1, SLC35B1, SLC39A6, SLC3A2, SLC44A1, SLC4A2, SLC5A6, SLC6A6, SLTM, SMARCA4, SMARCE1, SMC3, SMYD2, SNRK, SNRPB, SNTA1, SNX1, SNX3, SOAT2, SOCS1, SOCS3, SOD1, SOD2, SPAG7, SPATS2, SPECC1, SPP1, SPR, SPRYD7, SPTAN1, SPTLC1, SQLE, SQRDL, SREBF1, Srgn, SRM, SRPX, SRSF6, SS18, SSR4, SSRP1, ST3GAL5, STAB1, STAT1, STAT3, STAT4, STAT5B, STAT6, STX2, STXBP1, STYK1, SUN2, SURF1, SURF2, TAP1, TAP2, TAPBP, TARS, TAX1BP3, TBCEL, TBX21, TBXAS1, TCF12, Tchh, TDG, TECR, TERF2IP, TERT, TFB2M, TGFB1, TGM2, TGS1, TH, THOP1, THPO, TIMM10, TIMM23, TIMM44, TIMP2, TIRAP, TKT, TLR2, TLR3, TLR4, TLR7, TLR9, TM7SF2, TM9SF2, TMEM109, Tmem115, TMEM26, TMEM50B, TMEM55A, TMX1, TNF, TNFAIP2, TNFRSF1A, TNFRSF21, TNFSF12, TNFSF14, TNPO1, TOB1, TOLLIP, TOP1, TOR1AIP2, Tpm1, TPR, TPST1, TRAF1, TRAF3, TRAF5, TRAF6, TRAFD1, TRAPPC1, TRAPPC10, TRIM21, TRIM27, TRIM47, TRPM4, TSLP, TSNAX, TSPAN3, TSPAN33, TSPO, TSPO2, TTC7B, TUBA1A, TUBA1B, TUBB, TUBB2A, TUBB3, TUBGCP4, TULP4, TXN, TXNL4A, TYK2, UBA7, UBAC1, UBE2H, UBE2L6, UBN1, UMPS, UPF3B, UPP1, UQCR10, UQCRQ, USP15, USP18, USP39, USP9X, UTP18, VAMP3, VASP, VCAM1, VCAN, VDAC2, VDAC3, VEGFA, VIP, VOPP1, VPREB1, VPS26A, VPS35, VPS45, VPS72, VTCN1, WARS, WDFY3, WDR1, WDR6, WDR92, WNK1, WNT10A, XPO7, XRCC6, YBX1, ZFAND6, ZFHX3, ZFP36, ZFP64, ZMYM4, ZNF281, ZNF358, ZNF426, ZNF623, ZNFX1, ZSCAN21, ZW10, ZYX |
| **Hypertrophy genes (n=605)** | |
|  | A4GALT, ABCA1, ABCC4, ABCC8, ABCD2, ABL1, ACE, ACE2, ACTC1, ACTG2, ADA, ADAM17, ADCY6, ADIPOQ, ADK, ADM, ADORA1, ADORA2A, ADORA3, ADRA1A, ADRA1B, ADRA1D, ADRA2A, ADRA2C, ADRB1, ADRB2, ADRB3, AGER, AGT, AGTR1, AGTR2, AHR, AKAP1, AKAP13, AKAP5, AKT1, AKT1S1, ALOX15, ANGPT1, ANGPT2, ANKRD1, APAF1, APLN, APOE, APP, AR, ARAF, ATF2, ATF3, ATM, ATP2A2, AVP, BAG6, BAX, BCL2, BCR, BDNF, BIRC5, BMP10, BMP2, BMP4, BMP7, BMPR1A, BMPR2, BMX, BRAF, C3, CA2, CABIN1, CACNA1C, CACNA1D, CACNA1H, CACNA1S, CACNA2D1, CACNB2, CACNB3, CAMK2D, CAMK4, CASQ2, CAST, CAT, CAV1, CAV3, CCK, CCKAR, CCKBR, CCNT1, CCR2, CD2AP, CD36, CDK4, CDK5RAP1, CDK9, CDKN1A, CDKN1B, CEBPB, CETP, CFTR, CGA, CHGA, CIB1, CKM, CKMT2, CMKLR1, CNOT3, CNTF, COL1A1, COL9A2, CORIN, COX4I2, CPT1A, CPT2, CREB1, CREM, CRYAB, CSF2, CSF3, CSRP3, CTF1, CTGF, CTNNB1, CTSB, CTSC, CTSD, CXCL12, CYBA, CYBB, CYP19A1, CYP1A2, CYP1B1, CYP2J2, DACT1, DAG1, DBI, DEPTOR, DES, DGKZ, DICER1, DMD, DNAJC3, DPF3, DPP4, DRD2, DTNBP1, DUSP1, DUSP5, DYRK1A, E2F2, ECE1, ECM1, EDN1, EDNRA, EEF1D, EGF, EGFR, EGLN1, EHD3, EHD4, EI24, EIF4EBP1, ELN, EMP1, ENDOG, ENPP7, EP300, EPAS1, EPO, EPOR, ERG, ESR1, ESR2, EXT2, F2R, F2RL1, FABP3, FASLG, FASN, FBXO32, FGF2, FGF23, FGF3, FHL1, FHL2, FKBP1A, FKBP1B, FLNB, FLT1, FN1, FNIP1, FOXM1, FOXO1, FOXO3, FSHR, FST, FSTL3, FUBP1, FXYD1, GAA, GAL, GATA4, GATA6, GDF15, GDNF, GFAP, GH1, GIT, GJA1, GLA, GLRX3, GNA11, GNAI2, GNAQ, GPX1, GPX3, GRB2, GRK5, GRN, GRP, GSK3B, GSN, GUCY1A3, GUCY2C, H2AFZ, HAND1, HAND2, HBEGF, HCK, HDAC2, HDAC3, HDAC4, HDAC5, HDAC9, HES1, HES5, HEXIM1, HEY2, HGF, HIF1A, HMGA1, HMGB1, HMGCR, HMOX1, HOPX, HPRT1, HRAS, HRH2, HSD11B1, HSD11B2, HSD17B2, HSPB2, HSPB8, HTR2A, HTR2B, HTR2C, HTT, ICAM1, IER3, IGF1, IGF2R, IHH, IKBKB, IKBKE, IL11, IL13, IL18, IL1B, IL1RL1, IL25, IL33, IL4, IL5, IL6, IL6R, IL6ST, IL7, IL9, INHA, INHBA, INPPL1, IRX4, ITGB3, JAK2, JARID2, JPH2, JUN, KCND2, KCNE2, KCNE3, KCNJ11, KCNQ1, KL, KLF15, KLF4, KLF5, LAMA4, LCAT, LEMD3, LEP, LEPR, LGALS9, LHCGR, LIF, LIN28B, LMCD1, LRP5, LTK, LYN, MAGI2, MAP2K1, MAP2K3, MAP2K4, MAP2K5, MAP2K6, MAP2K7, MAP3K5, MAP3K7, MAPK1, MAPK14, MAPK3, MAPK7, MAPK8, MB, MBNL1, MBNL2, MBTPS1, MC3R, MC4R, MDM2, MED1, MED13, MEF2A, MEF2C, MEF2D, MEX3C, MIF, MKL1, MLIP, MMP1, MMP19, MMP3, MMP7, MMP9, MNT, MORF4L1, MPZ, MRVI1, MSTN, MTOR, MTPN, MUS81, MYBPC3, MYC, MYH10, MYH14, MYH6, MYH7, MYL2, MYL3, MYL9, MYLK2, MYLK3, MYOCD, MYOD1, MYOM1, MYOZ2, MYZAP, NAB1, NCF1, NF1, NFATC1, NFATC2, NFATC3, NFATC4, NFKB1, NFKBIA, NGF, NKX2-5, NKX3-2, NODAL, NOG, NOS1, NOS2, NOS3, NOX1, NOX4, NPHS2, NPPA, NPPC, NPR1, NPR2, NR1H3, NR1I3, NR3C1, NR3C2, NR4A3, NRAS, NT5E, NTF3, ODC1, OSM, P2RX4, PAFAH1B1, PARK7, PARP1, PBX1, PBX2, PBX3, PDC, PDE5A, PDE9A, PDGFA, PDGFC, PDPK1, PFKFB1, PFKM, PFN1, PGAP2, PGAP3, PGF, PGR, PHC1, PIGO, PIGV, PIGW, PIGY, PIK3CA, PIK3CG, PIK3R1, PIM1, PITX2, PLA2G4A, PLAT, PLAU, PLCB1, PLCE1, PLD1, PLIN1, PLIN5, PNKD, PNPLA2, PNPLA8, POSTN, POU3F1, PPARA, PPARD, PPARG, PPARGC1A, PPARGC1B, PPIA, PPP3CA, PPP3CB, PPP3R1, PRDX3, PRKAA1, PRKAA2, PRKCA, PRKCB, PRKCD, PRKCE, PRKCI, PRKG1, PRKG2, PRMT5, PTEN, PTGES, PTGIR, PTGIS, PTGS2, PTH1R, PTHLH, PTK2, PTPN11, PTTG1, RAB1A, RAB2A, RAB4A, RAC1, RAF1, RAP1A, RAPGEF3, RARA, RARB, RASSF1, RBP4, RCAN1, RCAN2, RCBTB1, REN, RGS2, RGS4, RGS5, RHEB, RHOA, RNLS, ROCK2, RPS6KA2, RRAD, RSPO2, RSPO3, RUNX2, RYR2, S100A10, S100A6, SCARB1, SCNN1B, SEMA3A, SERPINE1, SETDB1, SGCG, SGK1, SHC1, SIRT1, SIRT3, SIRT6, SIRT7, SIX1, SKP2, SLC18A3, SLC25A4, SLC2A4, SLC4A1, SLC6A4, SLC8A1, SLC9A1, SLC9A3R1, SLIT2, SMAD3, SMAD4, SMAD6, SMAD7, SMARCA4, SMTN, SNCG, SNTA1, SOD1, SOD2, SOST, SOX9, SP3, SPEG, SRC, SREBF1, STAB1, STAB2, STAT3, STC1, STK3, SULT1E1, TAB1, TBX5, TCAP, TCF15, TCF4, TERC, TERT, TG, TGFB1, TGFBR2, TIAM1, TIMP1, TIMP3, TLN1, TLR2, TLR3, TLR4, TNF, TNFAIP3, TNFRSF11B, TNFRSF13B, TNFSF12, TNNC1, TNNI3K, TNNT2, TP53, TPM1, TPPP2, TRDN, TRIM54, TRIM55, TRIM63, TRPC1, TRPC3, TRPC4, TRPC6, TRPM7, TRPV1, TSC2, TSHR, TTN, TWF1, TXN, UCN, UCN2, VAV2, VAV3, VDR, VEGFA, VEGFB, VIP, WNT5A, WNT5B, WNT7A, XIRP1, YAP1, ZNF260 |
| **Muscle contraction and contractility genes (n=178)** | |
|  | ABCC4, ACE2, ADCY6, ADH5, ADIPOQ, ADM, ADRA1B, ADRB1, ADRB2, ADRB3, AGRN, AKAP10, AKAP5, ANGPT2, ANK2, ANK3, ANXA6, APLN, APLNR, APOB, APOE, ARRB1, ARSB, ASPH, ATE1, ATP1A1, ATP1A2, ATP2A2, AVP, BCAR1, BDNF, BIRC2, BIRC3, BTC, CACNA2D1, CASP3, CASQ2, CAV1, CAV3, CDK5RAP1, CHGA, CHRM1, CKM, CLU, CNOT3, CORIN, COX7A1, CSRP3, CTNNA3, CYP19A1, DES, DIO2, DUSP8, EDN1, EDNRA, EDNRB, EFNA1, EIF4EBP1, ENO1, EP300, FBXO32, FGF2, FKBP1A, FKBP1B, FXYD1, GAB1, GAB2, GATA5, GCG, GHRL, GNAQ, GNB1, GRK2, HAND2, HBEGF, HEY2, HMOX1, HSPB6, HSPB8, HTR1B, ICAM1, IDUA, IER3, IL18, IL6, INHBA, IRX4, ITGB3, IVNS1ABP, JUP, KLK3, LAMA4, LAMP2, LDLR, LEP, LIMS2, LMNA, LMOD2, MAP3K1, MAPK1, MAPK8, MB, MEF2C, MMP9, MTMR14, MYBPC3, MYH6, MYH7, MYLK, MYLK3, NCF1, NEDD4L, NEXN, NOL3, NOS1, NOS2, NOS3, NRG1, PDC, PDCD1, PDLIM5, PFKFB1, PIK3CG, PLA2G10, PLA2G5, PLA2G6, PLAT, PLAU, PLCE1, PLIN5, PLN, PNPLA2, PPARGC1A, PRKCA, PRKCB, PRKCG, PRKD1, PRKG1, PTH1R, RAB1A, RNLS, RRAD, RXRA, S100A1, SHC1, SLC4A1, SLC6A4, SLC6A6, SLC8A1, SLN, SMAD7, SOD1, SPP1, SRL, SRSF1, SUMO1, TERC, TIMP3, TLR2, TLR4, TLR5, TNF, TNNI1, TNNT2, TRIM21, TRIM54, TRIM55, TRIM63, TRPM2, TTN, TXNIP, VAV3, VCL, VDR, VEGFA, XDH, XIAP, XIRP1 |
| **Oxidative stress/Antioxidant response genes (n=580)** | |
|  | ABCB10, ABCB6, ABCC1, ABCC2, ABCG2, ABHD4, ABL1, ACE, ACOT11, ACTL8, ADAM23, ADAMTS12, ADAMTS13, ADAMTS16, ADD2, ADIPOQ, ADM, AGT, AHR, AIFM1, AIFM2, AKR1B1, AKR1B10, AKR1C1, AKR1C3, ALDH1A1, ALDH3A1, ALDH3A2, ALOX12, ALS2, AMBP, ANGPT2, ANGPTL7, APOE, APP, ARHGAP28, ASF1A, ASNS, ASPH, ATF4, ATOX1, ATP2A2, ATP5L, ATPAF1, ATRN, B4GALNT1, BAK1, BAX, BBOX1, BCAT1, BCKDK, BCL2, BHLHB4, BRCA1, BRIP1, C10orf90, C14orf149, C15orf42, C16orf28, C19orf12, C1orf186, C1orf84, C1orf90, C1S, C20orf177, C20orf42, C22orf29, C6orf107, C6orf117, CA3, CA5A, CA5B, CALCB, CALN1, CAMK1, CAMK1G, CAMK2G, CAMKK1, CASP10, CAT, CBR1, CBR3, CBS, CBS/CBSL, CBWD2, CCDC77, CCL17, CCND3, CDC25A, CDCA4, CDH12, CDK5RAP1, CDK5RAP2, CDKN2B, CES1, CHAC1, CHRNA4, CLCC1, CLDND2, CLEC12A, CLN5, CLN8, COQ7, COQ9, CPLX1, CPLX2, CPN1, CPXCR1, CST3, CSTA, CXCR7, CYB5R3, CYB5R4, CYBB, CYGB, CYP2E1, CYP4F11, CYP4F12, CYP4F2, CYP4F8, DBF4B, DDC, DDIT3, DDX43, DEFB119, DEGS1, DGKG, DGKK, DHFR, DHODH, DIABLO, DKC1, DKFZp564N2472, DKFZp762E1312, DMD, DNA2L, DNAH8, DNAJA5, DNAJB9, DOCK10, DRD5, DTL, DUSP1, DUSP13, ECM2, EFEMP2, EIF2AK4, EIF2S2, EMR3, EPAS1, EPHX1, ERCC1, ERCC2, ERCC3, ERCC6, ERCC8, ETFDH, F2RL2, FABP1, FAM55C, FBXL5, FBXO30, FEN1, FGF7, FGF8, FIGF, FLJ20489, FLJ22184, FLJ23861, FLJ25416, FLJ25801, FLJ35767, FOXRED2, FTH1, FTHL17, FTL, FUT1, FXYD7, FZD7, G6PD, GAB1, GALNT13, GAS2, GCK, GCKR, GCLC, GCLM, GCNT3, GDAP1, GGA2, GGT5, GINS3, GLA, GMFB, GOT1L1, GPC1, GPD2, GPNMB, GPR135, GPR68, GPT2, GPX1, GPX2, GPX7, GRK2, GSR, GSS, GSTA2, GSTA5, GSTZ1, HFE, HGD, HINT2, HIST1H2AB, HMOX1, HMOX2, HNF1A, HNMT, HNRPD, HNRPDL, HPD, HPDL, HPN, HPS1, HRG, HRK, HSD17B10, HSPB1, HSPB2, HTATIP2, HTT, HYDIN, ICMT, IDH1, IFITM5, IGF2, IL33, IMPACT, INADL, INSR, IPCEF1, ISCU, JAK2, JUN, KCNA1, KCNE4, KEAP1, KIAA0232, KIAA0319, KIAA0368, KIAA1333, KLC1, KLHDC5, KPNA5, KRTAP4-10, LANCL1, LANCL3, LBR, LDLR, LEP, LGALS3, LIAS, LOC283871, LOC338328, LOC389118, LOXL3, LRAT, LRP12, LRP8, LRRK2, LTB4DH, MAFG, MAG1, MAGEA1, MAOB, MAP1B, MAP2, MAP2K1, MAPK14, MAPT, MARS, MAT2A, MC3R, MCM10, ME1, MEGF9, MEIS1, MET, MFN2, MGC27121, MGST1, MICB, MMP2, MMP9, MPN2, MPO, MPP3, MRPL52, MSC, MSRA, MSTN, MT-CO1, MTF1, MTHFD2, MT-ND3, MUC1, MUTYH, MYC, MYH11, MYL2, NAMPT, NAP1L2, NAPRT, NCF2, NDUFA12, NDUFA4L2, NDUFA6, NDUFB4, NDUFC2, NDUFS2, NDUFS4, NDUFS8, NEIL1, NEIL2, NEIL3, NFE2L1, NFE2L2, NFKB1, NGFR, NINJ2, NKPD1, NLN, NME8, NOS2, NOS3, NOTCH3, NOX4, NQO1, NQO2, NR0B1, NR3C2, NR4A2, NR4A3, NRCAM, NRF1, NRG4, NUDT1, NUMBL, NUPR1, OGDH, OGG1, OLFM2, OPN1SW, OR1F1, OR1S2, OR6K2, ORAI1, ORC1L, ORC6L, OSCAR, OSGIN1, OSGIN2, OXR1, OXSR1, P2RY6, PA2G4, PABPC3, PANX2, PARK7, PARP10, PCK2, PCLAF, PDE6B, PDHA1, PDK1, PDLIM1, PDSS2, PEMT, PEPP-2, PEX11B, PEX13, PFN2, PGD, PHGDH, PIK3R1, PINK1, PIR, PLAA, PLIN5, PNKP, POL3S, POLQ, PON1, PON2, PON3, POPDC3, POU3F3, PPARG, PPARGC1A, PPARGC1B, PPAT, PPFIA2, PPIA, PPID, PPIF, PPP1R15B, PPP2R2C, PRDX1, PRDX2, PRDX3, PRDX4, PRDX5, PRDX6, PRKCD, PRKN, PRNP, PRR5, PSAT1, PSEN1, PSIP1, PSMB5, PTEN, PTPRD, QDPR, RAB42, RAC1, RBP1, RBPMS, RCAN1, RCAN2, RDM1, REEP1, REN, RFX4, RGN, RGS14, RHBDL3, RIPK2, RKHD1, RNF212, RP11-93B10.1, RPP30, RPS3, RRM2, RRM2B, RXRA, S100A12, SCARA3, SCD, SELENOF, SELENOK, SELENOP, SEMA3G, SERPINA12, SERPIND1, SETMAR, SFN, SFTPA1, SGK2, SGMS1, SHC1, SIL1, SIRT1, SIRT3, SKP2, SLC11A1, SLC14A2, SLC15A1, SLC19A1, SLC1A4, SLC31A1, SLC38A6, SLC47A1, SLC5A11, SLC6A6, SLC7A11, SLCO2B1, SMPX, SMURF1, SNCA, SNPH, SNRP70, SOD1, SOD2, SORD, SOX3, SPG20, SPP1, SPPL2B, SQSTM1, SRGN, SRI, SRXN1, ST3GAL4, STC2, STEAP4, STIM1, STK25, STK4, SUHW3, SULT1A2, SULT1E1, SUV39H2, TALDO1, TARDBP, TAT, tcag7.1017, TDO2, TERT, TFAM, TFRC, TGFB1, TKT, TLCD1, TLR4, TLR6, TM4SF20, TMEM174, TMEM177, TMEM18, TMEM20, TMEM50A, TMPO, TNF, TNFSF15, TNIP3, TOP1MT, TOR1A, TP53, TP53INP1, TP73, TRDMT1, TREX1, TRIM16, TRIM29, TRPM2, TSKU, TSPAN7, TSPAN9, TWSG1, TXN, TXNDC2, TXNIP, TXNRD1, UBE4B, UCHL1, UCN, UCP3, UGDH, UGT1A6, UGT1A8, USP10, USP31, USP6, VASN, VAV1, VCP, VHL, VLDLR, VNN1, VSIG1, WDR33, WDR76, WNT11, WRN, XDH, XPA, XPC, XPOT, XYLB, YBX1, ZDHHC21, ZFP36, ZNF326, ZNF429, ZNF467, ZNF649, ZNF706, ZNF771, ZSCAN10 |
| **Familial cardiomyopathy genes (n=113)** | |
|  | AARS2, ABCC9, ACADVL, ACTA1, ACTC1, ACTN2, AGK, ALMS1, ALPK3, ANKRD1, APOA1, BAG3, BRAF, CACNA1C, CACNB2, CALR3, CAV3, CHRM2, COX15, CRYAB, CSRP3, CTF1, CTNNA3, DES, DMD, DNAJB6, DPP6, DSC2, DSG2, DSP, DTNA, EMD, EYA4, FHL1, FHL2, FHOD3, FKTN, FLNC, FXN, GATAD1, GJA5, GLA, ILK, JPH2, JUP, KCNA5, KCNE2, KCNE3, KCNH2, KCNJ2, KCNJ8, KCNQ1, KRAS, LAMA4, LAMP2, LDB3, LMNA, MIB1, MRPL3, MTO1, MYBPC3, MYH6, MYH7, MYL2, MYL3, MYL4, MYLK2, MYOM1, MYOZ2, MYPN, NCF1, NEBL, NEXN, NPPA, NUP155, OBSCN, PDLIM3, PKP2, PLN, PRDM16, PRKAG2, PSEN1, PSEN2, RAF1, RBM20, RORC, RYR2, SCN1B, SCN2B, SCN3B, SCN4B, SCN5A, SCO2, SDHA, SGCD, SHOC2, SLC25A4, SNTA1, SYNE1, TAZ, TCAP, TGFB3, TMEM43, TMPO, TNNC1, TNNI3, TNNT2, TPM1, TRIM63, TTN, TTR, TXNRD2, VCL |
| **Arrhythmia genes (n=272)** | |
|  | ABCC9, ACE, ACTC1, ACTN2, ADORA1, ADORA2A, ADORA2B, ADORA3, ADRA1A, ADRA1B, ADRA1D, ADRA2A, ADRA2B, ADRA2C, ADRB1, ADRB2, ADRB3, AGT, AGTR1, AKAP10, AKAP9, ALG10, AMY2A, AMY2B, ANK2, ANKRD1, ANXA7, AR, ARNTL, ASB2, ASPH, ATP1A1, ATP1A2, ATP1A3, ATP1A4, ATP1B1, ATP2A2, ATXN1, AVP, BAG3, CACNA1C, CACNA1D, CACNA1F, CACNA1S, CACNA2D1, CACNA2D2, CACNB1, CACNB2, CACNB3, CACNB4, CACNG1, CALR, CAMK4, CASQ2, CAV3, CCL2, CDC42EP3, CHI3L1, CHRM1, CHRM2, CHRM3, CHRM4, CHRM5, CIT, CNTN5, COL3A1, COLQ, CREM, CRP, CSF3, CTNNA3, CYP2D6, DES, DMD, DMPK, DPP6, DSC2, DSG2, DSP, ENTPD1, ESRRG, F10, F2, FHL2, FKBP1B, FOXO3, FRMD4B, GHRL, GJA1, GJA5, GNAI2, GNB5, GPD1L, GRHPR, HCN1, HCN4, HEY2, HLA-B, HLA-DQA1, HMGB1, HMGCR, HRAS, HRH1, HTR2A, IL6, ILK, JUP, KCNA1, KCNA4, KCNA5, KCNA7, KCND3, KCNE1, KCNE2, KCNE3, KCNG2, KCNH2, KCNH7, KCNIP2, KCNJ11, KCNJ12, KCNJ2, KCNJ5, KCNJ8, KCNK3, KCNMA1, KCNMB1, KCNMB2, KCNMB3, KCNMB4, KCNQ1, KHDRBS1, KIF21B, KITLG, KLF3, KRAS, LAMA4, LCP2, LMNA, LRRC63, MAOA, MAOB, MIR17HG, MMP1, MMP2, MMP9, MPO, MYBPC2, MYBPC3, MYH6, MYH7, MYL4, MYOM1, NAA10, NEXN, NHLH1, NHLH2, NOS1AP, NPC1L1, NPPA, NPPB, NR3C1, NR3C2, NTRK2, NUP155, OPRM1, P2RX7, P2RY1, P2RY12, PI4KA, PIK3CG, PITX2, PKP2, PLA2G6, PLAT, PLN, PPP1CA, PPP1R13L, PPP2CA, PRKAG2, PROC, PTGFR, PTGS1, PTGS2, PTPRE, RAC1, RAF1, RANGRF, RAPGEF4, RBM20, REN, REST, RGS4, RGS6, RHOA, RIMS1, RYR2, S1PR2, SCN10A, SCN1A, SCN1B, SCN2B, SCN3B, SCN4A, SCN4B, SCN5A, SCN9A, SERINC2, SERPINC1, SERPINE1, SIRT6, SLC2A5, SLC30A5, SLC6A2, SLC6A4, SLC8A1, SLMAP, SNTA1, SOAT1, SP4, TAC1, TANGO2, TBX3, TBX5, TBXA2R, TECRL, TFPI, TGFB1, TGFB3, TGM2, THRA, THRB, TIMP1, TMEM43, TNF, TNNI3, TNNT2, TOR2A, TPM1, TRDN, TRIM21, TRIO, TRPM4, TTN, TUBA1A, TUBA1C, TUBA4A, TUBA8, TUBB1, TUBB2A, TUBB3, TUBB4A, TUBB4B, TUBD1, TUBE1, TUBG1, TUBG2, TXN, UBR4, UBR5, UCN, UCN2, UTS2, VAV2, VCAM1, VCL, VEGFA, VKORC1, VWF, WDR26, ZC3HAV1L, ZDHHC16 |
|  |  |
