# Supplementary material for: Rare Pathogenic Variants in Mitochondrial and Inflammation-Associated Genes May Lead to Inflammatory Cardiomyopathy in Chagas Disease
Source: J Clin Immunol. 2021 Mar 3;41(5):1048–63. doi: 10.1007/s10875-021-01000-y (PMC8249271; doi:10.1007/s10875-021-01000-y)
Supplement: Supplementary file 5 — (DOCX 13 kb) [file 10875_2021_1000_MOESM5_ESM.docx]

**Online Table 4: Reactome pathways analysis of the 321 CCC-specific variants**

| **Pathway name** | **#Entities found** | **#Entities total** | **Entities FDR** | **Species name** | **Submitted entities found** |  | |
| --- | --- | --- | --- | --- | --- | --- | --- |
| Endosomal/Vacuolar pathway | 14 | 82 | 4,17E+10 | Homo sapiens | HLA-C | |  |
| Antigen Presentation: Folding, assembly and peptide loading of class I MHC | 14 | 102 | 2,77E+11 | Homo sapiens | HLA-C | |  |
| Interferon gamma signaling | 21 | 255 | 1,32E-03 | Homo sapiens | HLA-DRB5;HLA-DPB1;HLA-C;HLA-DPA1 | | |
| ER-Phagosome pathway | 15 | 165 | 7,34E-03 | Homo sapiens | BTK;HLA-C | |  |
| Antigen processing-Cross presentation | 15 | 187 | 2,31E-02 | Homo sapiens | BTK;HLA-C | |  |
| Interferon alpha/beta signaling | 15 | 191 | 2,40E-02 | Homo sapiens | HLA-C | |  |
| Interferon Signaling | 23 | 401 | 4,71E-02 | Homo sapiens | NUP214;HLA-DRB5;HLA-DPB1;HLA-C;HLA-DPA1 | | |
| Nuclear Receptor transcription pathway | 9 | 86 | 4,75E-02 | Homo sapiens | NR3C1 | |  |
