# Supplementary material for: Rare Pathogenic Variants in Mitochondrial and Inflammation-Associated Genes May Lead to Inflammatory Cardiomyopathy in Chagas Disease
Source: J Clin Immunol. 2021 Mar 3;41(5):1048–63. doi: 10.1007/s10875-021-01000-y (PMC8249271; doi:10.1007/s10875-021-01000-y)
Supplement: Supplementary file 6 — (DOCX 23 kb) [file 10875_2021_1000_MOESM6_ESM.docx]

**Online table 4: Summary of all the information available for the 22 variants of interest.**

| **Fam** | **Symbol and**  **gene Name** | **Variant** | **Function** | **Effect of**  **KO/KD** | **Effect of variant** | **Clinical**  **phenotype** |  |
| --- | --- | --- | --- | --- | --- | --- | --- |
| **Mitochondrial genes** | | | | | | | |
| 5 | DHODH  Dihydroorotate dehydrogenase | R135C | Provides electrons to OXPHOS and pyrimidine biosynthesis. | Increased mtROS, reduced MMP and ATP production.  DHODH inhibitors used clinically in rheumatic disease as immune suppressors via pyrimidine starvation. | R135C  complete loss of function. | R135C variant in 6 affected families: Miller's syndrome: facial dysostosis, 30-80% congenital heart defects |  |
| 4 | UMPS  Uridine monophosphate synthetase | S30G | Pyrimidine synthesis pathway, its substrate orotate is the product of DHODH | Accumulation of orotic acid inhibits FAOx | NR | Orotic aciduria, congenital heart disease, Neurodevelopmental disorder |  |
| 4 | RPUSD3  RNA pseudoruridinate synthase 3 | W269X | Uridinates mitochondrial 16S rRNA;nmitochondrial translation | 50% reduction of mitochondrial translation. Reduction of OXPHOS. | Truncation of 25% C-terminal sequence | Not described |  |
| 3 | MRPS18B  Mitochondrial ribosomal protein 18B | V120M | Component of the small subunit of mitochondrial ribosome | Reduced mitochondrial number, morphological alterations, reduced cardiac contractility. | missense mutations in multiple MRPs lead to reduced 12S mtRNA and impaired OXPHOS function. | mitochondriopathy with cardiomyopathy |  |
| 1 | ADCY10 adenylate cyclase 10 | Y412C | pH sensor HCO3/Ca+/ATP sensor | Reduced OXPHOS activity, MMP, ATP production. | NR | - |  |
| 3 | GIT1 (ArfGAP 1)  G Protein-Coupled Receptor Kinase Interacting | A745T; R43H | Multifunctional scaffold protein, regulator of mitochondrial biogenesis, regulation of chemoattractant-induced cell motility. | Altered mitochondrial morphology  Reduced biogenesis,heart mitochondrial function. Reduced MMP and ATP production,ventricular dysfunction. | NR | - |  |
| 1 | MOCS1 molybdenum cofactor synthesis 1 | R339W | Synthesis of molybdenum cofactor important for mitochodrial and cytoplasmic proteins. |  | R67W, G126D. Altered OXPHOS, reduced ATP, mitochondrial dynamics, fragmentation, increased ROS. | Molybdemum deficiency 1: neonatal encephalopathy |  |
| 2 | OBSCN  Obscurin | G2113C | Maintains precise relationships between myofibrils, mitochondria, and sarcoplasmic reticulum .  Important for fast delivery of Ca^2+^ and ATP for contraction. |  |  | Hyperptrophic, dilated, noncompaction cardiomyopathy |  |
|  |  |  |  |  |  |  |  |
| **Inflammatory genes** | | | | | | | |
| 1 | ADGRG6  Adhesion G protein coupled receptor G6 | A625T | Scaffold protein.  Induced by LPS via MAP Kinases.  Involved in adhesion, neuronal myelinization, and cardiac development. |  | NR |  |  |
| 1 | AKAP13  A-kinase anchoring protein 13 | L1132S | Scaffold protein.  Inducible by TLR2 ligands, activates NF-kB and MAP kinases inducing inflammatory cytokines.  (AT1Rs),and hypertrophic from beta-adrenergic receptors. Heart failure. | Reduces TLR2-mediated NF-kappa B activation and production of IL-8 and MCP-1. | NR |  |  |
| 3,6 | APOB  Apolipoprotein B | D749Y (fam 3)  N3107K (fam 6) | Lipid carrier in plasma. Activates macrophage NF-kB and MAP kinase pathways leading to increased production of inflammatory cytokines. Protects cells from lipotoxicity. | Overexpression blocks lipid accumulation and heart failure in diabetic mice | NR | Familial hypercholesterolemia |  |
| 1 | LEPR  Leptin Receptor | T699M | Hub between metabolism and inflammation.  Activates monocyte production of proinflammatory cytokines and increased TLR2 expression.  Induces Th1 polarization and IFN-gamma production. Suppresses regulatory T cells. Leptin increases FAOx and protects from lipotoxicity. | db/db mice (LEPR mutant) show reduced FAOx, develop myocardial lipid accumulation and dysfunction. | NR | Severe obesity |  |
| 3 | LILRA2  Leukocyte immunoglobulin like receptor A2 | R423C | Innate receptor.  Activates cytokines IL6, IL8, MIP-1alpha, TNF-alpha. Activates NF-kB and MAPK pathways.  Promotes Th17 differentiation. |  |  |  |  |
| 4 | MAP4K4  Mitogen-activated protein kinase kinase kinase kinase 4 | K91E | Activates MAPK8/JNK. Important for LPS-induced production of TNF-alpha and IL1-beta. | inhibits LPS-induced production of TNF-alpha ant IL1-beta |  |  |  |
| 4 | SLC11A1 (NRAMP)  Solute carrier family 11 member 1 | R397C | Macrophage activator and endosomal iron exporter. Induclble by IFNgamma, activates nuclear translocation of NF_kB and STAT1 and upregulation of cytokines TNFalpha and IL-1 beta. |  |  |  |  |
| 5 | MAML1  Mastermind like transcriptional coactivator 1 | G136E | Transcriptional coactivator of the NOTCH pathway.  Involved in TNF-alpha signal transduction to NF-kB. Enhances TNF-alpha cytotoxicity. | impaired TNF-alpha induced NFkB responses. |  |  |  |
| 6 | TNFRSF4  Tumor Necrosis Factor Receptor Superfamily Member 4,  OX40 | D163E | Induces macrophage secretion of proinflammatory cytokines. Activates NF-kB in T cells. Costimulatory molecule involved in long-term T cell memory to viral infection. Increases Th1 and Th17 polarization. |  |  | T cell immunodeficiency |  |
| **Other** | | | | | | | |
| 6 | SERPINE2  Serpin Peptidase Inhibitor Clade E member 2 | M64T | Matrix remodeling protein, upregulated in failing heart. Induces collagen deposition |  |  |  |  |
| 3 | PKHD1 Fibrocystin/  polyductin | T36M | important for structural integrity of kidney and liver. Leads to pericystic fibrosis and congenital hepatic fibrosis. |  |  | Autosomal Recessive Polycystic Kidney Disease 4 |  |
| 3 | RNLS  Renalase | R222H | Modulates sympathetic tone. Induced by high concentrations of catecholamines.  Degrades circulating catecholamines.  Lowers blood pressure and decreases cardiac contractility. | increased catecholamine levels, tachycardia, hypertension |  |  |  |
