# Supplementary material for: Rare Pathogenic Variants in Mitochondrial and Inflammation-Associated Genes May Lead to Inflammatory Cardiomyopathy in Chagas Disease
Source: J Clin Immunol. 2021 Mar 3;41(5):1048–63. doi: 10.1007/s10875-021-01000-y (PMC8249271; doi:10.1007/s10875-021-01000-y)
Supplement: Supplementary file 7 — (DOCX 13 kb) [file 10875_2021_1000_MOESM7_ESM.docx]

**Online table 5: Frequency of our variants of interest in latino reference subpopulations.**

| **Family** | **Gene** | **Amino acid** | **avsnp147** | **1000G** | **1000G** | **1000G** | **1000G** | **ExAC** |
| --- | --- | --- | --- | --- | --- | --- | --- | --- |
|  |  | **change** |  | **PUR** | **MXL** | **CLM** | **PEL** | **Latino/Admixed American** |
| **1** | LEPR | T699M | rs34499590 | 0 | 0 | 0,005 | 0 | 0 |
| **1** | ADCY10 | Y402C | rs140663029 | 0 | 0 | 0 | 0 | 0,0018 |
| **1** | MOCS1 | R339W | rs148579886 | 0 | 0 | 0 | 0 | 0,0006 |
| **1** | ADGRG6 | A625T | rs184235213 | 0,01 | 0 | 0,005 | 0 | 0,0006 |
| **1** | AKAP13 | L1132S | rs745783128 | NA | NA | NA | NA | NA |
| **2** | OBSCN | G2113C | rs74623201 | 0 | 0 | 0 | 0 | 0,019 |
| **3** | APOB | D749Y | . | NA | NA | NA | NA | NA |
| **3** | MRPS18B | V120M | rs116524936 | 0 | 0 | 0 | 0 | 0,00003 |
| **3** | PKHD1 | T36M | rs137852944 | 0 | 0 | 0 | 0 | 0 |
| **3** | RNLS | R222H | rs191733133 | 0 | 0 | 0,005 | 0 | 0,00006 |
| **3** | GIT1 | A745T | . | NA | NA | NA | NA | NA |
| **3** | GIT1 | R43H | . | NA | NA | NA | NA | NA |
| **3** | LILRA2 | R423C | rs149580797 | 0,005 | 0 | 0 | 0 | 0,0005 |
| **4** | MAP4K4 | K91E | . | NA | NA | NA | NA | NA |
| **4** | SLC11A1 | R397C | rs74906275 | 0 | 0 | 0 | 0 | 0,0001 |
| **4** | RPUSD3 | W269X | rs142984515 | NA | NA | NA | NA | NA |
| **4** | UMPS | S30G | rs17843776 | 0 | 0,031 | 0,011 | 0,029 | 0,028 |
| **5** | MAML1 | G136E | rs146382198 | 0 | 0,008 | 0 | 0,018 | 0,005 |
| **5** | DHODH | R135C | rs201230446 | 0 | 0 | 0,005 | 0 | 0,0002 |
| **6** | TNFRSF4 | D163E | . | NA | NA | NA | NA | NA |
| **6** | APOB | N3107K | rs72653101 | 0 | 0 | 0 | 0 | 0,0001 |
| **6** | SERPINE2 | M64T | rs34078713 | 0,019 | 0,008 | 0,027 | 0 | 0,007 |
